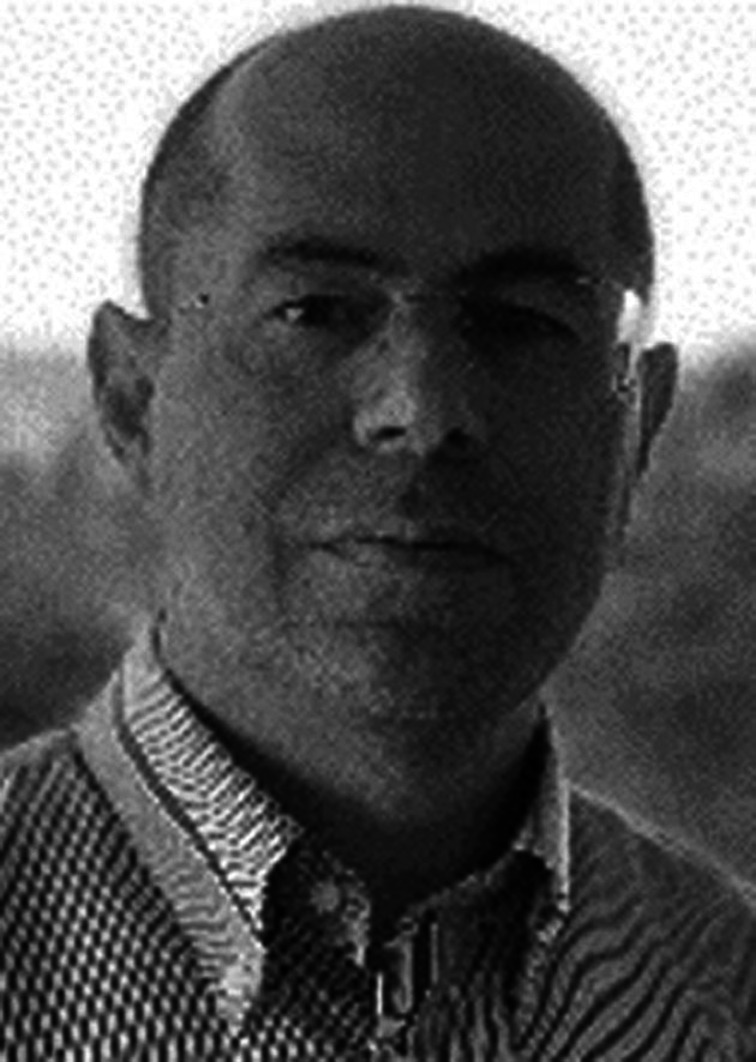# Mechanochemistry: New Tools to Navigate the Uncharted Territory of “Impossible” Reactions

**DOI:** 10.1002/cssc.202200362

**Published:** 2022-07-21

**Authors:** Federico Cuccu, Lidia De Luca, Francesco Delogu, Evelina Colacino, Niclas Solin, Rita Mocci, Andrea Porcheddu

**Affiliations:** ^1^ Dipartimento di Scienze Chimiche e Geologiche Università degli Studi di Cagliari Cittadella Universitaria 09042 Monserrato, Cagliari Italy; ^2^ Dipartimento di Chimica e Farmacia Università degli Studi di Sassari via Vienna 2 07100 Sassari Italy; ^3^ Dipartimento di Ingegneria Meccanica, Chimica e dei Materiali Università degli Studi di Cagliari Via Marengo 2 09123 Cagliari Italy; ^4^ ICGM Univ Montpellier, CNRS, ENSCM Montpellier France; ^5^ Department of Physics Chemistry and Biology (IFM) Electronic and Photonic Materials (EFM) Building Fysikhuset, Room M319, Campus Valla Sweden

**Keywords:** ball milling, green chemistry, Grignard reactions, mechanochemistry, mechano-redox reactions

## Abstract

Mechanochemical transformations have made chemists enter unknown territories, forcing a different chemistry perspective. While questioning or revisiting familiar concepts belonging to solution chemistry, mechanochemistry has broken new ground, especially in the panorama of organic synthesis. Not only does it foster new “thinking outside the box”, but it also has opened new reaction paths, allowing to overcome the weaknesses of traditional chemistry exactly where the use of well‐established solution‐based methodologies rules out progress. In this Review, the reader is introduced to an intriguing research subject not yet fully explored and waiting for improved understanding. Indeed, the study is mainly focused on organic transformations that, although impossible in solution, become possible under mechanochemical processing conditions, simultaneously entailing innovation and expanding the chemical space.

## Graphical abstract

**Mission impossible**: Mechanosynthesis is erroneously perceived as an enabling tool for only making greener *well‐known* solution‐based processes. Modern mechanochemistry is, above all, an effective tool to open synthetic paths not accessible by following conventional and well‐known pathways already reported in the literature for the classical chemistry in solution. Mechanical stimuli promote uncharted reaction pathways, making it possible to design and develop effective, successful synthetic protocols previously classified as “impossible reactions”.

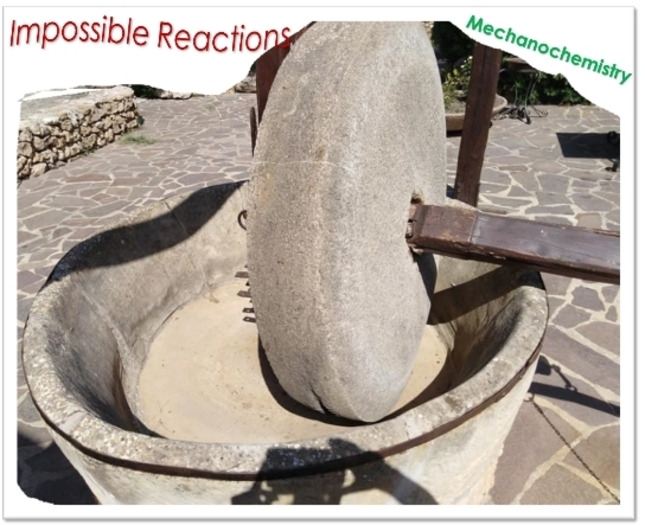

## 1 Introduction

**Figure 1:**
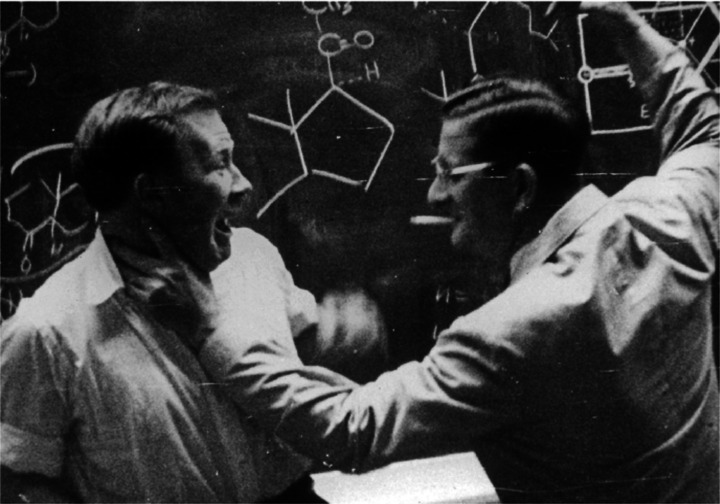
William E. Moffitt and Robert B. Woodward in front of a blackboard. Photography reproduced by courtesy of Wiley‐VCH.^[1]^

A famous photograph pictures the renowned organic chemist, Nobel laureate Robert B. Woodward having fun with his younger colleague William E. Moffitt in front of a blackboard filled with chemical structures and reactions. What immediately catches the chemist's eye in the photo shown in Figure 1 is the absence of any reference to the solvent that should dissolve the chemical species and allow their chemical combination. And yet, there is no textbook, scientific publication, or chemistry teacher that does not emphasize the importance of choosing the right solvent to perform a chemical synthesis with a reasonable hope of controlling its kinetics and maximizing its yield and selectivity.

Could an organic chemist even imagine carrying out his syntheses without resorting to his beloved solvents? Not that the solvent is free from drawbacks. Besides the initial, careful choice, it weighs down the chemist's work, imposing, for instance, time‐demanding, if not challenging, removal, purification, and crystallization steps.

Nevertheless, the current chemistry paradigm assumes that molecules must be dissolved in a solvent to get closer to each other through diffusion in the liquid and make their functional groups interact properly within the solvent cage. Indeed, it is a common experience that mixing two solid reactants, even if finely divided in the form of powder, does not suffice to make the reaction occur. It generally stops at interfaces as reaction products are not removed.

However, what may seem impossible to a classical organic chemist can appear in a whole new light to a mechanochemist. Accustomed to invoking Hephaestus’ mastery and power to strike and shape metals and hard ceramics in his laboratory, the mechanochemist knows how mechanical forces can effectively blend powders and mix chemical species on the molecular scale, easily overcoming the generally weak resistance to deformation of most organic crystals.

There are enormous and far‐reaching consequences that only recently have been highlighted in the community of organic and inorganic synthesis. Mechanochemistry^[2]^ can significantly benefit classical solution chemistry and supramolecular chemistry, providing alternative routes to known chemicals and completely new synthetic strategies for novel chemical compounds. The capacity to enable solvent‐less reactions, or, at least, to significantly reduce the amount of solvent utilized, is especially significant in the present period, characterized by increasing awareness of environmental issues and a renewed attention to pollution and climate change.

## 2 Mechanochemistry vs. Solvent‐Based Chemistry

Although introduced by W. H. Ostwald at the end of the 19th century, mechanochemistry has been often regarded as exotic chemistry. This is beginning to change. In this context, the remarkable papers of Senna^[3]^ and Toda et al.^[4]^ could be regarded as a milestone in the field of modern mechanosynthesis. In one of his papers, Senna's statement is as relevant today as ever. Many years ahead of time, their research has anticipated several theories and assumptions that we take for granted today. Back in the 1990s,[^4a^, ^4b^, ^4c^] these studies showed that mechanochemically promoted reactions proceed along synthetic pathways different from those described in the solution. Shan et al. successfully prepared several co‐crystals by mechanochemical activation for the first time.^[4e]^ Due to redox isomerization processes, these same compounds cannot be prepared following classical solvent‐based procedures. Moreover, Toda and co‐workers observed that the grinding of an aromatic aldehyde and acetophenone in the presence of NaOH led to the corresponding chalcone within a few minutes.^[4d]^ In the same reaction time, the target reaction performed in the presence of a solvent yielded mainly the aldol addition product.

Recent years have seen a remarkable surge of interest in mechanochemistry,^[5]^ turning this subject into one of the most vibrant fields of study at the cutting edge of scientific research in chemical sciences.^[6]^

**Figure 2:**
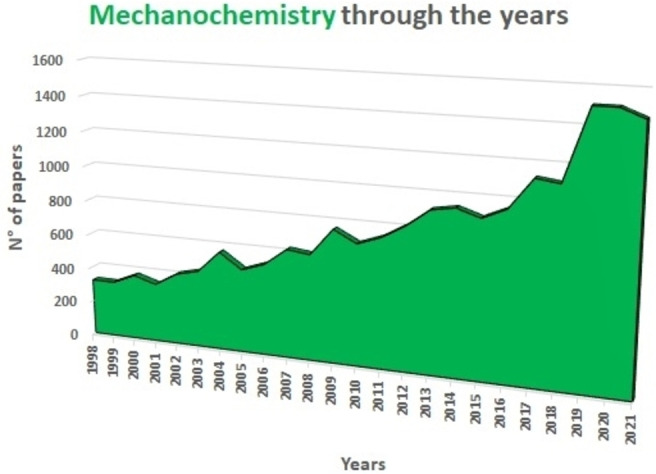
Number of papers on mechanochemistry per year. Data extracted from Sci‐finder database Number of papers on mechanochemistry per year. Data extracted from Sci‐finder database, including patents, using “mechanochemistry” as keyword.

Highlighted by the exponential growth of dedicated publications (Figure 2),^[7]^ the interest attracted by mechanochemical methods finds its explicit recognition in the attention of scientific journals and mass media. Thus, it is no coincidence that the IUPAC has included mechanochemistry among the top 10 technologies that can change our world.^[8]^

Presently, the scientific community revolving around mechanochemistry, strengthened by the ongoing EU COST Action CA18112 Mechanochemistry for Sustainable Industry,^[9]^ displays a rare multidisciplinary nature. Researchers from many different areas of physics, chemistry, and engineering strive together to meet the demand for deeper understanding and full exploitation of mechanochemical methods.^[10]^ Under the push of the more sensitive industrialists’ and entrepreneurs’ call for greener solutions to chemical manufacturing, entirely new paths have been opened. Particularly meaningful in this regard is the alternative preparation of active pharmaceutical ingredients (APIs)^[11]^ and solid pharmaceutical forms (referred to as medicinal mechanochemistry) by both batch^[12]^ and continuous^[13]^ mechanochemical processing suitable for scale‐up.^[ 14]^

**Figure 3:**
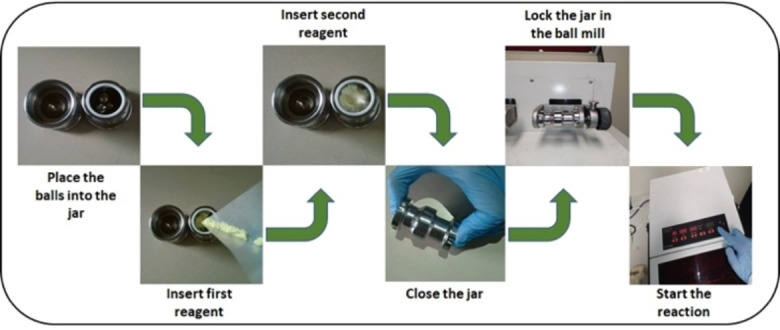
Practical aspects of setting up a mechanochemical reaction in a ball mill.

The experimental apparatuses utilized to activate and drive mechanochemical transformations^[15]^ contribute to making mechanochemistry appealing. Easy to use and characterized by straightforward and versatile design (Figure 3), ball mills and extruders can be regarded as the evolution of mortar and pestle,^[16]^ a ubiquitous tool throughout the history of human beings. Crushing, blending, rubbing, and mixing are no longer entrusted to the hands of researchers but to a set of grinding bodies that eventually make powders undergo a series of compression and shear processes. Efficient and practical, the modern tools display their power in various mechanical processing solutions and countless successful synthetic routes to chemicals and new materials. Thus, the whole spectrum of educational^[17]^ and production purposes is covered from laboratory to industrial scale.

However, despite the apparent simplicity of the experimental set‐up, mechanochemistry hides many levels of complexity,^[18]^ showing a unique capacity to enable unconventional processes challenging to imagine before their demonstration and challenging to seize after. Unquestionably, the initiation of chemical reactions in the solid state under solventless conditions^[19]^ can be counted among the most exciting opportunities mechanochemical methods offer.

Given these interesting premises, we have often wondered whether mechanochemistry is “another passing fashion” or if it is a golden opportunity to design new synthetic strategies following unconventional chemical approaches never explored before in the presence of organic solvents.^[20]^ In order to provide appropriate answers to these questions, it is crucial to focus on some of the strengths of this new technology that have dictated its success today.

As mechanochemical processes can occur without solvents^[21]^ or in the presence of only catalytic amounts of a solvent phase, in a process known as liquid‐assisted grinding (LAG),^[22]^ mechanochemistry has every right to be classified as green chemistry.[^7c^, ^15^, ^23^] In this respect, evidence removes any doubt. Moreover, a definite improvement of green metrics accompanies the adoption of mechanochemical synthetic routes, mainly when pursued under continuous processing conditions. In addition, it does not suffer from the drawbacks displayed by solvent‐free reactions performed under annealing conditions,^[24]^ which often lead to complex mixtures of compounds that require tedious chromatographic purification.^[25]^

In a chemical process, solvents are the majority components, often making up over 90 % of the reactant mass.^[26]^ Unfortunately, most organic solvents used in chemical reactions are not environmentally friendly^[27]^ and tend to raise significant concerns for public health and the environment.^[28]^ In addition, their production, transportation, and storage add further issues in terms of economics and safety.^[29]^

Last but not least, the heating and cooling of these masses, mainly when operating on an industrial scale, involve high additional costs, further accenting the problems highlighted above.^[30]^ Moreover, the need to work under inert reaction conditions^[31]^ using degassed^[32]^ and anhydrous solvents^[33]^ is another often‐underestimated aspect of solvent‐based chemistry that drastically increases cost production, resulting in a more significant environmental footprint.

Unfortunately, the problem cannot be addressed quickly by wiping away the solvent since the chemical equation is not a simple algebraical manipulation.^[34]^ In this context, mechanochemistry has brought innovative and pioneering solutions to bridge some of the most challenging issues faced with solvent‐less reactions.^[5a]^

Such development is very attractive because of more efficient and cleaner pathways for exhaustive or site‐selective derivatization of small molecules, particularly for pharmaceutical companies.^[35]^

However, significant opportunities are often combined with great challenges, and mechanochemistry is no exception. Understanding mechanochemical transformations is a problematic task precisely because of the profound differences between conventional solution chemistry and the chemistry activated by mechanical forces.^[36]^

**Figure 4:**
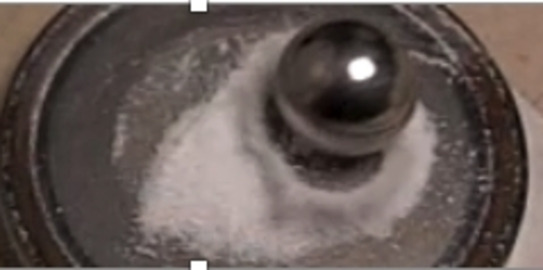
A frame of the impact between ball and jar wall.

First and foremost, mechanical processing has discrete nature, and correspondingly discrete are the resulting physical and chemical transformations.^[37]^ Time by time, the applied mechanical stresses reach the intensity needed to activate the reaction only in a tiny fraction of the processed sample. The remaining volume is left unaffected (Figure 4).

Second, mechanochemical reactions involving two or more species heavily depend on the generation of interfaces between the solids. This process replaces the mediating role of the solvent, which allows individual molecules to diffuse and come in contact within a fluid matrix. Therefore, dimensionality is reduced from three to two, and chemical interaction is only possible through the interaction of molecular reactants at interfaces. It follows that the mechanical response of the different solid phases plays a crucial role in determining the reaction rate.

Third, the reactivity of covalent bond systems can be affected and probably enhanced by mechanical forces and shear stresses. Individual molecules, or small molecular clusters, can be forced into high‐energy local configurations with elongation of chemical bonds, making them more prone to a chemical reaction. Not to mention the effects of forced mixing, which can result in the extraction of individual species from the parent phase and their implantation in a different one.

Fourth, mechanical stresses necessarily superpose to thermal effects. The most straightforward consequence is that compression and shear can occur at relatively high local temperatures upon relaxing severely deformed local states and the corresponding heat release. The resulting processing conditions can be quite extreme for several organic compounds, and a significant enhancement and diversification of chemical reactivity can be expected accordingly.

Herein, grinding, impacting, crushing, pulverizing, and shearing forces can induce a chemical reactivity different from thermal or photochemical processes.^[38]^ These force‐induced perturbations can change the energy landscape of chemical reactions and accelerate the dissociation of unloaded bonds and the changesets in the electronic configuration of covalent bonds, enabling a different chemical selectivity than conventional solution‐based reactions.^[39]^

Strong impact forces are responsible for two dominant features during ball milling: crystal breaking and defect formation, which (especially the latter) in turn affect the chemical reactivity of the mechanochemical processes, increasing the chemically active surface area and enhancing better interaction with reactants.^[40]^ Moreover, in contrast to the chemical process in solution, the high‐energy mechanical action shifts the atoms involved in the reaction away from their equilibrium position, promoting the reaction product‘s irreversible and high‐yield formation.^[41]^ Indeed, by managing some milling parameters, for instance, the ball/powder weight ratio, milling speed, milling time, and atmosphere, it is also possible to drive the kinetics of mechanochemical reactions. Sometimes, many chemical reactions^[42]^ involving solid reactants have high activation energies.

Despite being subjected to high‐energy impacts within the jars, these mechanical forces cannot provide the required energy. However, the mechanical action prolonged over time on the powder particles generates a large surface area and intimate mixing between reactants, thus lowering the energy barrier of the transition state, which somewhat explains the success of mechanochemical processes. The mechanochemistry freed from the strings and snares imposed by choice of solvent and with such a winning combination of features (creation of active sites, generation of new functional surfaces, and lowering of activation energy) allows the design of new scenarios and synthetic paths in areas completely hidden to traditional chemistry in solution.

In addition, mechanochemistry has several unique mechanistic features that also help to cut down on reaction times, making it an environmentally friendly, more cost‐effective, and less polluting process.^[43]^

None of the features mentioned above finds a counterpart in conventional solution chemistry.^[44]^ This is a continuous form of chemistry governed by common mass, momentum, and energy transport phenomena. Most of the theoretical achievements allowing a satisfactory description and comprehension of chemical events in solution do not apply to mechanochemistry. This is, indeed, a form of chemistry where global kinetics is the result of intimately intertwined statistical, mechanical, rheological, and chemical factors.

All of this brings with it several implications, the most important of which is probably that mechanochemical methods cannot be simply regarded as a tool to implement, under solvent‐free conditions, the usual chemical transformations allowed by solution processes. It would be an incomprehensible underestimation and a potentially dangerous mistake.

Although mechanochemistry took a long time to be given the same dignity as other chemistry forms, it is a chemical science with highly specific features and potential. Our knowledge, limited as it is, makes us perceive since long ago how peculiar the chemistry activated and driven by mechanical forces is. It offers an entirely new range of solutions to the chemical combination. Externally driven transformations can be obtained along with unexplored directions, precisely directed to where conventional chemistry surrenders and establishes the borders between what is possible and impossible.

It is precisely here that our work finds its place and motivation. Setting aside all self‐generated hesitation, mechanochemistry should be valued for what it really is, namely a chemical science offering the rare opportunity of opening alternative synthetic paths to fine chemicals and pharmaceuticals as well as to advanced materials, providing access to otherwise unreachable targets while challenging our fundamental understanding of chemistry as a whole. In short, mechanochemistry is a challenging opportunity to explore hitherto uncharted territories of chemical space.

Crushing, rubbing, grinding, and all the different possible combinations of compression and shear can give access to new paths in the energy landscape of chemical reactions, resulting in a chemical reactivity utterly different from the known one. This is true for solventless processes and LAG.^[45]^

Within this framework, we address the ability of mechanochemical methods to give rise to chemical reactions that are very difficult, or even impossible, to be carried out under conventional solution‐based processing conditions. Thus, far from being exhaustive, our overview aims at capturing at least the most significant pictures from a region of chemistry not yet adequately investigated, and at drawing attention from the ever‐growing scientific community interested in mechanochemistry and its unanswered questions.

In this scenario, we intend to provide an updated perspective on mechanically activated reactions that will be difficult or even impossible to achieve in traditional solvent‐based chemistry, thus offering the reader an insight into the potential of this technique. This research is mainly addressed to those who, for the first time, are interested in mechanochemistry and aim to capture a series of frames of an area of chemistry not yet wholly explored and with many unanswered questions.

### 2.1 Mechanochemistry for N−C, N−S, and C−C bond formation

**Figure 5:**
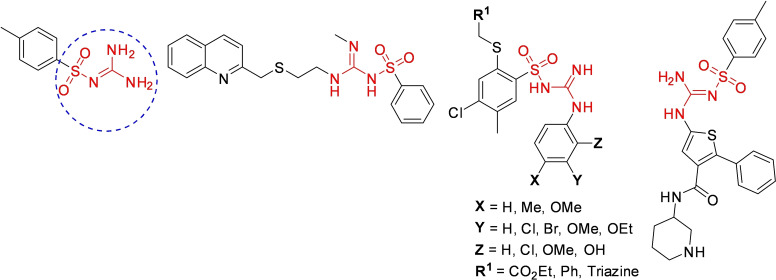
Sulfonylguanidines with potential pharmaceutical applications.

*N*‐sulfonylguanidines are a relevant class of molecules with great potential as herbicides and pharmaceuticals (Figure 5).^[46]^

**Scheme 1:**
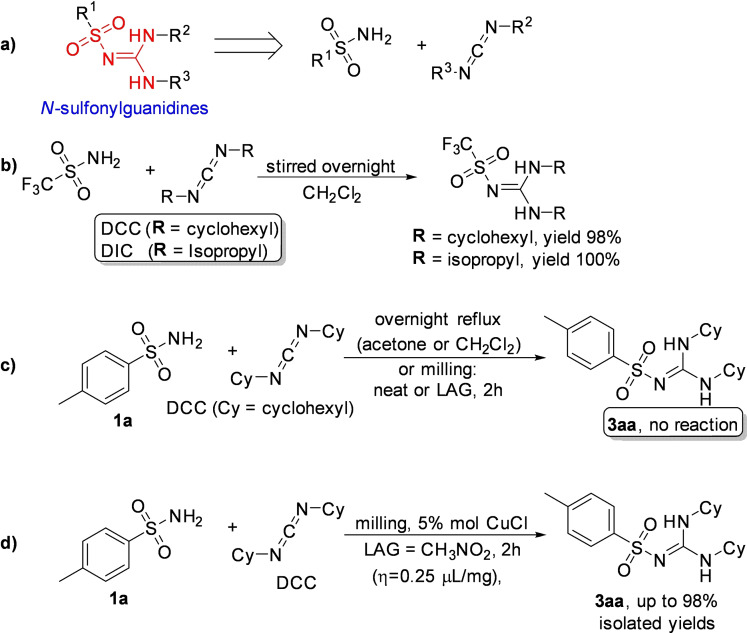
(a) A possible retrosynthetic approach to *N*‐sulfonylguanidines. (b) Reported coupling of trifluoromethylsulfonamide with DCC and DIC *N,N*′‐Diciclohexylcarbodiimide (DCC) and *N,N′*‐Diisopropylcarbodiimide (DIC). (c) First attempts to react **1 a** and DCC by solution synthesis, neat milling, or LAG. (d) Successful LAG synthesis catalyzed by 5 mol % CuCl. Adapted with permission from Ref. [47a]. Copyright 2014, Wiley‐VCH.

The retrosynthetic analysis shows that this family of compounds could be prepared by catalytic coupling of sulfonamides and carbodiimides (Scheme 1a). However, such coupling, except for trifluoromethylsulfonamide (Scheme 1b), is not usually observed in the solution synthesis because of poor sulfonamide nucleophilicity (Scheme 1c).

Friščić and co‐workers^[47a]^ used mechanochemistry to efficiently overcome the shortcomings of this reaction, developing a previously unknown copper‐catalyzed route to *N*‐sulfonylguanidines from arylsulfonamides and carbodiimides (Scheme 1d). The reaction occurs only in the presence of CuCl (5 mol %) and a substoichiometric amount of nitromethane (LAG, *η*=0.25 μL mg^−1^) (Scheme 1d). The symbol η refers to a conventionally established parameter, which corresponds to the volume of the added liquid (μL) per total mass of all reagents (mg).

As noted above, another way of thinking about facilitation for activating a chemical reaction lies in LAG.^[48]^ Although it is a common belief that using a LAG technique is recovering a solution‐mediated process, this is not exactly true. The addition of a small quantity of a solvent, in the order of 0–2 μL mg^−1^, so far from the solution value of > 12 μL mg^−1^, seems not to impact the solubilization of the compounds.^[49]^ The role that, in this case, solvents appear to play is very flexible and variable.[^49^, ^50^] In some cases, experimental data demonstrated that it could change the reactivity of some compounds; in some other cases it enhances the selectivity.^[51]^ In other ones, it plays a crucial role in the happening of the reactions.^[52]^

**Scheme 2:**
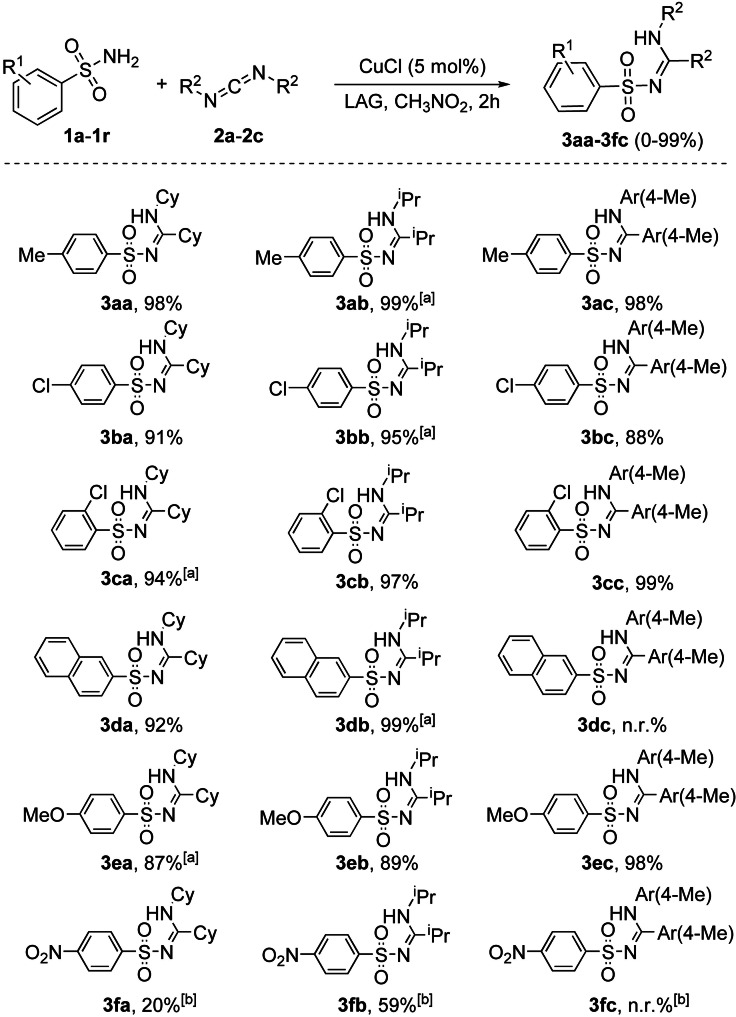
Mechanochemical CuCl‐catalyzed coupling of commercially available arylsulfonamides **1 a**–**1 r** with carbodiimides **2 a**–**2 c**. [a] Used 10% CuCl. [b] Used 20% CuCl. Adapted with permission from Ref. [47a]. Copyright 2014, Wiley‐VCH.

In the same study, the authors developed a simple and efficient solvent‐free protocol to purify the resulting *N*‐sulfonylguanidines. The reaction‘s progress can be easily monitored by Fourier‐transform infrared attenuated total reflectance (FTIR‐ATR) spectra following the disappearance of the carbodiimide signal. This mechanochemical copper‐catalyzed coupling was successfully extended to other commercially available arylsulfonamides (Scheme 2). Noteworthy, any attempt to conduct the copper‐catalyzed coupling reaction in CH_2_Cl_2_ or acetone solution failed to provide the product, even after overnight reflux, and the reactant **1 a** was retrieved. The authors speculated that the sulfonamide–carbodiimide coupling is extensively promoted in this mechanochemical process due to a higher effective concentration of the catalyst in the absence of bulk solvent.

**Scheme 3:**
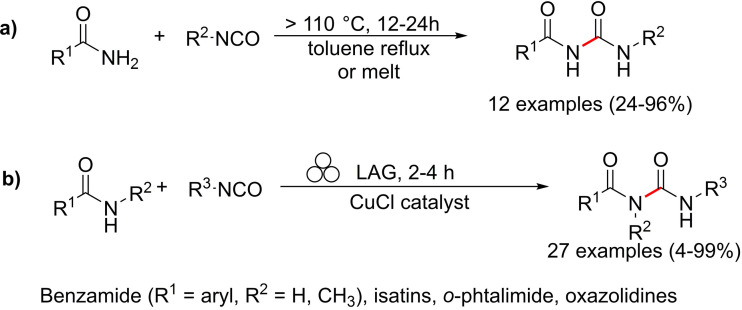
Amide coupling with isocyanates. (a) Previously reported high‐temperature procedures in solution. (b) Neat/LAG mechanochemical methodology. Adapted with permission from Ref. [55a]. Copyright 2020, Wiley‐VCH.

Mechanochemistry can also be considered a valuable means for synthesizing previously inaccessible molecules, offering transformations and selectivities that cannot be met in solution. For example, applying C−N coupling to amide substrates allows for a fast and easy way to carbamoyl amide moieties relevant in insecticides (Flucycloxuron, Triflumuron)^[53]^ and pharmaceuticals (Cefoperazone, Cabergoline, Glimepiride).^[54]^ However, in solution, although many strategies were developed to synthesize and modify amides, their derivatization is significantly less efficient and often fails to occur (Scheme 3a).

Friščić and co‐workers^[55]^ have been interested in this topic for years and recently developed a valuable mechanochemical strategy to synthesize carbamoyl isatins in 56–99 % conversion or yield carbamoyl benzamides in 4–81 % yield and carbamoyl imides in 44–92 % yield (Scheme 3b).

**Scheme 4:**
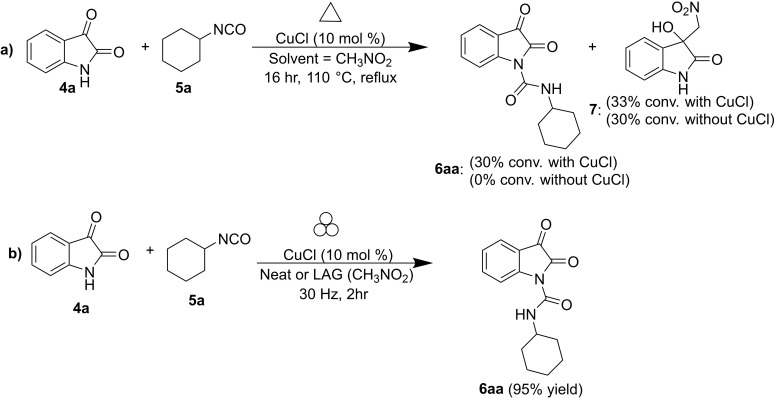
(a) Solution‐based and (b) mechanochemical copper‐catalyzed coupling of isatin with cyclohexyl isocyanate (CyNCO). Adapted with permission from Ref. [55a]. Copyright 2020, Wiley‐VCH.

This mechanosynthesis involved a rapid copper‐catalyzed coupling of isatins and benzamides, as well as a nonaromatic imide and phthalimide, with isocyanates forming the corresponding carbamoyl amides. However, in solution, the direct C−N coupling of amides with isocyanates to form carbamoyl amides required prolonged exposure (usually overnight) to temperatures higher than 110 °C in a high‐boiling solvent, giving poorer conversions (Scheme 4).

It is noteworthy to observe how the model reaction between isatin (**4 a**) and cyclohexyl isocyanate (**5 a**) (CyNCO) in hot nitromethane led to the partial formation of **6 aa** and significant amounts of a byproduct **7** from the competing Henry addition with the solvent (Scheme 4a). Such byproducts were not detected with LAG in the presence of nitromethane, proving a significant difference between thermal reactivity in solution and LAG mechanochemistry (Scheme 4b). The authors assume that a higher liquid/reactant ratio can account for the reduced performance in the solution (around 200‐fold, *η*=55 μL mg^−1^) than ball milling (*η*=0.25 μL mg^−1^).

**Figure 6:**
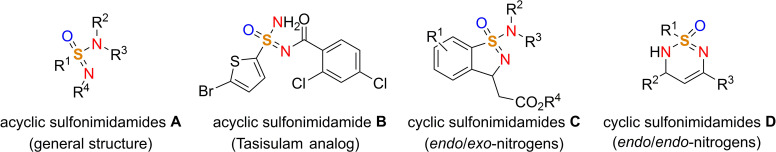
Various sulfonimidamide derivatives. Adapted with permission from Ref. [58]. Copyright 2021, American Chemical Society.

Sulfonimidamides have recently attracted increasing interest for their outstanding chemical properties as mono aza analogues of sulfonamide, in which a nitrogen atom has replaced the oxygen atom (Figure 6).^[56]^ This exchange of heteroatoms allows inserting additional molecular diversity on the N‐atom in the sulfonamide framework.^[57]^ In contrast to their achiral sulfonamide analogues, the cyclic derivatives of sulfonimidamides can be considered three‐dimensional heterocycles containing stereogenic sulfur atoms.

**Scheme 5:**
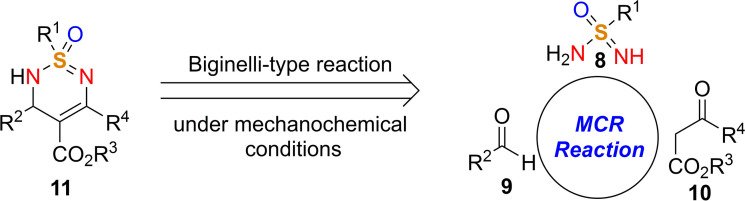
Potential retrosynthetic route to cyclic sulfonimidamide derivatives. Adapted with permission from Ref. [58]. Copyright 2021, American Chemical Society.

In this context, Bolm and co‐workers^[58]^ have developed a mechanochemical protocol to prepare cyclic sulfonimidamide derivatives^[59]^ by MCR (MultiComponent Reaction)‐type Biginelli reactions starting from easily accessible reagents (Scheme 5).

The mechanochemical MCR‐type Biginelli process has been optimized in a planetary ball mill, providing 2,3‐dihydro‐1,2,6‐thiadiazine 1‐oxide (**11 aaa**) in 98 % yield and with a diastereomeric ratio (d.r.) of 8 : 1 only in the presence of acetic acid (0.5 equiv.) and silica (60 mg, 3‐fold, Table 1). Comparable results were also achieved in the presence of Yb(OTf)_3_ (10 mol %), whereas in the absence of silica, no reaction occurred (Table 1, entries 3 and 10). Noteworthy, any attempt to develop the same MCR‐process in solution with 2 equiv. of acetic acid in refluxing ethanol for 24 h failed to provide the desired product **11 aaa**, even in trace amounts (Table 1, entry 14).

**Table 1:** Optimization of the reaction conditions.^[a]^ Adapted with permission from Ref. [58]. Copyright 2021, American Chemical Society.

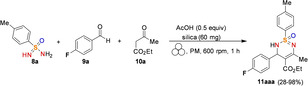		
Entry	Deviation from standard conditions	**11 aaa** yield^[b]^ [%]
1	none	98
2	no silica, no acid	n.r.
3	no silica	28
4	no acid	28
5	NaCl instead of silica	51
6	Na_2_SO_4_ instead of silica	69
7	30 mg of silica	89
8	100 mg of silica	88
9	NH_4_Cl instead of AcOH	93
10	Yb(OTf)_3_ (0.1 equiv.) instead of AcOH	91
11	Cs_2_CO_3_ instead of AcOH	92
12	39 balls instead of 20	95
13	0.20 mmol scale	89^[c]^
14	reflux in EtOH, AcOH (2.0 equiv.), 24 h	n.r.

[a] Reaction conditions: **8 a** (0.10 mmol), **9 a** (0.17 mmol), **10 aaa** (0.15 mmol), planetary ball mill (PM) with ZrO_2_ Mg‐stabilized jars (size: 12 mL) and 20 ZrO_2_ Y‐stabilized balls (size: 5 mm). [b] Determined by quantitative ^19^F NMR spectroscopy [standard: 4,4′‐bis‐(trifluoromethyl)benzophenone]. [c] Yield after flash column chromatography. n.r.=no result.

**Scheme 6:**
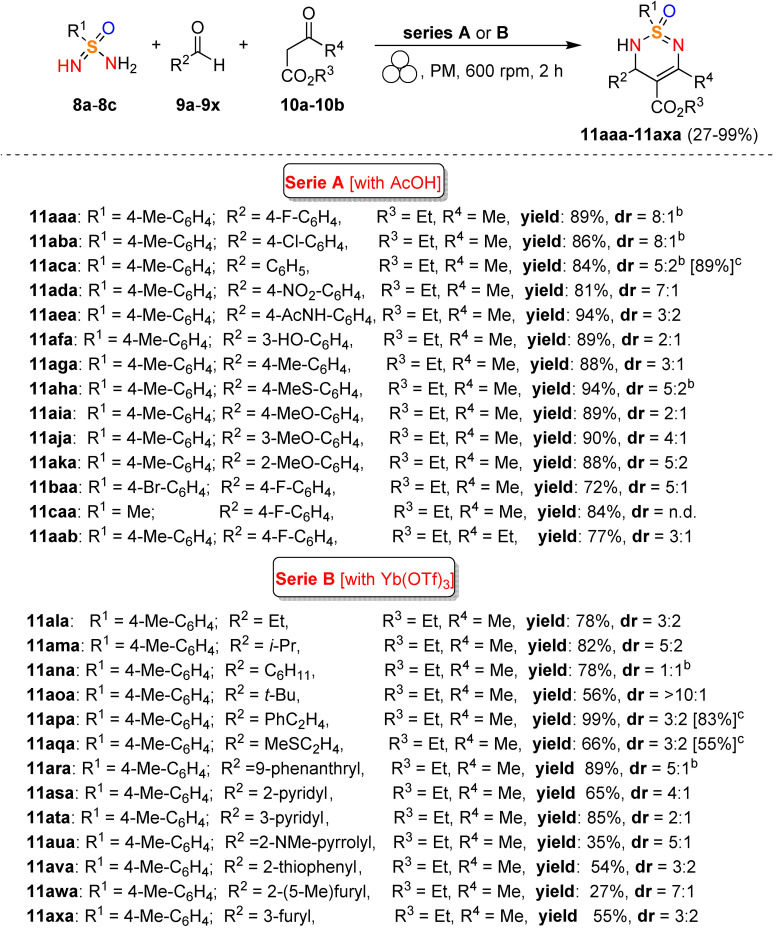
Substrate scope.^[a]^ [a] Reaction conditions: **8** (0.20 mmol), **9** (0.34 mmol), **3** (0.30 mmol), silica (200 mg); series A: AcOH (0.5 equiv.); series B: Yb(OTf)_3_ (0.1 equiv.). [b] Chromatographic separation of the diastereomers. [c] Results of reactions on a 1.0 mmol scale. n.d.=not determined. Adapted with permission from Ref. [58]. Copyright 2021, American Chemical Society.

Thus, silica plays a crucial role in serving as a slightly acidic medium, desiccant, and grinding agent, while acetic acid contributes to increasing the acidity in the reaction environment. Following analogous Biginelli‐type procedures in solution, this mechanochemical reaction also occurs in the presence of Cs_2_CO_3_, but no further information on this was given. This optimized mechanochemical procedure allowed the preparation of a library of widely decorated sulfonimidamides via a one‐pot multi‐component process with high atom‐economy efficiency (Scheme 6).

**Figure 7:**
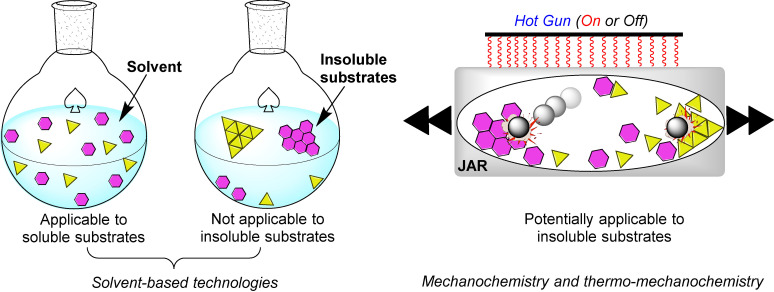
Tackling solubility issues in organic synthesis: conventional solution procedures and mechanochemical protocols. Adapted with permission from Ref. [66a]. Copyright 2021, American Chemical Society.

As we have seen so far, the poor solubility of some reagents in the most common organic solvents adversely affects their reactivity. On the other hand, the high diffusion efficiency of the reactants in solution‐based reactions is a crucial prerequisite for promoting complete chemical transformations under homogeneous conditions (Figure 7). In this regard, the choice of solvent, where available, is critical to the success of the process, affecting selectivity, reaction rate, and chemical equilibrium. That said, the problem becomes relevant if no solvents are available to dissolve the reagents (Figure 7).

Polyaromatic compounds are included in many cutting‐edge organic functional materials, such as luminescent materials^[60]^ and organic semiconductors^[61]^ that are investigated for a wide range of applications in organic electronics, including solar cells, light‐emitting diodes, and batteries. However, their low solubility often complicates synthetic processes involving polyaromatic compounds. Many polyaromatics are poorly soluble substrates in common organic solvents, and, as a result, they exhibit a significant decrease in the reaction rate. Although some reactions can be developed under slurry conditions, even running at high temperatures, the expected products are sometimes not recovered or only with low yields.

**Scheme 7:**
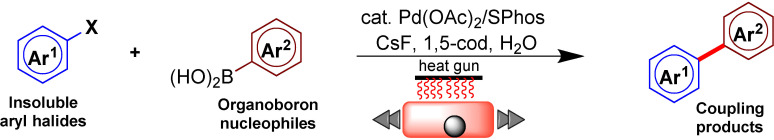
Cross‐coupling reactions with insoluble aryl halides. Adapted with permission from Ref. [66a]. Copyright 2021, American Chemical Society.

The well‐known Suzuki‐Miyaura cross‐coupling reaction^[62]^ between aryl halides and aryl boron nucleophiles represents the most efficient and straightforward route to access π‐conjugated molecules.^[63]^ However, the conventional solution‐based Suzuki–Miyaura reaction^[64]^ is of limited effectiveness when poorly soluble polycyclic aryl halides are used as building blocks to assemble new carbon‐carbon bonds (Scheme 7).^[65]^

**Figure 8:**
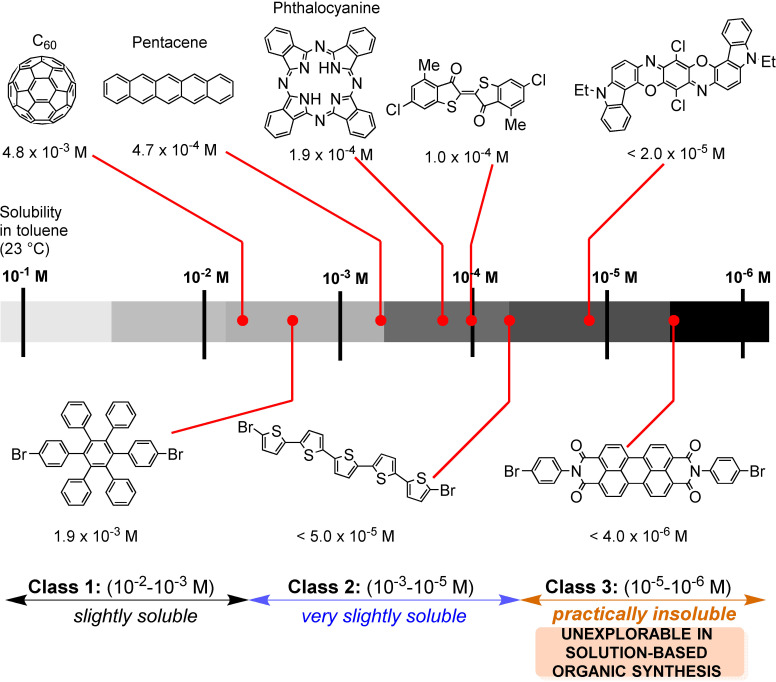
Classification of aryl halides based on their solubility for systematically evaluating their solid‐state cross‐coupling reactions using mechanochemistry. Adapted with permission from Ref. [66a]. Copyright 2021, American Chemical Society.

**Scheme 8:**
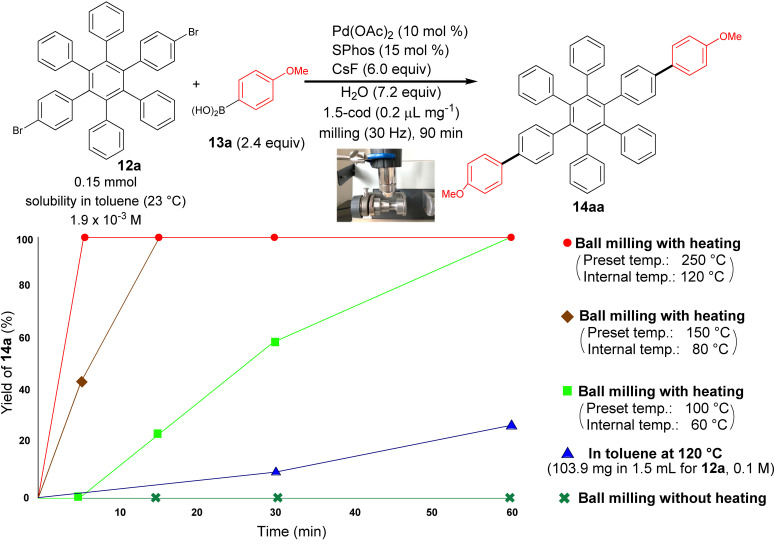
Application of the high‐temperature ball‐milling method to solid‐state Suzuki–Miyaura cross‐coupling reactions. Adapted with permission from Ref. [66a]. Copyright 2021, American Chemical Society.

Several authors deeply investigated the Suzuki–Miyaura reaction by mechanochemistry.^[64]^ Later on, in a seminal paper, Ito and co‐workers^[66a]^ provided a deeper understanding of the reactivity of the system under mechanochemical conditions, subdividing aryl halides into three solubility classes by referring to US Pharmacopoeia criteria^[67]^ to systematically evaluate the relationship between the reactivity and the insolubility (Figure 8). In a second step, they developed an efficient mechanochemical strategy for the solid‐state cross‐coupling of insoluble aryl halides **12 a** using a significantly performing catalytic system consisting of Pd(OAc)_2_/SPhos/1,5‐cod (Figure 8 and Scheme 8). Herein, 1,5‐cyclooctadiene (1,5‐cod) works as dispersant and stabilizer to promote C−C coupling under solid‐state reaction conditions.^[66a]^

The authors have studied this mechanochemical process operating at a higher temperature (120 °C) to speed up the solid‐state cross‐coupling. They employed a commercially available, temperature‐controllable heat gun placed above the running jars during the grinding process. The heat gun set at a temperature of 250 °C ensures a temperature of 120 °C inside the jar, as confirmed by thermography upon opening the milling jar.

The reaction of **12 a** in the presence of the Pd(OAc)_2_/DavePhos/1,5‐cod catalytic system was fast and went up to completion in a few minutes, almost affording the desired product **14 aa** in high yield (96 %; Scheme 8).

**Scheme 9:**
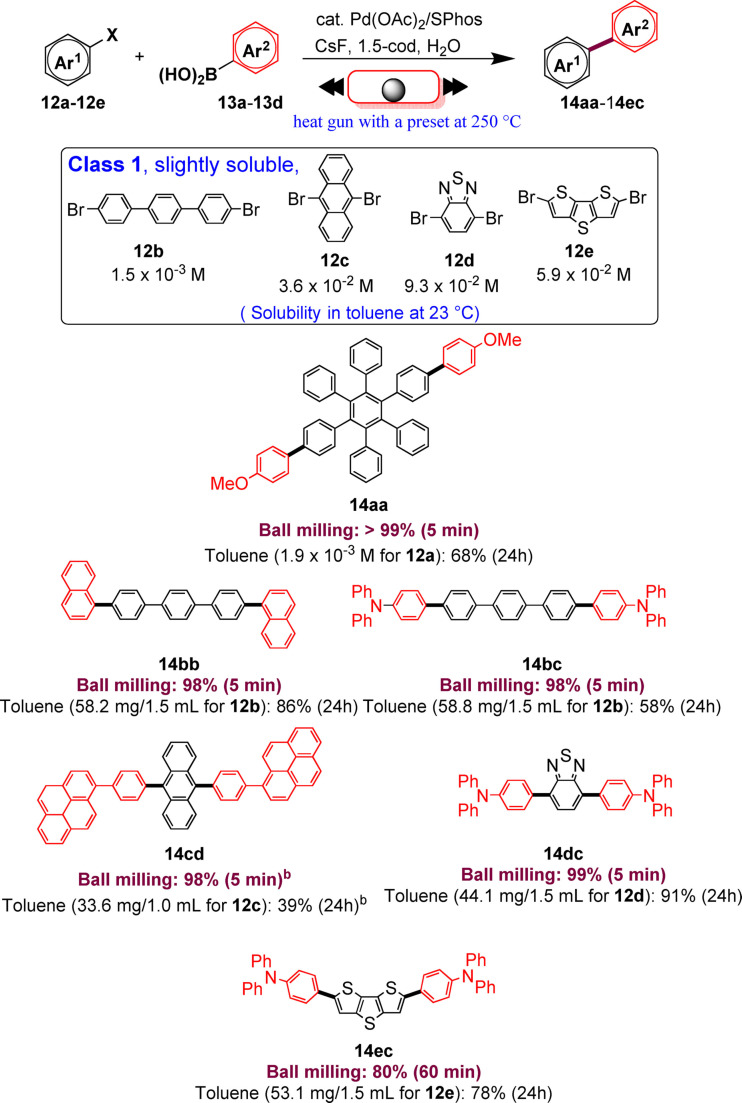
Solid‐state cross‐coupling reactions using high‐temperature ball milling.^[a]^ [a] Unless otherwise noted, the following reaction conditions were used for the solid‐state cross‐coupling reactions: **12** (0.15 mmol), **13** (0.36 mmol), Pd(OAc)_2_ (0.015 mmol), SPhos (0.023 mmol), CsF (0.9 mmol), H_2_O (1.08 mmol), and 1,5‐cod (0.20 μL mg^−1^) in a stainless‐steel ball‐milling jar (1.5 mL) with a stainless‐steel ball (5 mm); ball milling (30 Hz) carried out while using a heat gun with a preset temperature of 250 °C. Conditions for the solution‐based cross‐coupling reactions: **12** (0.15 mmol), **13** (0.36 mmol), Pd(OAc)_2_ (0.015 mmol), SPhos (0.023 mmol), CsF (0.9 mmol), H_2_O (1.08 mmol), and toluene (1.5 mL) at 120 °C for 24 h. [b] Reactions were carried out on a 0.1 mmol scale. Adapted with permission from Ref. [66a]. Copyright 2021, American Chemical Society.

In this study, aryl halides **12 a**–**12 e** classified as “slightly soluble” (class 1) afforded the desired products **14 aa**–**14 ec** in quantitative yields after 5 min of ball milling in most cases (Scheme 9). Conversely, solution‐based conditions provided lower yields, even at prolonged reaction times (24 h, Scheme 9).

**Scheme 10:**
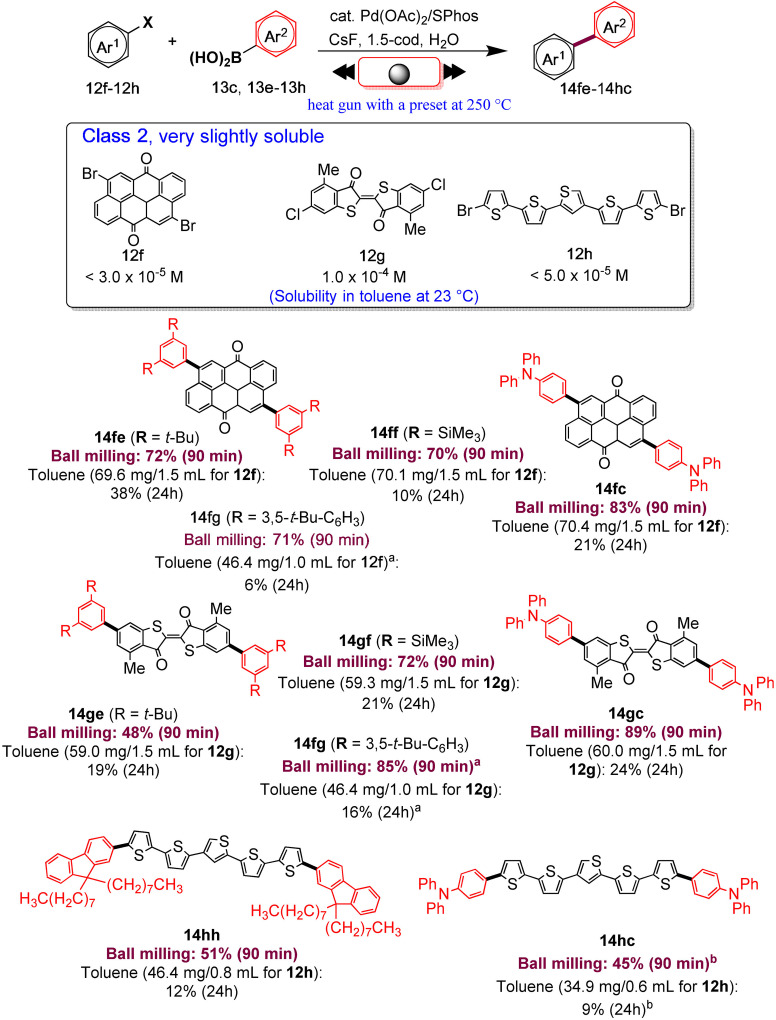
Solid‐state cross‐coupling reactions of poorly soluble aryl halides that are barely suitable for solution‐based coupling reactions. Unless otherwise noted, the following reaction conditions were used for the solid‐state cross‐coupling reactions: **12** (0.15 mmol), **13** (0.36 mmol), Pd(OAc)_2_ (0.015 mmol), SPhos (0.023 mmol), CsF (0.9 mmol), H_2_O (1.08 mmol), and 1,5‐cod (0.20 μL mg^−1^) in a stainless‐steel ball‐milling jar (1.5 mL) with a stainless‐steel ball (5 mm); ball milling (30 Hz) carried out using a heat gun with a preset temperature of 250 °C. Conditions for the solution‐based cross‐coupling reactions: **12** (0.15 mmol), **13** (0.36 mmol), Pd(OAc)_2_ (0.015 mmol), SPhos (0.023 mmol), CsF (0.9 mmol), and H_2_O (1.08 mmol) in toluene (1.5 mL) at 120 °C for 24 h. [a] The reactions were carried out on a 0.1 mmol scale. [b] The reactions were carried out on a 0.06 mmol scale. Adapted with permission from Ref. [66a]. Copyright 2021, American Chemical Society.

The mechanochemical solid‐state coupling of aryl halides, classified as “very slightly soluble” (class 2), was also found to react smoothly and efficiently to give the corresponding products in high yield (45–89 %, Scheme 10).

In contrast, this substrate class afforded the expected products with unsatisfactory yields (9–38 %) under solution‐based conditions (Scheme 10).

**Scheme 11:**
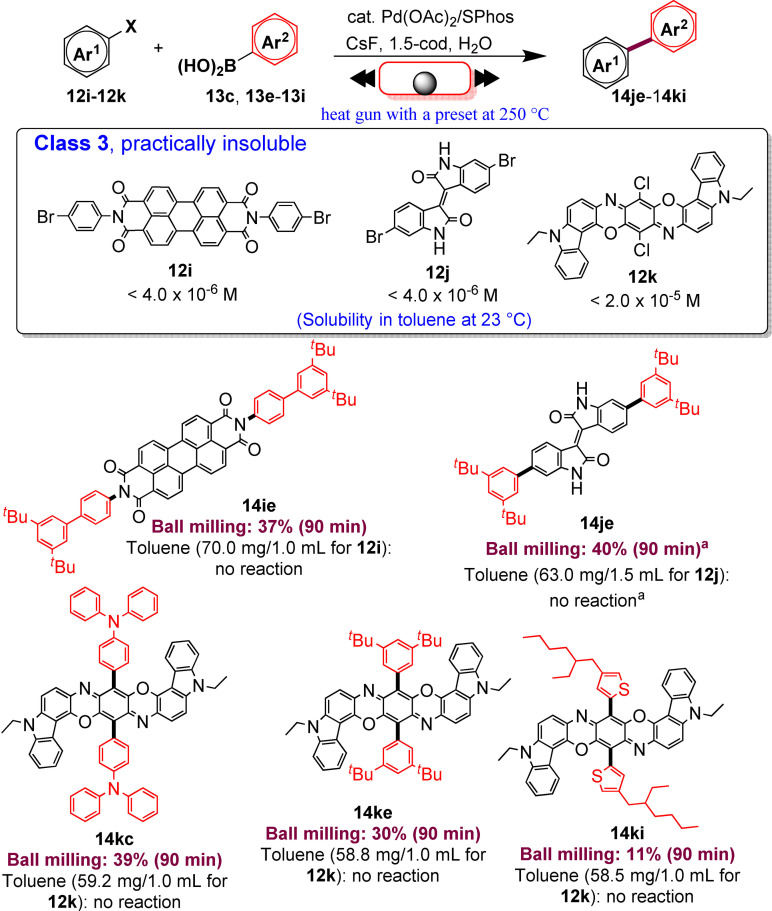
Solid‐state cross‐coupling reactions of poorly soluble aromatic compounds that are not suitable for solution‐based coupling conditions. Unless otherwise noted, the following mechanochemical conditions were used for the solid‐state cross‐coupling reactions: **12** (0.10 mmol), **13** (0.24 mmol), Pd(OAc)_2_ (0.010 mmol), SPhos (0.015 mmol), CsF (0.6 mmol), H_2_O (0.54 mmol), and 1,5‐cod (0.20 μL mg^−1^) in a stainless‐steel ball milling jar (1.5 mL) with a stainless‐steel ball (5 mm); ball milling (30 Hz) was carried out using a heat gun with a preset temperature of 250 °C. Conditions for the solution‐based cross‐coupling reactions: **12** (0.10 mmol), **13** (0.24 mmol), Pd(OAc)_2_ (0.010 mmol), SPhos (0.015 mmol), CsF (0.6 mmol), H_2_O(0.54 mmol), and toluene (1.0 mL) at 120 °C for 24 h. [a] The reactions were carried out on a 0.15 mmol scale. Adapted with permission from Ref. [66a]. Copyright 2021, American Chemical Society.

In addition, the reaction rates of those aryl halides in the solid‐state (90 min) were much higher than those in the solution (24 h, Scheme 10). It is well known from the literature that in solution‐based organic synthesis, the cross‐coupling products of chlorides, classified as “practically insoluble” (class 3), were never detected, even in traces (Scheme 11).

Ito and co‐workers^[66a]^ overcome these limitations, developing a remarkable mechanochemical solid‐state organic synthesis at 120 °C, which provides new opportunities to expand the molecular diversity of polyaromatic compounds derived from insoluble starting materials (Scheme 11). Furthermore, a commercially available, temperature‐controllable heat gun, placed directly above the ball‐milling jar, has made these achievements possible and led to new luminescent organic material with solid red emission.

The formation of new intramolecular aryl–aryl bonds is a crucial transformation for assembling nanographenes, graphene nanoribbons (GNR), and polycyclic aromatic hydrocarbons (PAHs), a class of compounds with important applications in organic electronics.^[68]^

To date, there are two widely used synthetic approaches leading to this cyclodehydrogenation: a thermally activated^[69]^ on‐surface procedure commonly carried out on metal substrates (e. g., Cu) inside an electron microscope at temperatures around 177 °C, and the solution‐mediated Scholl reaction,^[70]^ catalyzed by one‐electron oxidants (e. g., FeCl_3_ or MoCl_5_) performed at room temperature or slightly elevated temperatures. These two methodologies are pivotal in their respective fields in preparing a wide‐ranging library of nanographenes and GNRs.^[64]^ However, the intrinsically low solubility of reagents and products makes these substrates challenging to synthesize. The addition of solubilizing group to the scaffold enhances its solubility in organic solvents, but this involves additional steps, generating enormous amounts of waste.

**Figure 9:**
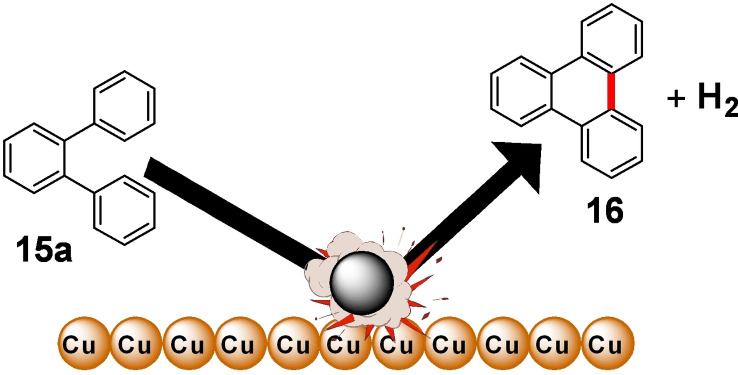
Mechanochemical cyclodehydrogenation reaction with elemental copper to polyaromatics. Adapted with permission from Ref. [72]. Copyright 2020, American Chemical Society.

Mechanochemistry provides a practical solution to overcome these limitations enabling the oxidative Scholl reaction without solvents inside a ball mill.^[71]^ For example, Borchardt and co‐workers^[72]^ have recently demonstrated that the solvent‐free environment of the ball mill can be used to conduct the cyclodehydrogenation of nanographenes, avoiding the chlorination of the products and the use of dangerous chlorinating agents that release significant amounts of corrosive HCl (Figure 9).

Inspired by the surface‐supported procedure on a copper (111) plane, they adapted this protocol to a room‐temperature procedure inside the ball mill, exploring the influence of different milling parameters on *o*‐terphenyl **15 a** as a reference substrate (Table 2).

**Table 2:** Reaction conditions and yields of triphenylene mechanochemical syntheses.^[a]^ Adapted with permission from Ref. [72]. Copyright 2020, American Chemical Society.

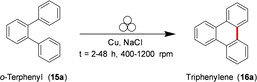				
Entry	Sample	Milling time [h]	Milling speed [rpm]	Yield^[b]^ [%]
1	P7‐12‐400	12	400	<1
2	P7‐12‐800	12	800	20
3	P7‐24‐800	24	800	25
4	P7‐48‐800	48	800	>99
5	Emax‐12‐1200^[c]^	12	1200	10
6	Emax‐12‐1500^[c]^	12	1500	26
7	Emax‐48‐1200^[c]^	48	1200	66
8	P7‐12‐800‐WC^[d]^	12	800	37
9	Emax‐12‐1200‐WC^[c,d]^	12	1200	>99
10	P7‐12‐800‐Fe^[e]^	12	800	<1
11	P7‐12‐800‐Fe_2_O_3_^[e]^	12	800	<1
12	P7‐12‐800‐Ni^[e]^	12	800	10
13	P7‐12‐800‐Co^[e]^	12	800	<1
14	P7‐12‐800‐Ar^[f]^	2	800	<1
15	P7‐12‐800‐Ar^[f]^	12	800	10
16	P7‐48‐800‐HPB^[g]^	48	800	>99

[a] Reaction conditions if not stated otherwise: 0.1 g of *o*‐terphenyl, 1.9 g of copper powder, 2.5 g of NaCl (bulking material), 22×10 mm balls ZrO_2_ in a 45 mL ZrO_2_ vessel. Sample code: mill+milling time+milling speed, for example, P7‐12‐400: Pulverisette 7, 12 h at 400 rpm. [b] Yield after purification. [c] Experiments in the Emax have been conducted with 16×10 mm balls. [d] Tungsten carbide milling balls and vessel; 8 g of NaCl as bulking material. [e] Fe, Fe_2_O_3_, Ni, and Co powder, respectively, were used instead of Cu powder. [f] Conducted under an argon atmosphere. [g] Hexaphenylbenzene was used as the starting material.

The authors initially investigated the mechanochemical cyclodehydrogenation process, using a planetary mill (Pulverisette 7, P7) to determine the minimum amount of copper needed to achieve a good conversion (Table 2). The mechanochemical reaction carried out in a planetary mill (Pulverisette 7, P7) requires long reaction times (up to 48 h) to ensure complete conversion of the *o*‐terphenyl toward triphenyl **16 a** (Table 2, entry 4).

**Scheme 12:**
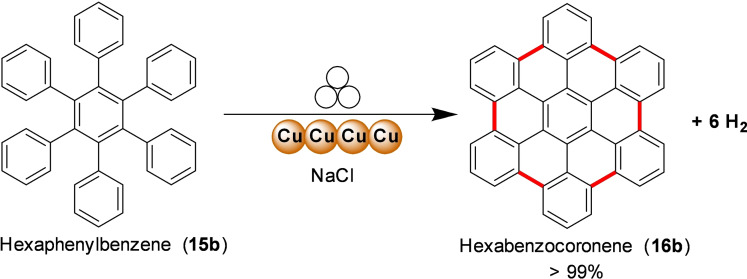
Mechanochemical planarization reaction of hexaphenylbenzeneand elemental copper to hexabenzocoronene. Adapted with permission from Ref. [72]. Copyright 2020, American Chemical Society.

Reaction times can be cut down if sufficient energy input is provided during impacts (Table 2, entries 1–4). However, the higher temperatures attained inside the jar, especially with tungsten carbide balls (100 °C), if not appropriately controlled, led to the degradation of the freshly formed product. The problem of thermal degradation has been overcome by using tungsten carbide milling balls and a ball mill (Emax, Table 2, entries 5–9) that allows for partial cooling of the jars. Combining these two advances led to the desired triphenylene in a quantitative yield in only 12 h of reaction without any side reaction like dimerization (Table 2, entry 9). The developed protocol has been successfully extended to hexaphenylbenzene, proving the ability to planarize bigger nanographene derivatives (Scheme 12).

**Scheme 13:**
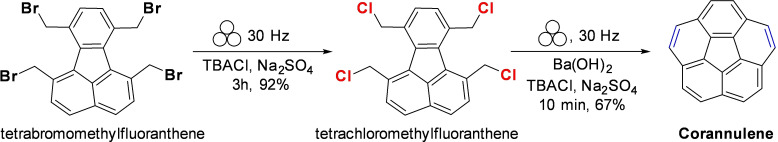
Mechanochemical synthesis of corannulene from planar polyarenes. Adapted with permission from Ref. [73]. Copyright 2021, Springer Nature.

Mechanochemistry also allowed obtaining fused ring systems from planar polyarenes (Scheme 13).^[73]^ Corannulene was obtained in 67 % yield from tetrabromomethylfluoranthene precursor under ambient conditions, in only 10 min, with no need for dry conditions, heating, or organic solvents. Tetrachloromethylfluoranthene, in turn, has been prepared in high yield (92 %), subjecting tetrabromomethylfluoranthene to a mechanochemical halide‐exchange reaction with the help of tetrabutylammonium chloride (TBACl). This two‐step mechanochemical procedure is highly competitive with traditional solution^[74]^ (14 %) and gas‐phase^[75]^ synthetic pathways (18 %) in terms of yield and reaction time.

Polyaromatic molecules such as corannulene can be viewed as nano‐graphenes prepared through a bottom‐up process. In the case of graphene, mechanochemistry can also be employed to form graphene by the deconstruction of graphite. In fact, the process of discovering graphene, where layers were peeled off with the help of scotch tape, is of mechanochemical character.^[76]^ As that method is unsuitable for large‐scale graphene production, various novel methodologies have been developed, with prominent examples being ultrasonication or shear‐mixing of graphite dispersions in solvents. This topic has been extensively reviewed, and we refer the interested reader to a recent, very comprehensive Review.^[77]^ It should be noted that such methodology will lead to mixtures of particles with different amounts of stacked graphene sheets. A crucial issue of exfoliating graphite into graphene is avoiding re‐stacking graphene sheets. Because of this, milling of graphite is performed in the presence of agents that can prevent such restacking. A wide range of different agents can be employed; for example, graphite can be milled in the presence of sugars,^[78]^ organic solvents,^[79]^ salts,^[80]^ small aromatic molecules^[81]^ dry‐ice,^[82]^ or melamine or other triazine derivatives.^[83]^ Another option is to mill graphite in water in the presence of surface‐active agents such as proteins,^[84]^ resulting in aqueous graphene dispersions. Some of these materials have been investigated for applications involving charge storage.^[85]^ In another example, employing graphite milled with proteins, an aqueous dispersion was obtained that could be processed into thin films that displayed a voltage when exposed to a temperature gradient.^[85b]^ Such materials can potentially be used for thermoelectric applications where waste heat can be converted into electricity. In another example related to organic electronics, lignin was milled with graphite, resulting in a carbon paste that could be processed into films capable of charge storage.^[86]^ Lignin contains quinone groups that can store charge by redox processes. However, it is electronically insulating, and in bulk, to contact molecules, lignin must be mixed with a conductor that can transport charges to the redox‐active sites. This requires the mixing of lignin and an electronic conductor (graphite). When processed by mechanochemistry, materials were obtained where graphite provided charge transport to lignin redox sites, showing promising charge storage characteristics. These results can inspire further development of materials for charge storage applications.^[87]^ Mechanochemical methodology has also been employed to prepare graphene‐related materials for biomedical applications. For example, mechanochemical processes were used to prepare hybrid materials constituted by graphene oxide (GO) and superparamagnetic iron oxides nanoparticles coated with oleic acids.^[88]^ In a different approach, the solvent‐free mechanochemical copper‐catalyzed click reaction between the hydrophilic maleimide‐PEG‐N_3_ and hydrophobic nano‐GO bearing a propargyl group led to a nano‐GO‐PEG hybrid material, further functionalized with antibodies targeting cancer cells.^[89]^

Acid–base, as well as nucleophilic substitution reactions, represent a crucial skill tool for synthetic organic chemists. When performed by mechanochemistry, the lack of solvation (absence of solvent) allows bases and nucleophiles to be stronger than in solution, enabling milder reaction conditions. Although these reactions are straightforward in generating new bonds, they require reagents (alkyl lithium, amides, and hydrides) and reaction conditions^[90]^ that raise significant environmental and public health concerns.

In a chemical process, the solvent is a hazardous item with a high environmental impact that suppresses reactivity because of the stabilization offered by the solvation of the reagents. Mechanochemistry has at least three crucial advantages over solution‐based processes: it significantly reduces the negative impacts of solvents on the environment, improves the reagent's reactivity substantially, and cuts down the additional energy demand due to solvation.^[91]^ Furthermore, mechanochemistry allows bases and nucleophiles to be stronger species under mechanochemical conditions than in solution, enabling milder and safer reaction conditions.

**Scheme 14:**
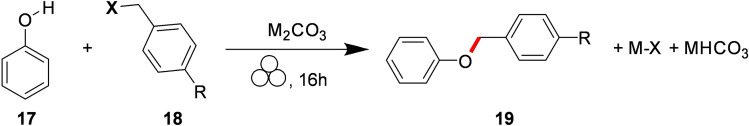
Typical reaction scheme for the nucleophilic substitution of phenol towards various benzyl halides. Adapted with permission from Ref. [92]. Copyright 2020, Royal Society of Chemistry.

To better understand and rationalize the lack of solvent shells in mechanochemical processes, Mack and co‐workers^[92]^ have investigated the effect of ion pairing and the use of weak bases for driving forward nucleophilic substitution reactions. The nucleophilic substitution of phenol towards various benzyl halides has been used as a model to study the interaction between the phenoxide and the different alkali metals, as shown in Scheme 14.

Two crucial interactions drive this solventless reaction: (a) the metal–oxygen interaction (i. e., “M−O” interaction) of the carbonate, (b) the metal–halogen interaction (i. e., the “M−X” interaction), and both of them focus on the metal cation of the base (i. e., “M”). The synthesis of benzyl ether can proceed, giving the desired product together with a metal‐halogen salt only if “M−X” ion pair is strong and the “M−O” ion pair is weak as under these conditions, the supposed intermediate is expected to form. Under mechanochemical conditions, a definite trend for both conversion and yield appears to increase as the alkali metal and halogen become larger and more polarizable (Table 3). The alkali metals have a significant effect on the efficiency of the reaction, and the most reactive are those with little overlap between the ions according to either the Pearson's hard soft acid base theory^[93]^ and Jones–Dole viscosity B coefficients^[94]^ (Table 3).

**Table 3:** Percent conversion and yield of the phenol and carbonate reaction. Adapted with permission from Ref. [92]. Copyright 2020, Royal Society of Chemistry.

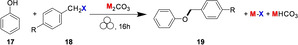					
Entry	R	X	M	Conv. [%]	Yield^[a]^ [%]
1	Cl	Cl	Li	3	3
2	Cl	Cl	Na	28	10
3	Cl	Cl	K	47	44
4	Cl	Cl	Rb	62	54
5	Cl	Cl	Cs	75	72
6	Br	Br	Li	<2	<2
7	Br	Br	Na	43	38
8	Br	Br	K	69	45
9	Br	Br	Rb	79	60
10	Br	Br	Cs	87	74
11	Br	I	Li	<2	<2
12	Br	I	Na	55	49
13	Br	I	K	77	68
14	Br	I	Rb	83	78
15	Br	I	Cs	94	89

[a] Isolated yields.

This trend was also observed for the reactivity of carbonates with the benzyl alcohol, where, unlike what can be observed in solution, the carbonates are strong enough bases to promote alkylation reactions (Table 4). To increase the reactivity of carbonates, 2 equiv. of Cs_2_CO_3_ need to promote the complete conversion of benzyl alcohol (94 %, Table 4, entry 10). In contrast to what has been observed in solvent‐based reactions, mechanochemistry allows replacing hydroxides with more environmentally benign carbonates efficiently.

**Table 4:** Comparison of 1 mmol and 2 mmol of carbonate base used in a substitution reaction. Adapted with permission from Ref. [92]. Copyright 2020, Royal Society of Chemistry.

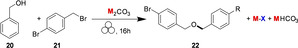			
Entry	M	Equiv. of base	Yield^[a]^ [%]
1	Li	1	5
2	Na	1	8
3	K	1	13
4	Rb	1	34
5	Cs	1	54
6	Li	2	5
7	Na	2	8
8	K	2	55
9	Rb	2	76
10	Cs	2	94

[a] Isolated yields.

**Scheme 15:**
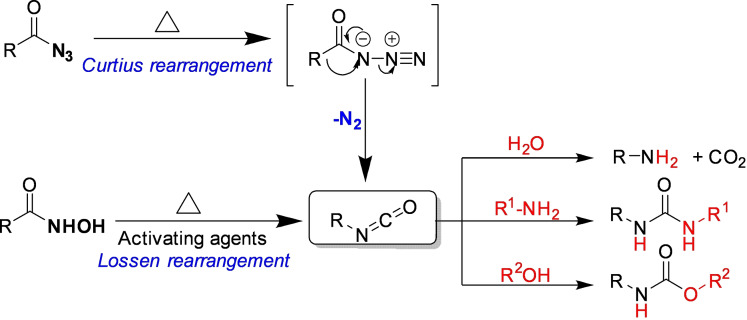
Curtius rearrangement. Adapted with permission from Ref. [97]. Copyright 1988, American Chemical Society.

Organic azides are versatile organic compounds with the potential to be used for a wide range of reactions.^[95]^ In many chemical processes, this class of compounds allows the insertion of a nitrogen atom into the molecular architecture.^[96]^ Generally, the external energy inputs trigger the release of nitrogen gas from the organic azide functionality, which is the driving force behind the whole process (Scheme 15).^[97]^

However, many acyl azides are extremely thermally unstable intermediates, and the control of the reaction remains problematic in the design of chemical processes involving these organic molecules.^[98]^ For example, carbamoyl azides or benzoyl azides, when heated around 50 °C, quickly undergo molecular rearrangement (Curtius rearrangement), forming isocyanates, which significantly limit its range of applications (Scheme 15).^[99]^

In the literature, mechanochemical azide transformations have been previously described only with relatively stable sulfonyl and alkyl azides. Užarević and co‐workers^[100]^ recently observed that acyl azide does not undergo Curtius rearrangement into isocyanate under high‐speed ball milling conditions. Contrary to what one might initially expect, this suggests that the energy barrier for Curtius rearrangements is too high to be overcome in a conventional ball mill working at room temperature. These types of rearrangements can be achieved by mechanochemical activation of hydroxamic acids with CDI, (Lossen rearrangement), as recently proven by Colacino and co‐workers.[^5f^, ^101^] Užarević *et al*.^[100]^ designed and performed a series of experiments in a jar heated to 80 °C for 60 min that allowed them to overcome the energy barrier of the Curtius reaction, enabling the formation of the corresponding ureas.

In solution, indeed, the low ceiling temperature has significantly limited the selective acyl nitrene transfer reactions. For example, benzoyl azides or carbamoyl azides quickly rearrange to isocyanate at temperatures slightly above the ambient (around 50 °C), significantly off‐putting any possible metal‐catalyzed nitrene transfer C−H amidation process.

**Scheme 16:**
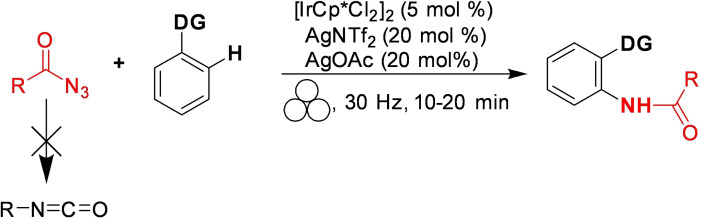
Solvent‐free mechanochemical C−H amidation. Adapted with permission from Ref. [102]. Copyright 2021, American Chemical Society.

Conversely, a mechanochemical system could offer better control and a broader synthetic spectrum for these transformations than analogous thermal reactions. Kim and co‐workers^[102]^ showed that thermally unstable acyl azides behaved differently in high‐speed ball milling. In their study, they developed a selective Ir^III^‐catalyzed mechanochemical C−H amidation, avoiding the sudden decomposition of acyl azides to isocyanate (Scheme 16).

The energy delivered during impacts in the jar is strongly correlated to the density of the materials (Teflon, ZrO_2_, SS, WC) of the container and balls (Table 5, entries 1–4). Proton nuclear magnetic resonance (^1^H NMR) and FTIR spectroscopy pointed out that the low‐energy condition, Teflon jar (2.2 g cm^−3^), high‐density zirconia (ZrO_2_, 5.7 g cm^−3^) and stainless steel ball (SS, 7.9 g cm^−3^) resulted in no acyl azide rearrangement of **23 a** to isocyanate **24 a** (Table 5, entries 1–5). At the same time, heavy tungsten carbide (WC, 15.6 g cm^−3^) caused isocyanate formation (21 %, Table 5, entry 4).

**Table 5:** Stability test of benzoyl azides under ball milling conditions.^[a]^ Adapted with permission from Ref. [102]. Copyright 2021, American Chemical Society.

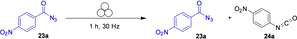				
Entry	Jar (10 mL)	Ball diameter [mm]	Yield **23 a** [%]	Yield **24 a** ^[b]^ [%]
1	Teflon	SS (7 mm×2)	98	0
2	ZrO_2_	ZrO_2_ (8 mm×2)	92	7
3	SS	SS (7 mm×2)	91	5
4	WC	WC (7 mm×2)	71	21

[a] Reaction conditions: **1 a** (0.8 mmol, 154 mg) milled in each container and balls; the crude mixture was collected by chloroform (2 mL×3) and filtered directly using Celite pads. [b] Yield based on ^1^H NMR spectroscopy of the crude reaction mixture using CH_2_Br_2_ as the internal standard.

**Scheme 17:**
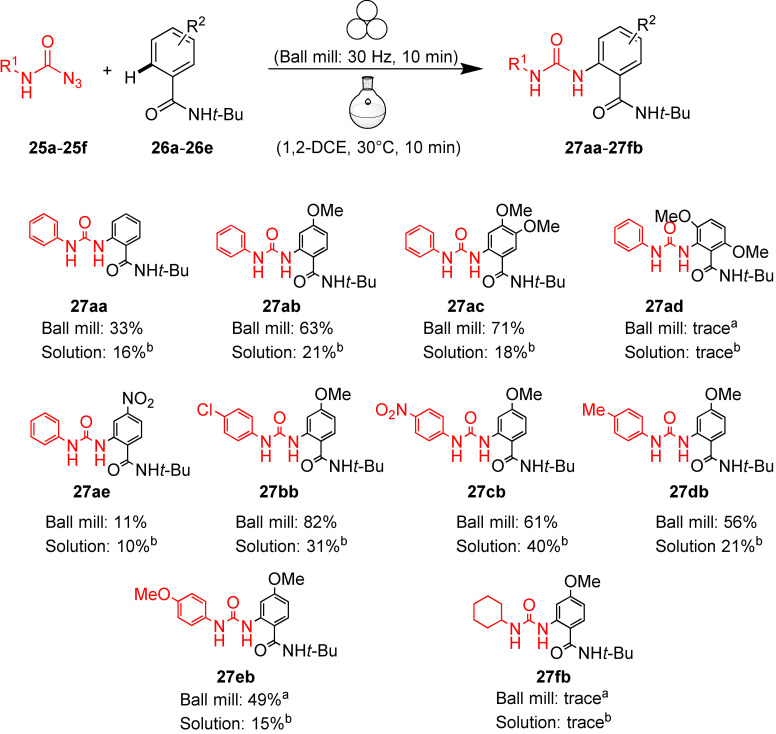
Ir‐Catalyzed sp^2^ C−H amidation with various carbamoyl azides. [a] Conditions: **25** (0.15 mmol), **26** (0.1 mmol), [IrCp*Cl_2_]_2_ (10 mol %, 8 mg), AgNTf_2_ (20 mol %, 8 mg), and AgOAc (40 mol %, 7 mg) were ball milled in a mixer mill at 30 Hz, using a 10 mL Teflon milling jar with one stainless‐steel ball of 10 mm diameter. [b] Conditions: **25** (0.15 mmol), **26** (0.1 mmol), [IrCp*Cl_2_]_2_ (10 mol %, 8 mg), AgNTf_2_ (20 mol %, 8 mg), and AgOAc (40 mol %, 7 mg) in 1,2‐DCE (dichloroethane, 0.5 mL) for 10 min at 30 °C. Adapted with permission from Ref. [102]. Copyright 2021, American Chemical Society.

The reaction conditions used by Chang and co‐workers in their Cp*Ir^III^‐catalyzed C−H amidation in solution were then adapted for the procedure in a high‐speed vibratory ball mill.^[103]^ The authors got an even more impressive result when this mechanochemical procedure was extended to prepare unsymmetrical ureas through Ir^III^ catalyzed C−H amidation with carbamoyl azides (Scheme 17). In solution, monosubstituted carbamoyl azides exhibited low reactivity (Scheme 17). An increase in temperature for thermal activation was not applicable as it triggered the Curtius rearrangement of carbamoyl azides to isocyanates. The developed ball milling procedure overcomes the restrictions occurring under thermal solution conditions by allowing carbamoyl azides utilization.

### 2.2 Mechanochemical formation of higher‐ordered peptide structures

**Scheme 18:**
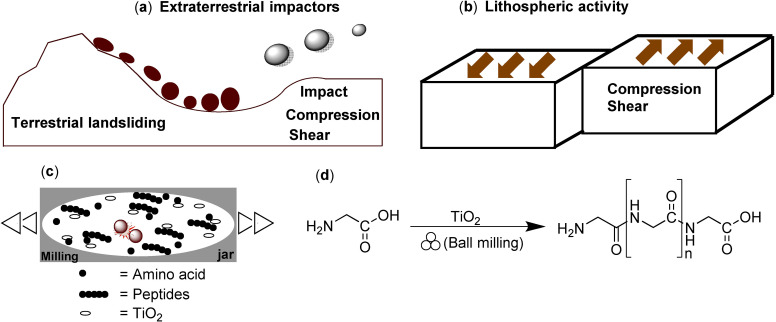
The mechanical energy in prebiotic impact scenarios could have been provided by impact, compression, and shear forces. (a) Extraterrestrial and terrestrial collisions. (b) Plate tectonic movement. (c) Ball mill containing reactants: amino acids, peptides, and TiO_2_. (d) Solvent‐free mechanochemical peptide bond formation by ball milling. Adapted with permission from Ref. [109]. Copyright 2021, Wiley‐VCH.

Although it is generally accepted that amino acids were present on the prebiotic Earth,^[104]^ either by endogenous formation or by delivery in meteorites,^[105]^ the mechanochemical formation of higher‐ordered peptide structures from amino acids mainly had only been hypothesized (Scheme 18).^[106]^ Prebiotically plausible reaction pathways^[107]^ for amide bond formation from inactivated amino acids are still doubtful as most oligomerization reactions rely on strong assumptions that they are thermodynamically disfavored processes in solution (Scheme 18).^[108]^

**Scheme 19:**
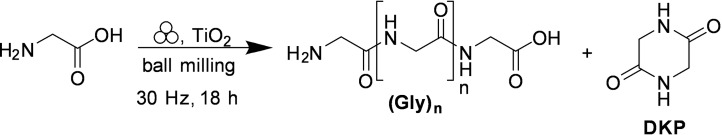
Mechanical oligomerization of pristine glycine (H‐Gly‐OH) as a model amino acid. Adapted with permission from Ref. [109]. Copyright 2021, Wiley‐VCH.

Hernández and co‐workers^[109]^ showed that TiO_2_, a prebiotically plausible mineral, combined with mechanochemical activation, promoted oligomerization of glycine at ambient temperature and in the absence of water (Scheme 19). The milling temperature has proven critical for the oligomerization process as raising the reaction temperature increases both the degree of oligomerization and the concomitant formation of undesired cyclic glycine dimer (DKP, Scheme 19 and Table 6). Ion‐pair high‐performance liquid chromatography (IP‐HPLC) analysis revealed that high temperatures (70–130 °C) heavily influence the selectivity of the mechanochemical oligomerization process. At 130 °C, DKP was the main component of the product mixture (Scheme 19).

**Table 6:** Effect of the milling temperature on the mechanochemical oligomerization of Gly with TiO_2_. Adapted with permission from Ref. [109]. Copyright 2021, Wiley‐VCH.

Milling temperature [°C]	Longest detected oligomer^[a]^	Yield^[b]^ [%]
RT	Gly_6_	6.7
40	Gly_6_	6.7
70	Gly_7_^[c]^	10.1
100	Gly_8_	10.2
130	Gly_10_^[d]^	8.5

[a] Based‐on IP‐HPLC analysis using a UV/Vis detector set to record the absorbance at 195 nm. [b] Combined yield of all linear oligomers of glycine Gly>2. [c] The presence of Gly_8_ was detected by the UPLC‐MS method. [d] The presence of Gly_11_ was detected by UPLC‐MS.

The mechanochemical oligomerization of glycine (Table 6) allowed for a maximum calculated total yield (UPLC‐MS) of glycine oligomers (Gly>2) of 10.2 % at 100 °C and a repetitive peptide sequence of glycines, which is 14 residues long (Gly_14_).

When DKP or Gly_2–3_ were used as starting materials, the mechanochemical peptide bond formation occurred as a dynamic and reversible process with concomitant forming and breaking of amide bonds.

The interest in peptides synthesis is still highly topical today,^[110]^ and it is not completely limited to how life originated in the early Earth.^[111]^ Peptides found numerous applications in our everyday lives, playing a pivotal role in different branches of physics, chemistry, material engineering, and biological sciences.^[112]^

Building up a long peptide sequence is still a highly challenging and laborious task since it requires assembling many amino acid units. Indeed, coupling and deprotection reactions become less efficient as the peptide chain length becomes longer, resulting in amino acid deletions and uncompleted sequences.^[113]^ The risk of epimerization represents an even more severe limitation in the amide coupling of a C‐term activated peptide fragment with a free N‐term residue, causing the formation of highly unwanted diastereomers.^[114]^ However, examples with little or no evidence of epimerization are rare, and only a few results are reported in the literature.

In response to these issues, Métro and co‐workers^[115]^ assessed the capacity of ball mills to reduce or eliminate the propensity for epimerization during the peptide coupling, comparing these results to those obtained with classical approaches in solution (Table 7). In addition, mechanosynthesis was developed using two different target amino acids: phenyl glycine (H‐Phg‐OH), known for its increased tendency for racemization, and isoleucine (H‐Ile‐OH), which has a significant steric hindrance that hampers the amide bond formation (Table 7). The amount of DMF (*η*=0.45 μL mg^−1^) involved as a liquid additive during the synthesis is insufficient to dissolve all reagents. Still, it is crucial to promote the formation of the corresponding tripeptide Z‐Ala‐(L)‐Phg‐Ile‐OMe, which was recovered with excellent purity (HPLC) and in a very high yield (93 %).

**Table 7:** Comparative study between vibrating ball milling and solution synthesis of Z‐Ala‐Phg‐Ile‐OMe.^[a,b]^ Adapted with permission from Ref. [115a]. Copyright 2021, American Chemical Society.

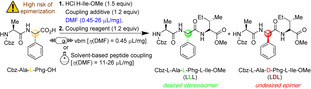						
Entry	Reagents	*T* [°C]	*t* [min]	Yield [%]	Purity^[b]^ [%]	LDL^[b]^ [%]
1	EDC⋅HCl/ Oxyma	33 (33)	10 (30)	93 (88)	>99 (32)	<1 (9)
2	EDC⋅HCl/HOBt⋅H_2_O	34 (34)	10 (30)	90 (90)	70 (48)	25 (35)
3	EDC⋅HCl/HOAt	34 (34)	10 (30)	88 (90)	95 (59)	<1 (26)
4	DIC/HOAt	30 (31)	10 (40)	n.d. (n.d.)^[c]^	67 (39)	17 (33)
5	DIC/Oxyma	31 (31)	10 (40)	n.d. (n.d.)^[c]^	46 (<10)	<1 (n.d.)^[c]^
6	HATU/Et_3_N	34 (34)	10 (60)	85 (88)	88 (58)	1 (<1)
7	HBTU/Et_3_N	33 (33)	10 (20)	86 (82)	71 (55)	2 (9)
8	EDC⋅HCl/ Oxyma^[d]^	n.d.^[c]^	30	96	>99	<1

[a] Milling reactions were performed in a 5 mL polytetrafluoroethylene (PTFE) jar with three stainless‐steel balls (5 mm diameter) at 25 Hz. The total mass of reactants was 50 mg. The values in parentheses correspond to solution reactions. [b] LDL=undesired epimer; determined by HPLC. [c] n.d.=not determined. [d] Reaction was performed on a total mass of 250 mg of reactants in a 15 mL PTFE jar with one stainless‐steel ball (10 mm diameter) at 25 Hz for 30 min using 1.7 equiv. of EDC⋅HCl. EtOAc was used as a liquid additive instead of DMF.

Compared to the 30 min needed to complete the peptide coupling under classical solution conditions (Table 7), the mechanochemical procedure required only one‐third of this reaction time (10 min). The authors suggest that a higher concentration of the reaction mixture in the ball mill speeds up their reaction times than in solution. This mechanochemical protocol was also applied to longer peptide fragments, providing a valuable tool to synthesize oligopeptides with significantly higher efficiency (Table 8).

**Table 8:** Scope of peptide couplings by ball milling.^[a]^ Adapted with permission from Ref. [115a]. Copyright 2021, American Chemical Society.

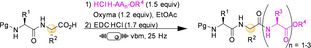				
Entry	Peptides	*t* [min]	Isolated yield [%]	*de* [%]
1	Cbz‐Ala‐Phg‐Phe‐OMe	20	89	>99
2	Cbz‐Ala‐d‐Phg‐Phe‐OMe	20	92	>99
3	Cbz‐Ala‐Cys(Bn)‐Ala‐OMe	30	98	>99
4	Cbz‐Ala‐Cys(Bn)‐Phe‐OMe	20	94	>99
5	Cbz‐Phe‐Val‐Cys(Bn)‐OMe	30	98^b^	>99
6	Cbz‐Phe‐d‐Val‐Cys(Bn)‐OMe	30	97	>99
7	Cbz‐Phe‐Val‐Ser(^*t*^Bu)‐O^*t*^Bu	30	97^b^	>99
8	Cbz‐Phe‐d‐Val‐Ser(^*t*^Bu)‐O^*t*^Bu	20	97	>99
9	Boc‐Trp‐Phe‐Glu(Bn)‐OBn	15	92	>99
10	Boc‐Trp‐Phe‐Gly‐OBn	10	84	>99
11	Boc‐Trp‐d‐Phe‐Gly‐OBn	10	95	>99
12	Cbz‐Phe‐Val‐Leu_2_‐OBn	25	95^[c]^	> 99
13	Cbz‐Phe‐d‐Val‐Leu_2_‐OBn	25	91^[c]^	98
14	Cbz‐Phe‐Val‐Leu_3_‐OBn	30	93^[c,d]^	>99
15	Cbz‐Phe‐d‐Val‐Leu_3_‐OBn	30	91^[c,d]^	99

[a] Milling reactions were performed in a 15 mL PTFE jar with one stainless‐steel ball (10 mm in diameter). The total mass of reactants was 250 mg. [b] 1.2 equiv. of EDC⋅HCl was used. [c] 1.0 equiv. of HCl⋅H‐AA_*n*_‐OR was used. [d] 2.2 equiv. of EDC⋅HCl, *η*(EtOAc)=0.9 μL mg^−1^, and total mass=267 mg. *de*=diastereomeric excesses.

Peptides and proteins are closely linked. Proteins constitute a class of molecules closely associated with living in an aqueous environment. For example, a mechanochemical methodology involving proteins has been developed by performing enzyme catalysis under mechanochemical conditions^.[116,12c]^ We are not aware of mechanochemical methodology involving covalent transformations of proteins. However, the ability to perform mixing in the absence of solvents allows for mechanochemical supramolecular modification of proteins. Milling can be employed to mix a water‐soluble protein powder with hydrophobic dyes.[^117^, ^118a^, ^119^, ^120^] When the resulting hybrid material is dissolved in water, the hydrophobic effect will act as a cohesive force keeping the hydrophobic dyes associated with the protein. In fact, this is a well‐known method for dispersing pigments in water.

However, such dispersions where a protein acts as a dispersing agent for a hydrophobic molecule/material present opportunity for the preparation of novel self‐assembled materials that would be challenging to prepare by other means. Many proteins have rich self‐assembly chemistry; for example, many proteins will self‐assemble into nanofibrils when heated in weakly acidic water.^[118]^

When employing proteins milled with hydrophobic dyes, the resulting dispersion will form nanofibrils incorporating the hydrophobic dye; or in other words, functionalized nanofibrils are formed. A wide variety of materials can be prepared, suitable for organic electronics or photonics applications.[^119^, ^120^] For example, functionalized fibrils have been incorporated into organic‐LEDs^[120d]^ or as coatings that can convert the light from a UV‐LED into white light.^[120a]^ In addition, if bioactive hydrophobic materials are employed, such functionalized protein materials may present opportunities to prepare materials suitable for applications such as drug delivery.^[117]^ In addition, it may be possible to employ other self‐assembling systems than proteins using this approach.

Few reagents have contributed to the general progress in improving humankind‘s health and economic well‐being as much as ammonia.^[121]^ NH_3_ is the crucial reagent in the (bio)synthesis of amino acids, proteins and is one of the most important feedstocks for fertilizers, plastics, explosives, and many other chemicals.^[122]^ In 2019, the total world production of ammonia reached 140 million tons worldwide, making it one of the world‘s top ten chemicals synthesized.^[123]^

Despite a growing demand for NH_3_, the Haber–Bosch process remains the state‐of‐the‐art method for preparing ammonia more than 100 years after its discovery.^[124]^ The process involves the reaction between gaseous molecular nitrogen (N_2_) and gaseous molecular hydrogen (H_2_) at high temperatures (400–500 °C) and pressures (100 bar). The Haber–Bosch process has a negative enthalpy change (Δ*H*=−46.22 kJ mol^−1^), and therefore according to Le Châtelier's principle, ammonia formation is less favored thermodynamically at high temperatures.^[125]^ Although low temperatures and pressures thermodynamically favor the equilibrium, the process in a 100‐year history has not changed significantly.^[126]^

**Figure 10:**
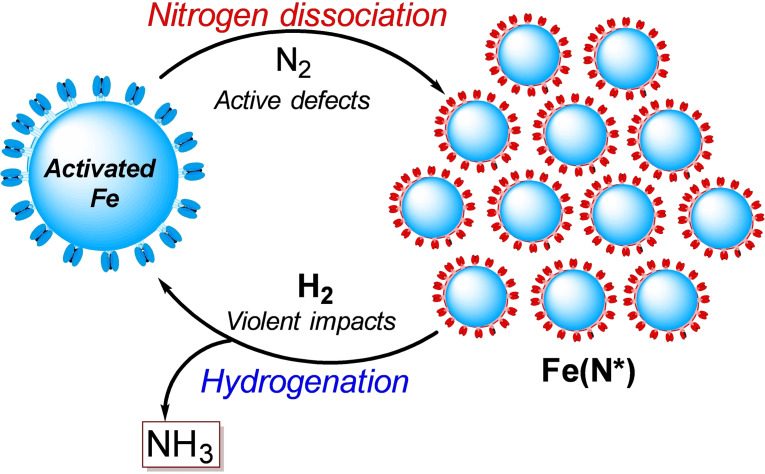
Schematic illustration of the ammonia synthesis process. Adapted with permission from Ref. [129a]. Copyright 2021, Springer Nature.

In the traditional Haber–Bosch process, according to the Brønsted–Evans–Polanyi relation,^[127]^ the elevated temperature promotes the dissociation of stable N_2_ molecules and facilitates the desorption of strongly adsorbed/entrapped intermediates (N*, NH*, and NH_2_*).^[128]^ However, mechanochemistry marks a break with traditional approaches as it has several unique properties, including metastable non‐equilibrium states, high impact force, and high defect densities that allow the reaction to occur using milder conditions (Figure 10).

Recently, Han et al.^[129a]^ have employed a mechanochemical approach for synthesizing ammonia at 45 °C and 1 bar by using an iron‐based catalyst, reaching the higher final ammonia concentration (82.5 vol%) than state‐of‐the‐art ammonia preparation under high temperature and pressure (25 vol%, 450 °C, 200 bar).

The mechanochemical process for ammonia synthesis consists of two steps. In the first step, repeated collisions generate active defects on the surface of the iron catalyst (Figure 10) and provide additional energy to promote the dissociation of stable nitrogen gas into nitrogenated iron particles [Fe(N*)]. In the second step, violent mechanical impacts involving the low‐coordinated defects on the catalyst surface promote both the hydrogenation of atomic nitrogen into NH_x_* species (*x*=1–3) and facilitate the final desorption of the strongly adsorbed NH_x_* intermediates from the iron surface, releasing the product ammonia (Figure 10).

The initial charge pressure of hydrogen strongly affects the ammonia yield and concentration, which reached as high as 82.5 vol% when the initial charge pressure was 2 bar. However, upon completion of the reaction, the remaining pressure was only 1.4 bar, which suggested that hydrogenation can proceed at near‐atmospheric pressure (1 bar).

As stated by the same authors, low‐temperature and pressure reaction conditions simplify the configuration of the manufacturing device, which does not need a heater, pressurizer, and/or other related systems. This also means safety can be improved. Thus, scaling up productivity at a low cost could be possible (204 and 937 mmol h^−1^ USD^−1^, Table 9). Moreover, this mechanochemical methodology changes the traditional approach to ammonia synthesis by allowing the construction of decentralized reactors on a flexible scale and near the point of consumption.

**Table 9:** Synthesis of ammonia: comparison with other methods. Adapted with permission from Ref. [129a]. Copyright 2021, Springer Nature.

Parameter	Mechanochemical	Haber‐Bosch	Electrochemical	Chemical looping	Photochemical
Ref.	[129a]	[129b]	[129c]	[129d]	[129e]
energy classification	mechanical	thermal	electrochemical	thermal	photochemical and thermal
reaction conditions	45 °C, 1 bar	400 °C, 1 bar	ambient	300 (350) °C, 1 bar	light, 1000 °C
product	82.5 vol% (gas)	0.37 vol% (gas)	10 ppm (electrolyte)	– (gas)	– (electrolyte)
yield [μmol h^−1^]	[1315 (N*)] [6073 (NH_3_)] 1081	554.3	1.6–2.4 μmol cm_geo_^−2^ h^−1^	93.75	0.20
yield [μmol g^−1^ h^−1^]	[55 (N*)] [253 (NH_3_)] 45	5543	0.8 μmol cm_geo_^−2^ h^−1^	3125	1.67
catalyst	Fe powder	Ni/LaN	Li on Mo	Ni–BaH_2_	Mg
raw metal price [USD kg^−1^}	0.27 (Fe)^[a]^	14 (Ni)^[a]^ 4.2 (La)^[b]^	82 (Li)^[a]^ 26 (Mo)^[a]^	14 (Ni)^[a]^ 894 (Ba)^[c]^	2.5 (Mg)^[b]^
catalyst price [USD kg^−1^]^[d]^	0.27	5.425	–^[e]^	454	2.5
yield [mmol h^−1^ USD^−1^]	[204 (N*)] [937 (NH_3_)] 168	1022	–^[e]^	6.9	0.67
consumed energy [J mt^−1^]	4.5×10^12^	39×10^12^	2.1×10^12^	–^[f]^	38×10^12^ (light) – (thermal)
separation or purification [J mt^−1^]	low^[g]^	low^[g]^	226×10^12,[h]^	low^[g]^	–^[i]^
total consumed energy [J mt^−1^]	4.5×10^12^	39×10^12^	228×10^12^	–^[f]^	38×10^12^ (light)^[j]^

[a] The price is from *Mineral Commodity Summaries*. https://www.usgs.gov/centers/nmic/nitrogen‐statistics‐and‐information (2020) [b] The price is according to https://www.metal.com. (Accessed on 20th August 2020). [c] There is no bulk commodity for elemental Ba. The price is from the Alfa Aesar with a pack of 1 kg (Catalog No. 010103.A1). [d] The preparation and non‐metal element costs are not considered here. Only the raw metal cost is included. [e] The used catalyst is a bulk electrode. The mass and composition are not given. [f] The power of the heating device is not given in the work. [g] The gas state ammonia can be readily separated by gas compression. [h] The product is ammonium, not ammonia. The cost of transferring ammonium to ammonia is not included. [i] The ammonia concentration in the electrolyte is not given. [j] The consumed energy of thermally reducing MgO to Mg is not included.

Despite the highly interesting finding, this approach could not be considered a proper catalytic Haber–Bosch process for the continuous ammonia synthesis at room temperature. Indeed, in this mechanochemical protocol, ammonia has been synthesized by nitridation of iron and subsequent hydrogenation of the *in‐situ* generated iron nitrides. Despite ammonia's extreme importance and challenging nature, attempts to develop continuous Haber–Bosch ammonia synthesis under milder conditions have failed, or they were of limited interest and relevance.

**Figure 11:**
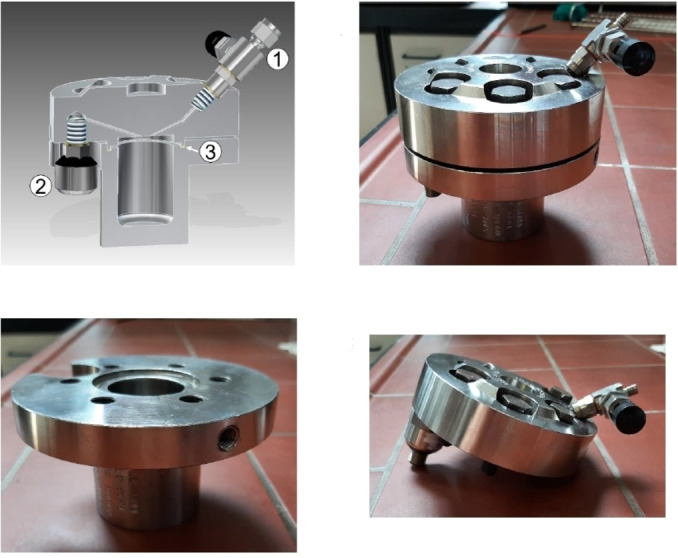
Drawing and picture of the milling jar used for the batch experiments. Reproduced with permission from Ref. [130]. Copyright 2021, Wiley‐VCH.

In an exhaustive study, Schüth and co‐workers^[130]^ have recently developed a remarkable procedure for the mechanocatalytic synthesis of ammonia from H_2_ and N_2_, working continuously at room temperature and under atmospheric pressure. The process was first developed in a batch approach, using stainless‐steel homemade jars that resembled a traditional autoclave in their exterior design (Figure 11). These devices allowed to rapidly screen different catalyst systems at room temperature (pressures >100 bar), following experimental conditions that ideally meet the thermodynamic requirements for ammonia synthesis.

The first grinding attempts with elemental iron were ineffective. In contrast, the addition of alkali metal ferrites AFeO_2_ (A=Li, K, Cs) to the grinding mixture enabled the detection of the formation of ammonia (up to 0.25 vol%, for LiFeO_2_). Unfortunately, subsequent powder X‐ray diffraction (PXRD) analysis of powders from the Fe/CsFeO_2_ reaction disclosed the presence of CsOH⋅H_2_O, indicating an *in‐situ* reduction product of CsFeO_2_ under the formation of water. As a result, the authors assumed that the ammonia produced during the milling process resulted from nitride hydrolysis and non‐catalytic reaction with the reaction water.

**Figure 12:**
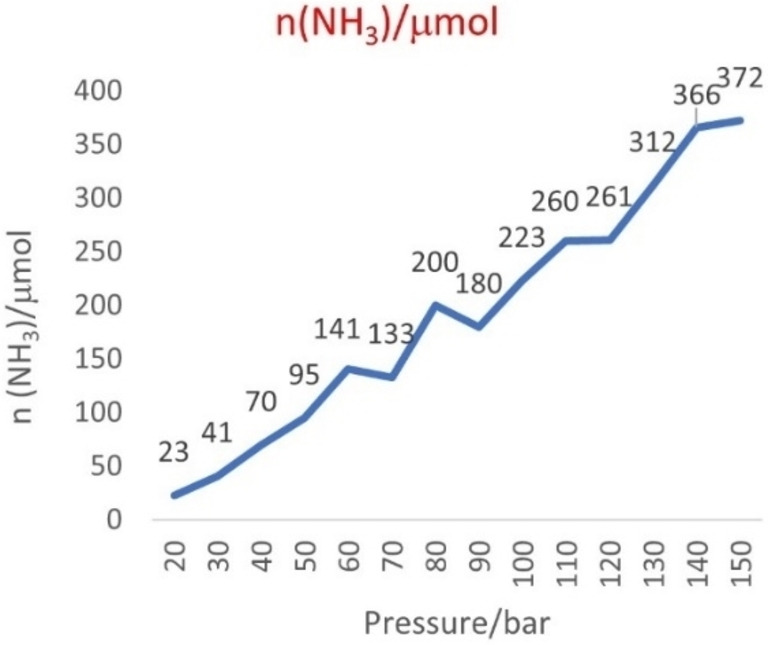
Pressure‐dependency of the mechanocatalytic ammonia formation in batch. In each experiment, a fresh FeCs mixture was used, total milling time for each experiment was 24 h. Adapted with permission from Ref. [130]. Copyright 2021, Wiley‐VCH.

On the contrary, the addition of cesium (2.2 mol % Cs, a total of 2.0 g catalyst) to iron allowed to prepare ammonia not only at 150 bar [24 h of milling, *n*(NH_3_)=0.378 mmol, *Y*=0.38 %, with *n*(NH_3_) as the total amount of ammonia produced and the yield *Y* based on nitrogen) but also at 50 bar [same reaction conditions, *n*(NH_3_)=0.097 mmol, *Y*=0.29 %). The authors attributed this to the higher electropositivity of Cs and thermodynamic instability of the bulk cesium nitride (Cs_3_N), while, in the case of lithium nitride (Li_3_N), it resulted very stable. As expected, this mechanochemical version of the Haber–Bosch process also has a favorable response to increased pressure (at room temperature, the system is far from equilibrium), resulting in an ammonia increase (Figure 12).

**Figure 13:**
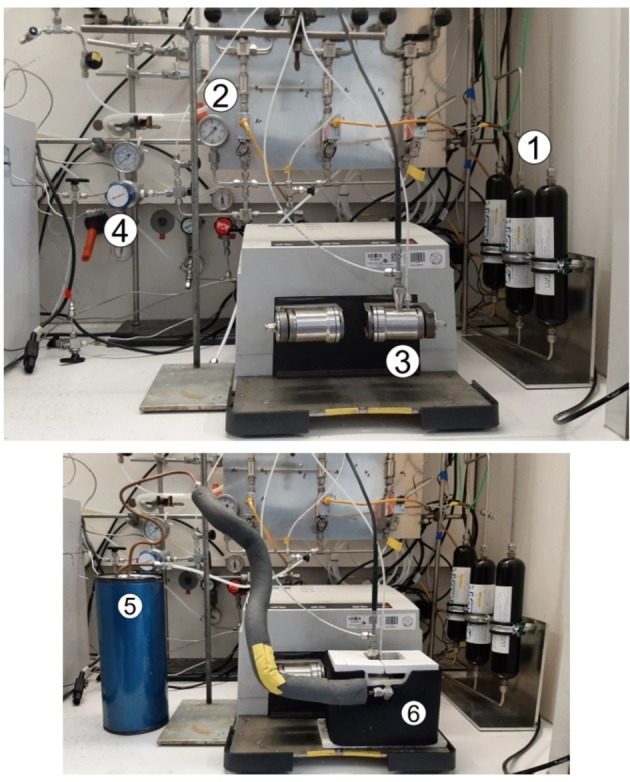
Set‐up for the continuous mechanocatalytic ammonia synthesis. (1) Inlets of individual gases, additionally purified by Oxiclear gas‐purification columns. (2) Dosing of gases using mass‐flow‐controllers, manometer. (3). Modified shaker mill with home‐built jar equipped with thermocouple and gas connections. (4) Back‐pressure regulator. (5) Dewar with a copper spiral for cooling down nitrogen gas to control the milling jar‘s average temperature. (6) Cooling box made out of fiberglass sheets. Reproduced with permission from Ref. [130]. Copyright 2021, Wiley‐VCH.

For studying the influence of some reaction parameters of a continuous mechanochemical‐activated flow process, such as temperature, pressure and residence time, special milling jars were designed that resemble small autoclaves (Figure 13).

**Figure 14:**
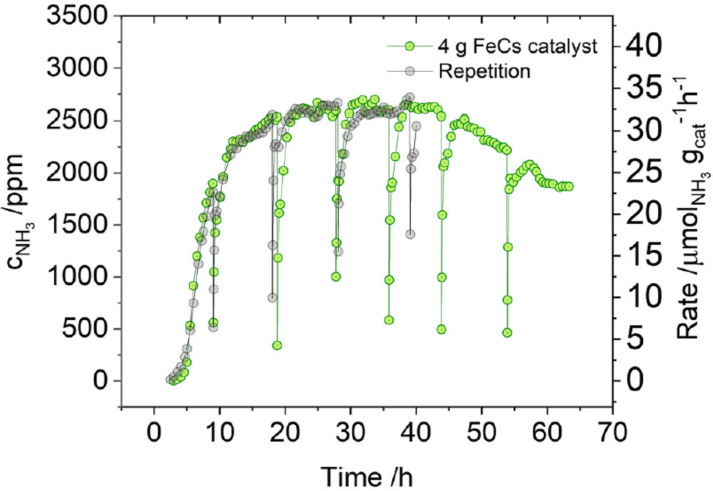
Development of ammonia production during continuous mechanocatalysis with 4.0 g of the catalyst. The experiments were performed using a shaker mill at a frequency of 25 Hz and with two 15 mm steel balls. The temperature was kept at 20 °C by external cooling. The jar was fed with 20 mL min^−1^ (STP) of an H_2_/N_2_ (3 : 1) mixture at 20 bar. Due to safety reasons, the milling process had to be paused every day, which is the reason for the sharp spikes. Reproduced with permission from Ref. [130]. Copyright 2021, Wiley‐VCH.

At 20 bar pressure (3H_2_+N_2_), and using a jar fed with iron (3.8 g) and cesium (0.2 g), this mechanocatalytic version of the Haber–Bosch process allowed to develop, for the first time, operating both in batch and in a continuous process the formation of ammonia at ambient temperature (global vessel temperature was kept at 20 °C by a cooling system) up to 0.26 vol% for more than 60 h (Figure 14).

After more than a hundred years of ineffective efforts via different approaches, this study proves the continuous synthesis of ammonia (more than 0.2 vol% for over 50 h) from its elements (H_2_ and NH_3_) in the presence of a cesium‐promoted iron catalyst at ambient temperature and pressure conditions, following a Haber–Bosch similar process. It is important to emphasize that these breakthroughs have been made possible only thanks to the influence of mechanical forces, a scenario that just a few years ago would have been unthinkable.

### 2.3 Mechanochemical processes developed in the presence of (solid) reagents used as a gas source

In the collective imagination, ball mill machines, regardless of their working principle, are valuable devices for comminution, crushing minerals and pulverizing solid raw materials to a fine powder. However, it is more recent knowledge that the mechanical energy developed during collisions inside a jar can promote a reaction between reactants, at least one of which has to be in a solid state.

**Scheme 20:**
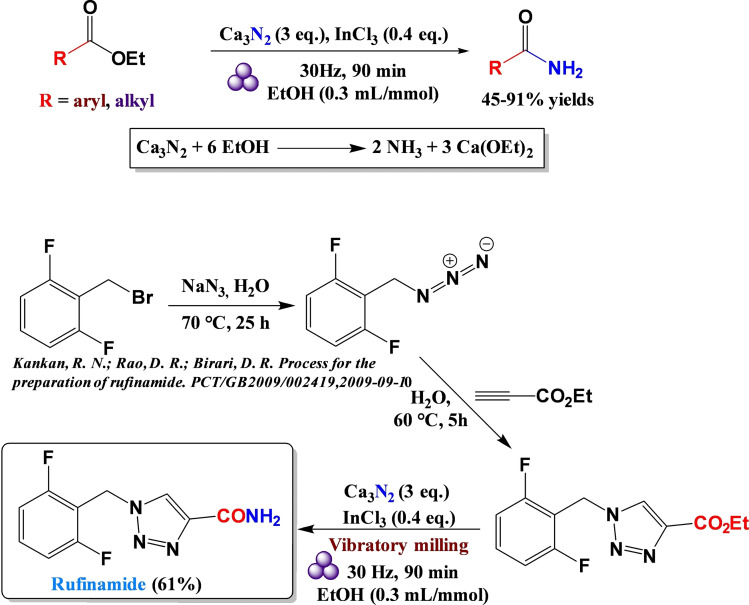
Amidation conditions and relative scope. Adapted with permission from Ref. [131].

In the context of nitrogen generation capable of reacting with organic compounds, Menéndez and co‐workers also contributed with a swift and easy reaction via ball mill.^[131]^ When calcium nitride is ball milled with an ester, under the mild conditions reported in Scheme 20, it afforded the corresponding amide product in good to high yields. The group made a broad scope of aromatics, heteroaromatics and amino acid compounds, demonstrating the technique's potential. In addition, to validate the potential application in industrial processes, they reported the synthesis of a widespread and commercialized anticonvulsant drug, rufinamide (Scheme 20).

**Figure 15:**
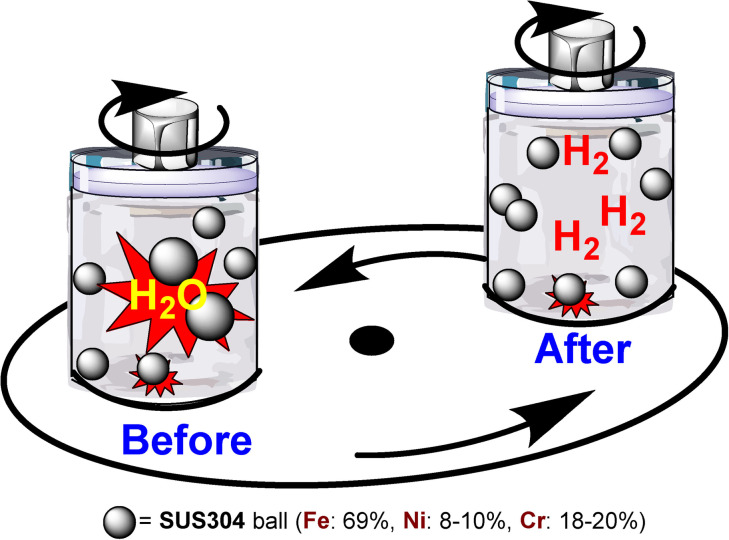
Hydrogen generation from water through ball milling. Adapted with permission from Ref. [132a].

Sajiki and co‐workers^[132a]^ turn this widespread belief and prove that mechanochemical collision of stainless‐steel (SS) balls using a planetary ball mill device could generate hydrogen gas from water, alkane, and diethyl ether without CO_2_ emission. They conducted systematic studies on hydrogen generation reactions using various ball mill machines. Still, a high‐energy planetary ball mill's milling regime at 800 rpm released enough mechanical energy to promote hydrogen production from water (Figure 15).

All reactions were conducted using stainless‐steel beads (diameter=5 mm) and vessels (80 mL) having a specific chemical composition in metals (SUS304: ≈69 % Fe, 8–10 % Ni, and 18–20 % Cr), which turned out to be critical both in the generation of hydrogen and in subsequent reactions where hydrogen was used in reduction processes.

The grinding process of 15 mmol of water at a rotational speed of 800 rpm led to a significant increase in temperature and pressure inside the jar, which reached a plateau within an hour. Furthermore, GC–MS analysis showed that the internal gas derived from the H_2_O was almost exclusively hydrogen (14.75 mmol), without molecular oxygen, which is a dangerous detonating gas.

**Scheme 21:**
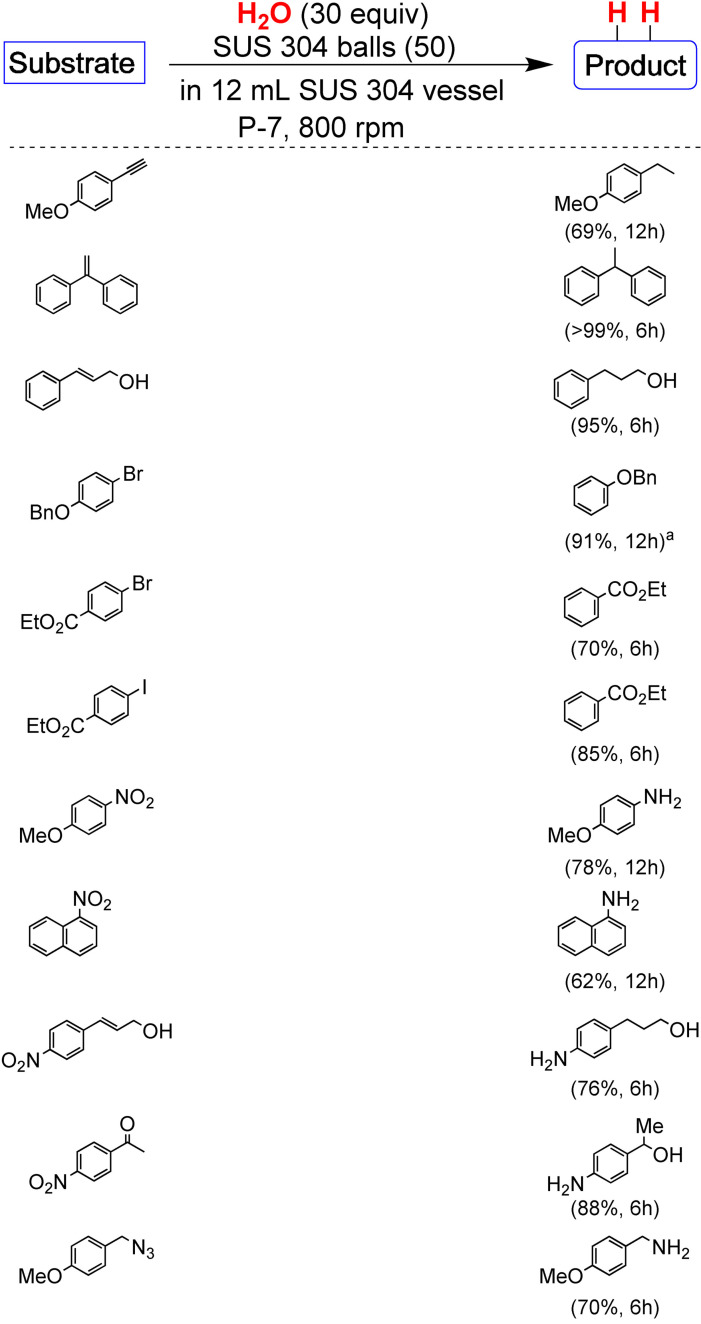
Scope of substrates used in hydrogenation. [a] Mixture of the unchanged substrate and product (9 : 91) was obtained. Adapted with permission from Ref. [132a].

Interestingly, ethyl ether and other hydrocarbons can also be used as feedstock to generate hydrogen *in situ*. Once such a mechanochemical procedure has been developed, the authors have successfully implemented this protocol for the reduction/hydrogenation of multiple bonds, nitro groups, ketones, halides, and azide residues (Scheme 21). The formation of a black powder follows generating hydrogen from water using SUS304 balls and a vessel.

**Scheme 22:**
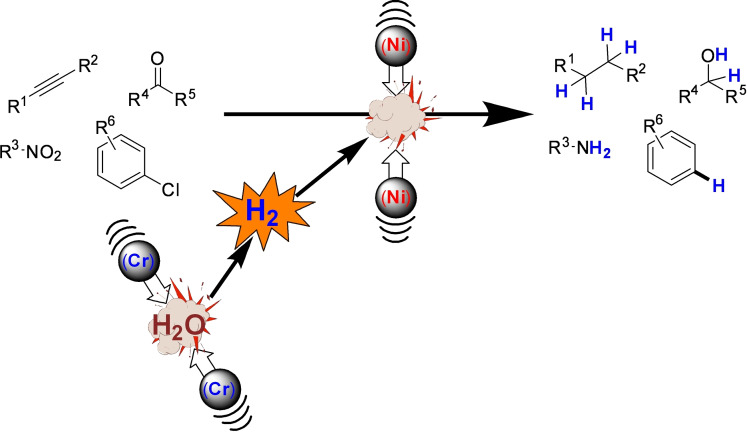
Cr‐mediated H_2_ generation and Ni‐catalyzed mechano‐hydrogenation on SUS304. Adapted with permission from Ref. [132a].

X‐ray photoelectron spectroscopy (XPS) analysis on the most superficial part (sputtering time of 0 min)^[133]^ of the fresh SUS304 powder suggests that Cr^0^ is the reducing species responsible for hydrogen generation from water (Scheme 22). At the same time, the most abundant iron metal induces regeneration of the Cr^0^ species by single‐electron transfer. The abundance ratio of Fe^0^ relatively decreased, while the Ni^0^ metal has no change in percentage composition. Finally, Ni^0^ can act as a catalyst for subsequent hydrogenation reactions.^[134]^

In recent years, mechanochemistry allowed to development of reactions handling potentially dangerous gaseous reactants such as H_2_,^[135]^ O_2_,^[136]^ CO,^[137]^ CO_2_,^[138]^ HCN,^[139]^ C_3_H_6_,^[140]^ and CH_4_,^[141]^ always operating safely for the operator.^[142]^ Carbon monoxide, no doubt, is one of the most widely used chemical reagents in organic synthesis in both academic and industrial fields. Although CO is an inexpensive reagent and plays a crucial role in many organic applications, it is an odorless, toxic, and flammable gas. Moreover, health and safety concerns regarding its storage and transport severely restrict its enormous potential.

Although a considerable number of papers involving the use of gaseous CO for the carbonylative reaction are reported in the literature, there is still a certain reluctance on the part of operators to handle this highly toxic gas. This problem becomes more relevant when palladium‐catalyzed carbonylations require pressurized CO.

To mitigate this problem, carbonylation procedures that use metal carbonyls, such as **M**(CO)_6_ (**M**=Cr, Mo, W) and Co_2_(CO)_8_, have received increasing attention as alternative and convenient carbonyl sources avoiding the need for directly handling gaseous carbon monoxide.^[143]^

Although these solid CO sources are extremely attractive as they can release CO on demand, in solution, they demand high temperatures and high‐boiling solvents (diglyme, 1,4‐dioxane, DMSO, anisole, etc.). Previous studies have shown that Mo(CO)_6_, a reliable CO‐releasing solid reagent, spontaneously decomposes into CO and Mo(s) at temperatures above 150 °C and 1 atm pressure.^[144]^

**Scheme 23:**
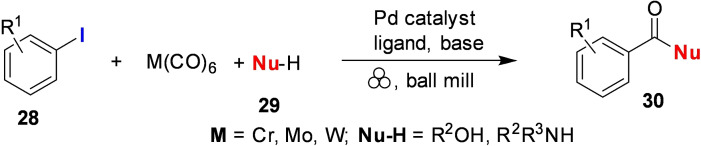
Mechanochemical carbonylation reactions by ball milling. Adapted with permission from Ref. [145b]. Copyright 2020, Wiley‐VCH.

In this regard, Hernández and co‐workers^[145]^ have developed palladium‐catalyzed alkoxycarbonylation and aminocarbonylation reactions in ball mills using molybdenum hexacarbonyl as a versatile one‐carbon building block with the ability to generate *in situ* carbon monoxide during the reaction (Scheme 23). The reaction proceeded smoothly at room temperature and did not require particular skills or further operator training beyond the necessary safety precautions.

The procedure was first set up (Table 10) by milling iodobenzene (0.2 mmol) with butanol (0.4 mmol) in the presence of various CO‐releasing solid reagent [M(CO)_6_=0.2 mmol]. Once all process parameters were tuned, Mo(CO)_6_ proved to be the most effective in forming benzyl benzoate (Table 10, entry 2).

**Table 10:** Screening of metal carbonyls M(CO)6 (M=Cr, Mo, W) in the mechanochemical carbonylation of **28 a** with *n*‐butanol **29 a**.^[a]^ Adapted with permission from Ref. [145b]. Copyright 2020, Wiley‐VCH.

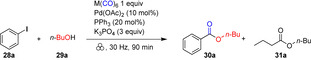			
Entry	M(CO)_6_	Yield **30 a** ^[a]^ [%]	Yield **31 a** ^[b]^ [%]
1	Cr(CO)_6_	13	26
2	Mo(CO)_6_	99	<1
3	W(CO)_6_	22	26

[a] Reaction conditions: **28 a** (0.2 mmol), **29 a** (0.4 mmol), M(CO)_6_ (0.2 mmol), Pd(OAc)_2_ (0.02 mmol), PPh_3_ (0.04 mmol), and K_3_PO_4_ (0.6 mmol) were ball‐milled in a 5 mL stainless‐steel milling jar with one 10 mm milling ball of the same material. [b] Determined by ^1^H NMR spectroscopy using ethylbenzene as the internal standard.

On the contrary, Cr(CO)_6_ and W(CO)_6_ promote a competitive oxidative homocoupling reaction of *n*‐butanol, which leads to the formation of a significant amount of butyl butyrate (26 %, Table 10, entry 3).

K_3_PO_4_ plays a dual role both as a base for alcohol deprotonation and a promoter by boosting the release of carbon monoxide from the hexacarbonyl metal source.

Further studies by the same authors showed that the released carbon monoxide is promptly intercepted by the palladium catalyst for the subsequent carbonylation reaction without significant release of molecular CO in the jar. Thus, the palladium‐catalyzed carbonylation reaction affects both kinetics (i. e., the release/generation of carbon monoxide and its consumption), avoiding the direct handling and exposition to highly toxic and hazardous gaseous CO.

### 2.4 Mechanochemical processes developed in the presence of raw ores used as metal catalysts

Many raw ores are earth‐abundant minerals in nature and play an important role as mediators and catalysts in promoting a wide variety of complex organic reactions.^[146]^ These include, for example, the formation and cleavage of C−C and C−H bonds, the synthesis of organic nitriles from terminal alkynes and trimethylsilyl azide, and the selective hydrogenation of a large set of functionalized nitroarenes to substituted anilines, among others.^[147]^

The effectiveness of metals in accomplishing their catalytic activity is often related to their unique morphology and elemental composition, which has inspired the design of new materials intending to mimic such structures. However, the poor solubility of minerals in commonly used reactive media strongly restricts their application. It represents the main bottleneck to the full spread of these methodologies based on cheap minerals as heterogeneous catalysts. Mechanochemistry overcoming the issues closely related to the low solubility of reagent has brought new life to this methodology, which was far from a mainstream choice in catalysis research.

**Scheme 24:**
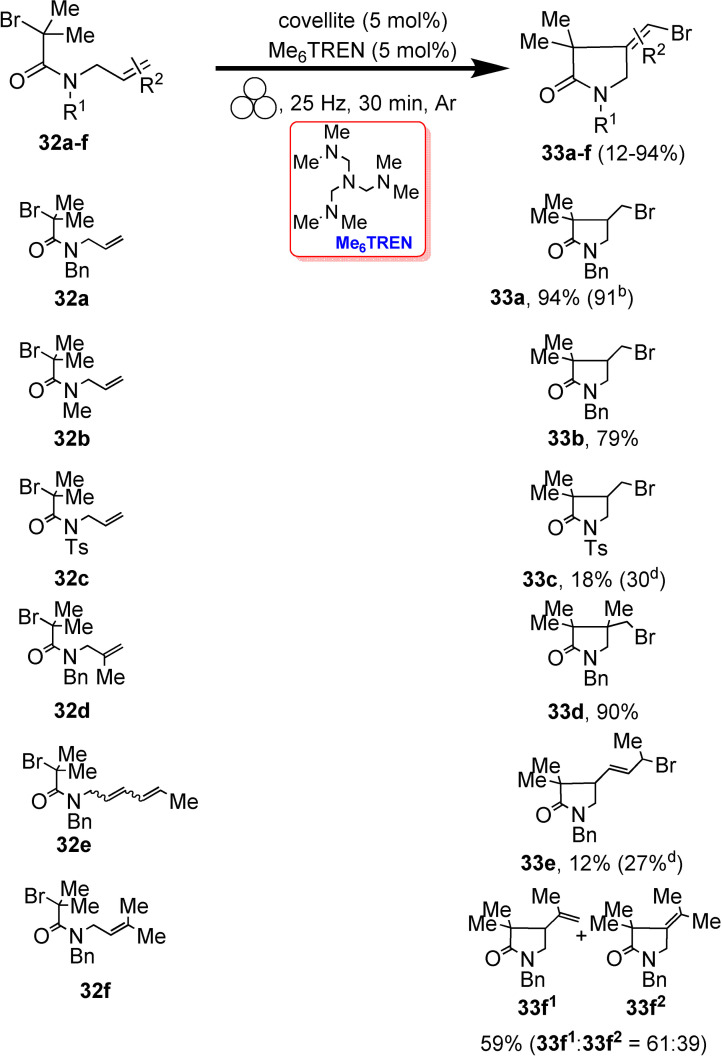
Scope of ATRC reaction. [a] Reaction conditions: **32 a**–**32 f** (0.3 mmol or 100.0 mg), covellite (0.02 mmol, 5 mol %), and Me_6_TREN (0.02 mmol, 5 mol %) were milled under argon in a 10 mL ZrO_2_‐Y milling jar with one 10 mm milling ball of the same material for 30 min at 25 Hz. [b] Reaction conducted on 1.0 mmol scale of **32 a**. [c] 90 min of milling. Adapted with permission from Ref. [148]. Copyright 2020, American Chemical Society.

**Scheme 25:**
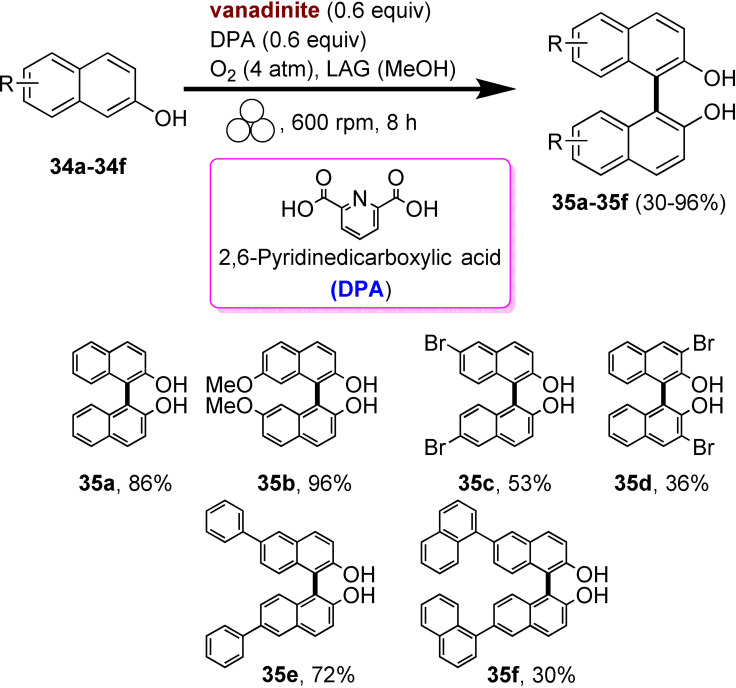
Scope of mechanochemical vanadium‐catalyzed oxidative coupling. Reaction conditions: **34 a**–**34 f** (0.70 mmol), vanadinite (252.5 mg; 0.43 mmol), DPA (71.5 mg, 0.43 mmol), and methanol (*η*=0.05 μL mg^−1^) were milled under dioxygen in a planetary ball mill using a 20 mL ZrO_2_‐Mg milling jar with five 10 mm milling balls of the same material for 8 h at 600 rpm (1 h×8; 20 min pause after each milling cycle). Adapted with permission from Ref. [148]. Copyright 2020, American Chemical Society.

In a recent article, Bolm and co‐workers^[148]^ highlighted the potential of using raw mineral ores as catalysts in copper‐catalyzed atom transfer radical cyclizations (ATRC) and vanadium‐catalyzed oxidative couplings. In this study, covellite (CuS), a binary copper sulfide mineral, and vanadinite [Pb_5_(VO_4_)_3_Cl], a vanadium‐based mineral, effectively promote atom transfer radical cyclizations (Scheme 24) and oxidative couplings of β‐naphthol derivatives (Scheme 25 ), respectively. The mechanochemical reactions proceed in the presence of a catalytic amount of mineral either under neat conditions or by using a minimal amount of solvent (LAG, MeOH, *η*=0.05 μL mg^−1^). To best perform their catalytic activity, the metals within the mineral ores require the use of some readily available organic ligands such as Me_6_TREN and DPA (Schemes 24 and 25).

### 2.5 Mechanochemistry in redox processes promoted by piezoelectric materials

In organic chemistry, redox processes are one of three types of reactions (addition reactions, substitution reactions, and elimination reactions) that govern most reactions and are still one of the most widely studied topics in chemistry today.^[149]^

**Scheme 26:**
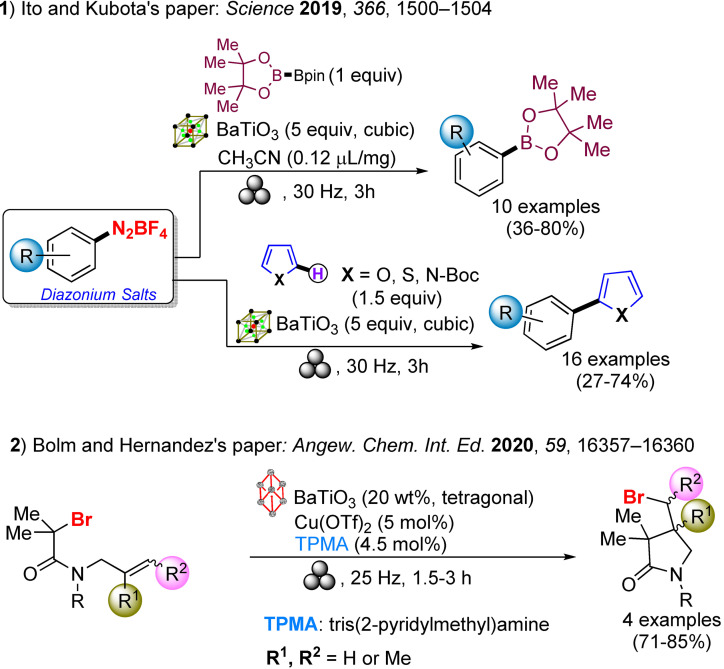
Application of mechanochemistry in redox processes. Adapted with permission from Refs. [154] and [155]. Copyright 2020, the authors.

In recent years, the growing interest in this class of reactions got an important role in promoting the development of suitable synthetic protocols to ensure efficient redox protocols^[150]^ and minimize the environmental impact of these synthetic strategies.^[146]^ In this context, we observed a resurgence of proof concepts developed about a century ago by Ciamician and based on sunlight or some of its components as the green energy source.^[151]^ The combination of ever more efficient LEDs and more effective catalysts has led to an explosion of photochemical technologies.^[152]^ Unfortunately, scaling up the photochemical processes is the first real bottleneck of all these modern photocatalytic reactions, limiting the application of the photocatalysis system.^[153]^ In 2019, the road for the application of mechanochemistry in redox processes was paved,^[154]^ followed by two independent contributions, published almost synchronously, with Ito and co‐workers^[154]^ and Bolm and co‐workers^[155]^ showing the use of piezoelectric materials (e. g., BaTiO_3_) for radical synthesis (Scheme 26). It is a topic so new and innovative for the panorama of organic synthesis that it is still to be explored. However, at least from a conceptual point of view, the process is straightforward and considerably broadens the fields of application of mechanochemistry.

The mechanical action of the balls on piezoelectric materials temporarily generates highly polarized redox species promoting the efficient transfer of an electron to an appropriate chemical oxidant and reception of an electron from a suitable reductant to move electrons towards small organic compounds.^[156]^

To further simplify the concept, the piezoelectric material works as a shuttle moving electrons from and to small organic molecules with or without an additional catalyst. These unusual features make mechano‐redox methodologies a cost‐effective and environmentally benign alternative to photocatalytic chemistry for the oxidation of organic substrates. However, despite the simplicity of this methodology, the number of papers produced on this topic is low; some questions remain and need to be better understood.^[157]^

During the preparation of the present manuscript, a noteworthy contribution by Cao and co‐workers^[158]^ contributed significantly to shedding light on this subject by broadening its scope. They devised a mechanochemical‐induced strategy to synthesize a library of 1,2‐diketoindolizine derivatives from indolizines and epoxides by using BaTiO_3_ as the piezoelectric material (Table 11).

**Table 11:** Optimization of reaction conditions.^[a]^ Adapted with permission from Ref. [158a]. Copyright 2021, American Chemical Society.

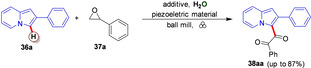				
Entry	Additive	Piezoelectric material (particle size)	Jar/ball material	Yield^[b]^ [%]
1	PivOH	none	SS/SS	10
2	PivOH	BaTiO_3_	SS/SS	60
3	TFA	BaTiO_3_	SS/SS	43
4	AcOH	BaTiO_3_	SS/SS	40
5	ZnCl_2_	BaTiO_3_	SS/SS	trace
6	PivOH	BaTiO_3_	SS/PTFE	32
7	PivOH	BaTiO_3_	PTFE/PTFE	20
8	PivOH	BaTiO_3_	PTFE/SS	51
9	PivOH	BaTiO_3_ (<3 μm)	SS/SS	87
10	PivOH	ZnO (<50 nm)	SS/SS	trace
11	PivOH	TiO_2_ (<3 μm)	SS/SS	10
12	PivOH	BaCO_3_ (<5 μm)	SS/SS	12
13^[c]^	PivOH	BaTiO_3_ (<3 μm)	SS/SS	84
14^[d]^	PivOH	BaTiO_3_ (<3 μm)	SS/SS	85

[a] Conditions: **36 a** (0.3 mmol), **37 a** (0.6 mmol), piezoelectric material (0.6 mmol), and H_2_O (0.6 mmol) were added in a 20 mL milling jar with 30 balls (6 mm Ø) for 6 h at 550 rpm. [b] Determined by GC–MS analysis. [c] 12 h. [d] Piezoelectric material (1.5 mmol).

These investigations suggested that PivOH appears to be the most effective additive for this transformation (Table 11, entries 3–5).

**Scheme 27:**
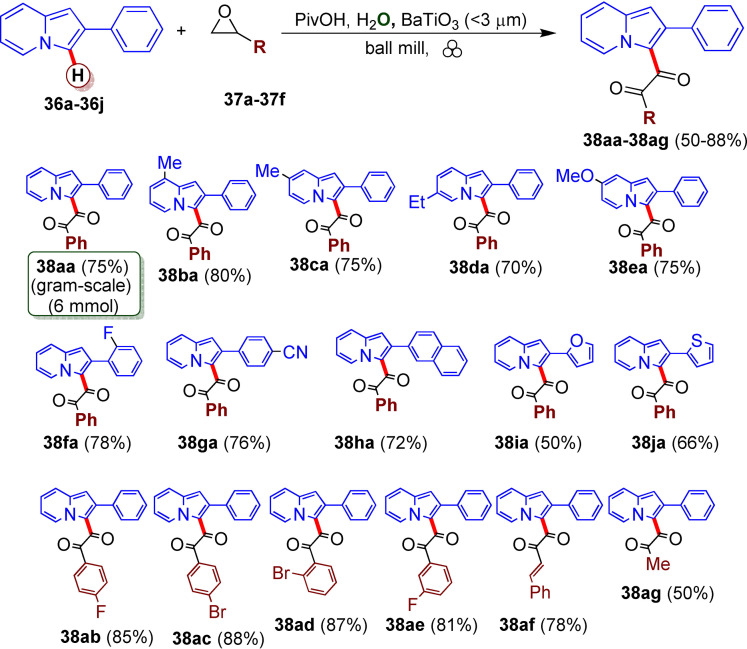
Representative control experiments. Conditions: **36** (0.3 mmol), **37** (0.6 mmol), PivOH (0.3 mmol), BaTiO_3_ (<3 μm, 0.6 mmol), and H_2_O (0.6 mmol) were added in a 20 mL milling jar with 30 balls (6 mm Ø) for 6 h at 550 rpm. Isolated yield. Adapted from Ref. [158a]. Copyright 2021, American Chemical Society.

At the same time, cubic BaTiO_3_ (particle size: <3 μm), generating more polarized particles in response to mechanical stimuli, improved the catalytic efficiency of this mechanochemical process, affording the product **38 aa** in high yield (87 %, Table 11, entry 9). All these mechano‐redox reactions proceeded smoothly and efficiently to give the corresponding 1,2‐diketoindolizine derivatives in fair to good yields, also on a large scale (Scheme 27).

Compared with other solvent‐based procedures, non‐use of solvents, simple experimental conditions, the ability to quickly scale up the process, and high conversion efficiency are some of the features that make this procedure a greener and more practical approach for preparing indolizine structures.

**Scheme 28:**
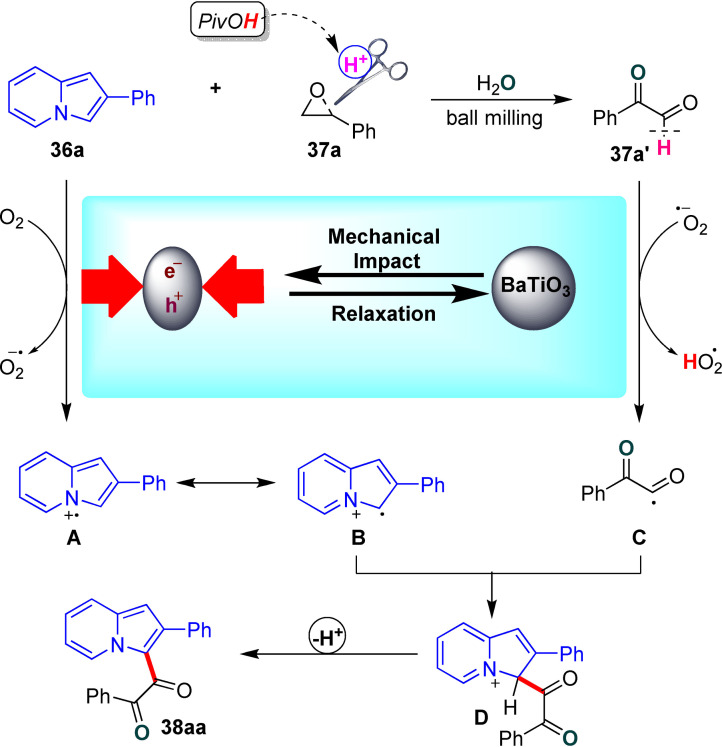
Suggested mechanism. Adapted with permission from Ref. [158a]. Copyright 2021, American Chemical Society.

Based on experimental results and several literature reports, the authors hypothesize that the process proceeds following a radical oxidation route, as shown in Scheme 28.

### 2.6 Mechanochemistry processes performed with homemade single‐screw drill device

This roundup of examples presents a cross‐section of papers addressing new advances in mechanochemistry and its potential to design new reaction pathways by providing access to new chemical structures, often never prepared before. That said, there remains an underlying question that is well summed up in one of Henry Ford's many aphorisms: “*There is no true progress if technology is not within range of everybody*”.^[159]^

**Figure 16:**
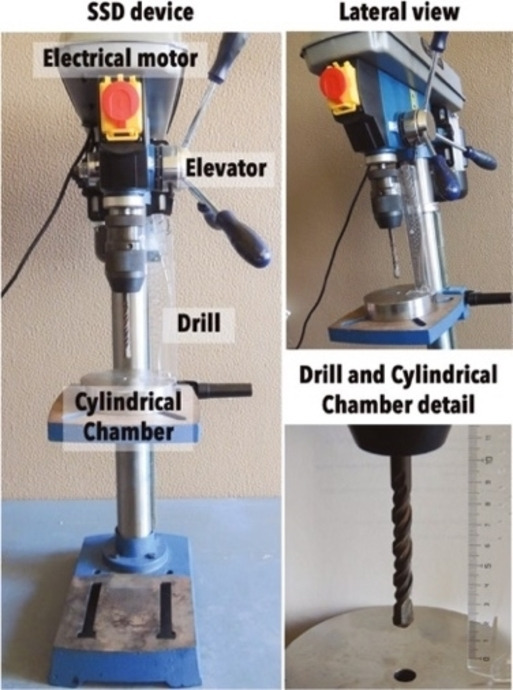
SSD device for mechanochemistry. Reproduced with permission from Ref. [160]. Copyright 2020, the authors.

The development of dedicated reactors is still today a challenge. In 2020, Pineiro and co‐workers^[160]^ have shown that some well‐known mechanochemical processes can be developed, even with better performance, using an inexpensive single‐screw drill (SSD) (10 cm long and 1 cm of diameter) rotating in a closed fixed stainless‐steel cylindrical chamber (2.5 cm deep and with 1.05 cm of diameter (capacity 4.33 cm^3^) column trap equipped with a steel tip and a hole (Figure 16).

**Scheme 29:**
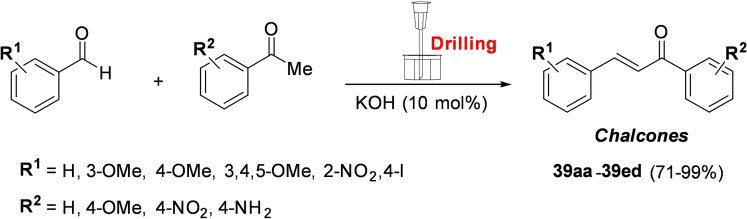
Effect of substituents on aldehydes and acetophenones on the mechanochemical synthesis of chalcones. Yields obtained by drilling aldehyde (1.0 mmol), PhCOCH_3_ (1.0 mmol), and KOH (10 mol %), in the SSD device for 5 min followed by recrystallization from ethanol. The drill is driven by a direct drive electric motor at 250 rpm. Adapted with permission from Ref. [160]. Copyright 2020, the authors.

**Scheme 30:**
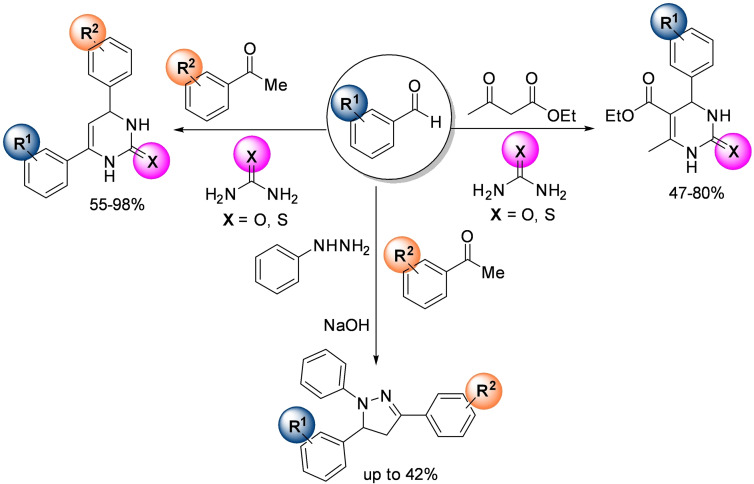
Multicomponent reactions for the synthesis of dihydropyrimidinones, dihydropyrimidinethiones, and pyrazolines under mechanical activation. Adapted with permission from Ref. [160]. Copyright 2020, the authors.

**Scheme 31:**
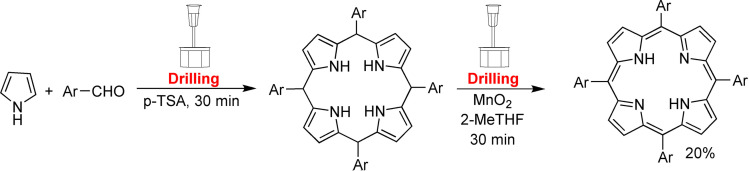
Two‐step synthesis of 5,10,15,20‐tetrakis(4‐methoxyphenyl)porphyrin. Adapted with permission from Ref. [160]. Copyright 2020, the authors.

The authors successfully applied this homemade single‐screw drill device to prepare chalcones **39** (Scheme 29), dihydropyrimidinones, thiones, pyrazoline (Scheme 30), and porphyrins (Scheme 31). The reactions were performed at room temperature in higher yields and shorter reaction times than similar mechanochemical protocols in commercial ball milling devices.^[161]^

This widespread and straightforward automatic device combines the simplicity of the classical mortar with the reproducibility of the ball mills. The movement of the drill device, rotating (250 rpm) in the escape direction, imparts intense shear stress and turbulent levels, letting cavitation and intense mixing between reagents under the so generated high‐pressure conditions.

Noteworthy, the reaction among benzaldehyde (1 mmol), acetophenone (1 mmol), and solid powdered KOH (10 mol %) to give the corresponding chalcone proceeded smoothly in the new mechanical device. The mechanosynthesis took about 5 min to complete compared to the 10 min required in a classic vibrating ball milling system operating at 25 Hz.^[162]^ The GC–MS analysis of the reaction crude confirmed the formation of the chalcone as a product, while no evidence of self‐condensation secondary products was detected. The SSD device allowed to scale up (5×) this classical Claisen–Schmidt reaction in the total absence of a solvent. The use of KOH in catalytic amounts (10 %) makes it possible to cut the *E*‐factor to 0.17 (0.39 in solution^[163]^), which is the lowest value ever achieved before for the synthesis of this class of compounds.

Significant sustainability scores in terms of both *E*‐factor and Eco‐Scale were also achieved for all the other reactions taken into analysis. In particular, the Biginelli synthesis performed in a single‐screw drill device halved the *E*‐factor (0.22) compared to similar procedures in solution (*E*‐factor=0.5^[164]^).

### 2.7 Mechanochemically‐conducted Grignard reactions

**Figure 17:**
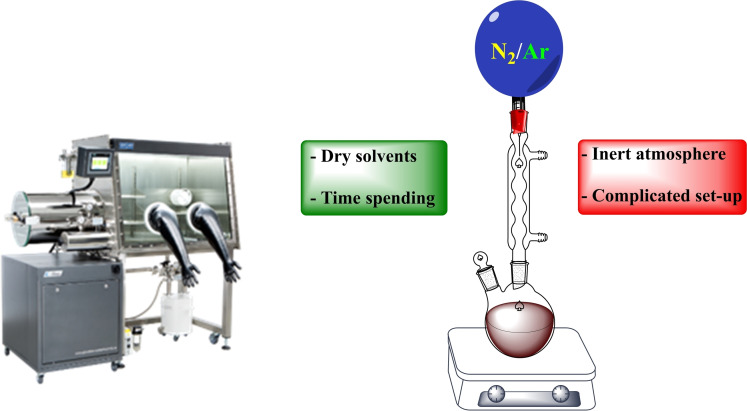
Typical conditions for Grignard's reaction in solution.

With the aim of facilitating the approach to chemistry, trying to set in the best reaction conditions and avoiding a whole series of complicated combinations, Ito and co‐workers have developed an innovative and extremely versatile method for the preparation of Grignard reagents and their consequent reactions on various electrophiles.^[165]^ Grignard's reaction has always been a helpful tool for preparing certain intermediates and products within well‐defined retrosynthetic pathways. However, if this is true, it is equally valid that these reactions are often difficult to implement, requiring inert and anhydrous atmospheres without considering the preparation of complicated set‐ups (Figure 17), discouraging their use.

**Scheme 32:**
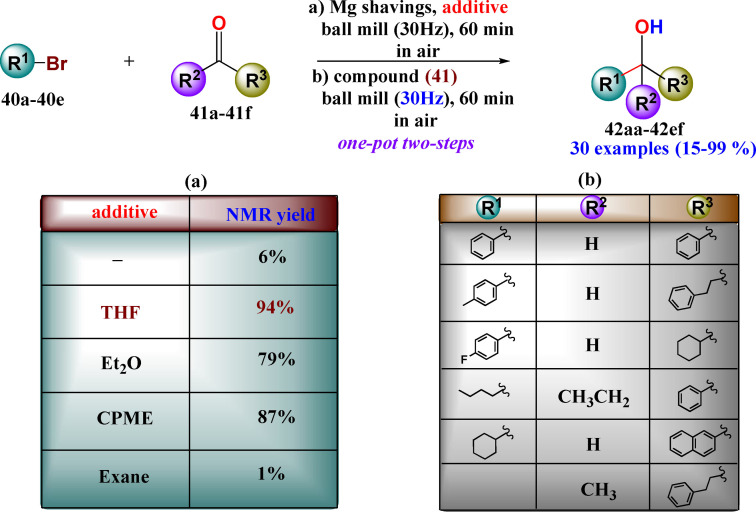
Conditions optimization and reaction scope. Adapted with permission from Ref. [165]. Copyright 2021, the authors.

Before their work, several attempts for this type of reaction were tried via the ball mill, resulting in failures or limited outcomes, yield, and scopes.^[166]^ For the first time, Ito and co‐workers found a way to overcome these difficulties by using the mechanical force and without any restriction about dryness or typical environmental reaction conditions. There are two main advantages, above all the other improvements that this technique furnishes: the first is the simple set‐up required with this approach, which is less time spent and much more direct. The other point is broadening the reaction scope, facilitating access to different reaction products in tight dependence on the reaction conditions. The first study optimized the organo‐magnesium nucleophile synthesis as its object, which provided significantly better results in the presence of additives, such as ethereal solvents (e. g., THF and cyclopentyl methyl ether), in a 1 : 2 ratio. Once the best parameters were found, a large scope was carried out, including aromatic and aliphatic bromides as the nucleophile substrate and aromatic and aliphatic carbonyl compounds (aldehydes and ketones) as partners for the reaction (Scheme 32).

The above‐described methodology worked perfectly with liquid bromide reagents, but very low yields were obtained when the halides were solids. Since neither prolonging time nor varying additive and LAG species worked, increasing the temperature was the only chance. Based on the experience gained in their cross‐coupling work_,_ ^[66]^ Ito and co‐workers performed the reactions using a heat gun to reach a precisely controlled temperature of 68 °C (measured inside the jar with a thermographic analysis). Thanks to this, not only did the technique work for the first time in the ball mill, but it also helped to overcome the problems related to all these reactions that in solution did not proceed due to the poor or the lack of solubility of the compounds, demonstrating once again the strength of the methodology.

**Scheme 33:**
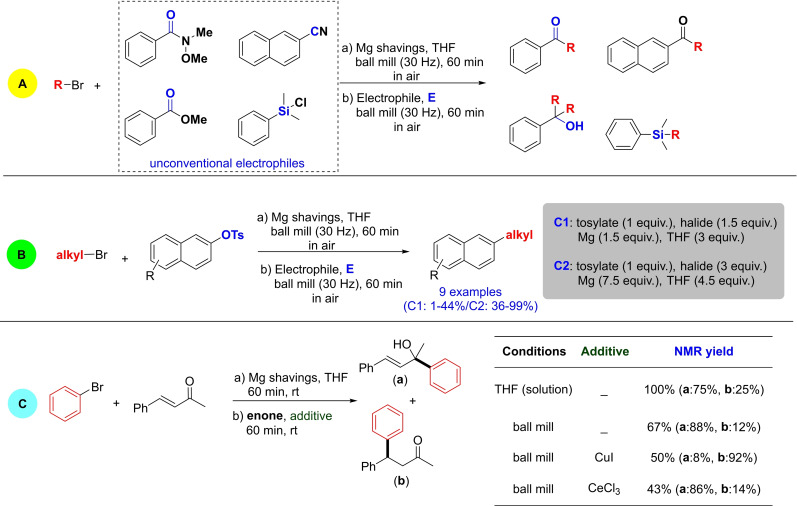
Expansion of the reaction's scope. Adapted with permission from Ref. [165]. Copyright 2021, the authors.

As previously mentioned, Grignard's reaction is a highly versatile tool with unique perspectives in a broad context of synthetic plans, even in a solid‐phase approach. The results, in general, were impressive (Scheme 33), especially in the case of enones: depending on the adopted approach, whether it is a reaction in solution or one via ball mill, but performed with different additives, there is a change in the selectivity of the preferred center for the attack (1,2‐addition or 1,4‐addition) (Scheme 33).

**Scheme 34:**
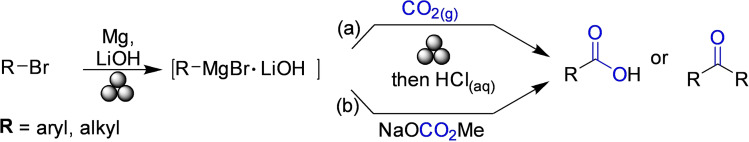
Mechanochemically conducted Grignard reactions with CO_2_ (a) and sodium methyl carbonate (b). Adapted with permission from Ref. [169]. Copyright 2022, the authors.

In 2020 and 2021, the interest in Grignard reagents prepared by mechanical activation or used in mechanochemical processes has been proven by a growing number of relevant publications on this topic,[^166c^, ^167^] quickly becoming an important and hot topic.^[168]^ In this context, Bolm and co‐workers recently developed a one‐pot, three‐step mechanochemical procedure for preparing Grignard reagents from aryl and alkyl organobromides in a ball mill.^[169]^ Their subsequent reactions with gaseous carbon dioxide (CO_2_, Scheme 34a) or sodium methyl carbonate (SMC, Scheme 34b) provided the corresponding carboxylic acids in moderate to good yields (up to 82 %, Scheme 34).

**Scheme 35:**
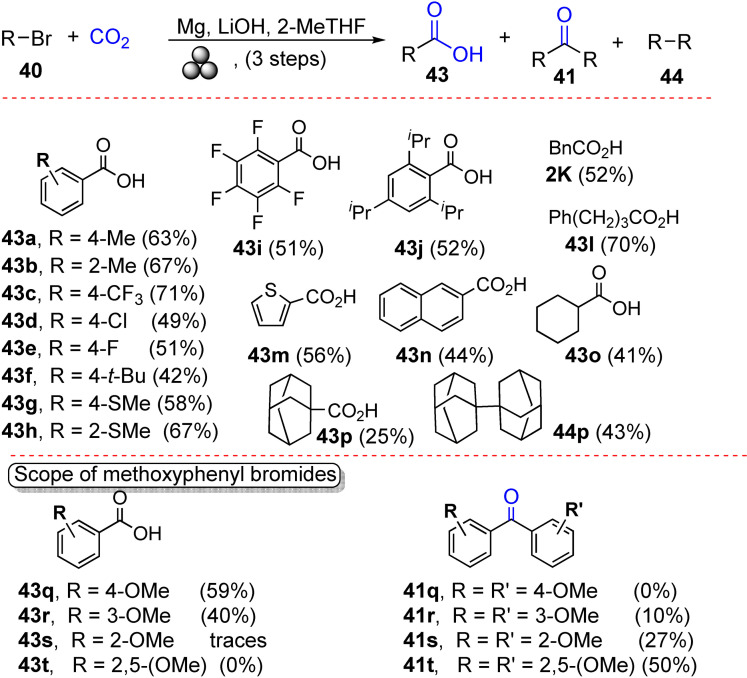
Scope of organobromides in mechanochemical Grignard reactions with CO_2_ (4.0 mmol scale). Reaction conditions (for steps I–III): step I=Mg (2.5 equiv.) and LiOH (1.1 equiv.) in a ZrO_2_‐M milling vessel (20 mL) with gas inlet/outlet valves and 5 ZrO_2_‐M balls (Ø=10 mm) under Ar; step II=addition of 2‐MeTHF (2.0 equiv.) and **40** (4.0 mmol) under Ar; step III=CO_2_ (4 bar). The yields refer to product amounts obtained after column chromatography. Adapted with permission from Ref. [169]. Copyright 2022, the authors.

An interesting reaction variation was observed using methoxy‐substituted aryl bromides, where significant amounts of symmetric ketones were isolated as major products, strongly depending on the position of the methoxy substituents in the aryl bromides (Scheme 35). These results contrast with what has already been reported in the literature for analogues in solution‐based Grignard reactions.^[170]^

In contrast to analogues solvent‐based procedures, this protocol needs the presence of only 2 equiv. of an ethereal additive (THF, 2‐MeTHF) as LAG. The authors preferred 2‐MeTHF (derived from renewable biomass)^[171]^ as it has proven to have better performance in various organometallic reactions, preventing thermal runaway reactions and suppressing Wurtz couplings of benzyl halides.

As expected, lithium chloride as an activating agent in the first grinding step for preparing the so‐called “Turbo‐Grignard reagents” improved the yield of **43 a** from 25 to 60 %.^[172]^ Surprisingly, a screening of lithium salts pointed out that LiOH was superior to LiCl (63 % of **43 a**), which is commonly used in solution as an additive to promote the Grignard reactions.

The authors attribute this improvement by LiOH, which has neither background nor counterparts in solvent‐based chemistry, “*to the unusual reaction conditions lacking standard interactions between a possible magnesiate and the surrounding solvent*”.

**Scheme 36:**
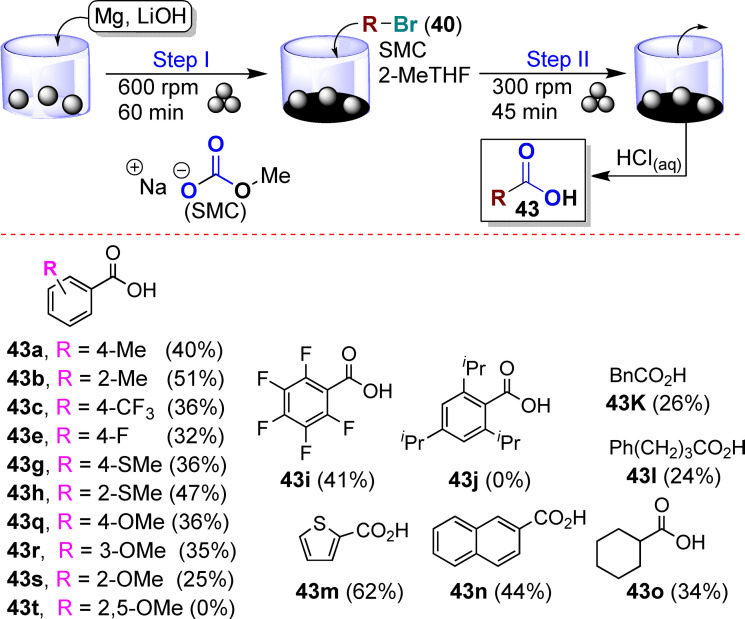
Scope of aryl and alkyl bromides in a mechanochemical Grignard reaction with SMC (1.0 mmol scale). Reaction conditions: step I=Mg (2.5 equiv.) and LiOH (1.1 equiv.) in a ZrO_2_‐M milling vessel (12 mL) with 3 ZrO_2_‐M balls (Ø=9 mm); step II=addition of 2‐MeTHF (2.0 equiv.), SMC (1.5 equiv.), and **40** (1.0 mmol). The yields refer to product amounts obtained after column chromatography. Adapted with permission from Ref. [169]. Copyright 2022, the authors.

The use of SMC as a solid and benchstable CO_2_ surrogate shows several undeniable advantages since an entire step shortened the milling procedure, and argon gas was not required. Finally, the overall process time was significantly shorter. Symmetrical ketones **41** were also detected with SMC, but generally, this reactivity is less pronounced (Scheme 35 and Scheme 36).

**Scheme 37:**
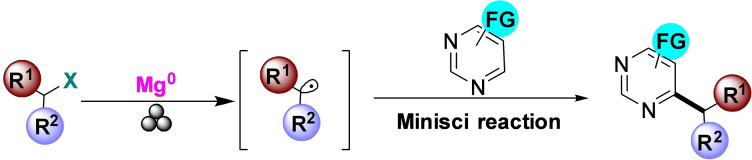
New approach to access alkyl radicals by mechanochemistry. Adapted with permission from Ref. [173]. Copyright 2021 l, American Chemical Society.

In recent work of outstanding scientific significance, Yu and co‐workers^173^ demonstrated that Grignard reagents mechanochemically prepared from alkyl halides could be employed for the regioselective construction of diversely functionalized pyrimidines by a Minisci‐type reaction (Scheme 37).^[174]^ As inexpensive commercially available chemical building blocks, alkyl halides could be considered the ideal choice to promote radical‐mediated C−H functionalizations by homolytic cleavage of the labile C−X bond.^[175]^ In the Minisci‐type reactions, the generation of unstabilized alkyl radicals is crucial for directing the pathways of subsequent reactions and thus controlling the results of the whole process.

**Scheme 38:**
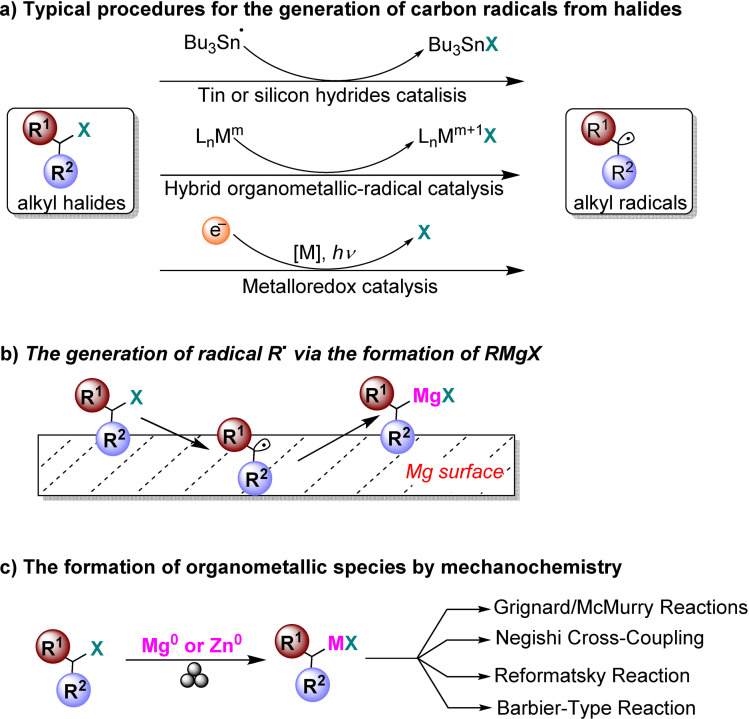
Classical chemical strategies for preparing carbon radicals from unactivated alkyl halides. Adapted with permission from Ref. [173]. Copyright 2021 l, American Chemical Society.

Classically, all the approaches that have been proposed in the literature to date, although effective, have several shortcomings, such as the use of precious metals, ligands, high temperature, strongly reducing systems, and others (Scheme 38).^[176]^

In this context, the mechanochemical interactions between freshly generated magnesium surfaces and alkyl halide reagents promote the formation of alkyl radicals that, in turn, are caught by pyrimidines rather than undertaking classical Grignard reactions. (Table 12, entry 1).

**Table 12:** Optimization of mechanochemical magnesium‐mediated Minisci reaction between **45 aa** and **46 aa**. Adapted with permission from Ref. [173]. Copyright 2021 l, American Chemical Society.

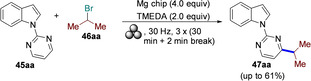		
Entry	Deviation from standard conditions	**3 aa** ^[a]^ [%]
1	none	61
2	without Mg chip	n.r.
3	without TMEDA	15
4	3.0 instead of 4.0 equiv. of Mg chip	38
5	1.0 instead of 2.0 equiv. of TMEDA	32
6	Mg foil/powder instead of Mg chip	36/17
7	Et_3_N/DMEDA/DBU instead of TMEDA	19/44/46
8^[b]^	THF/1,4‐dioxane as LAGs	51/51

[a] Yield of isolated product. [b] LAG (20 μL, *η*=0.3 [V (liquid; μL) m^−1^ (solid reagents; mg)]) was added.

**Scheme 39:**
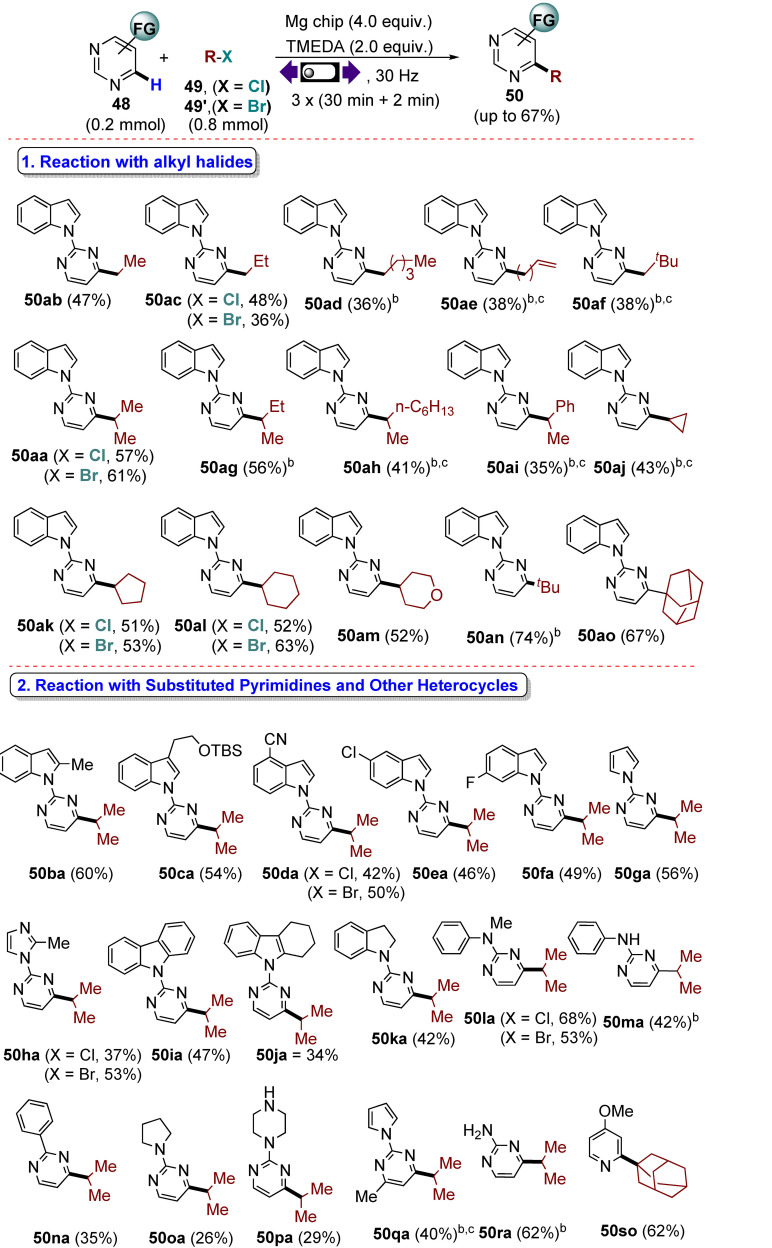
Mechanochemical magnesium‐mediated Minisci reaction between **48** and alkyl chlorides **49** and bromides **49’**. [a] All yields are isolated yields. [b] Milling at 30 Hz for [4(30 min+2 min break)]. [c] **49** (1.0 mmol), Mg chip (5.0 equiv.). Adapted with permission from Ref. [173]. Copyright 2021 l, American Chemical Society.

Afterwards, the standard protocol was further explored on various substrates (halides and nitrogen‐rich heterocyclic compounds), proving the applicability, usefulness, and generality of this mechanochemical Minisci‐type protocol (Scheme 39).

**Scheme 40:**
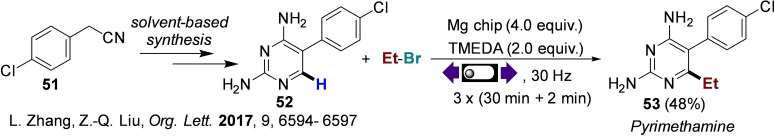
Synthesis of pyrimethamine **53** over 4 steps. Adapted with permission from Ref. [173]. Copyright 2021 l, American Chemical Society.

In sharp contrast to solution chemistry,^[177]^ where a mixture of products is usually obtained in this type of reaction, this mechanochemical procedure afforded the desired outcomes with excellent regioselectivity. This strategy's broad and practical applicability has also been demonstrated by the scaled‐up synthesis of **50 aa** from **48 a** (6 mmol) and preparation of pyrimethamine (**53**), an antimalarial drug, in 48 % yield over four steps (Scheme 40).

Mechanistic studies have demonstrated that the reaction follows a radical pathway, effectively ruling out the formation of alkyl magnesium halide.

### 2.8 Mechanochemical Kabachnick–Fields reaction

**Scheme 41:**
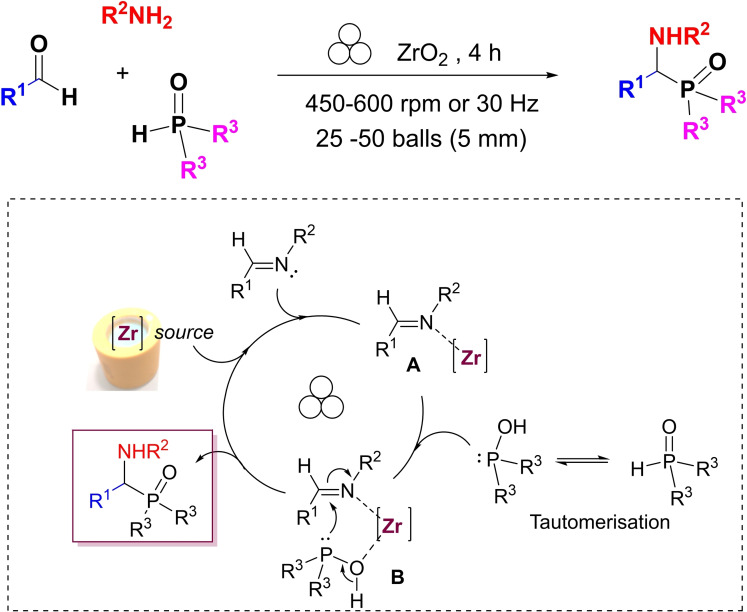
Mechanochemical KF‐3CR to prepare α‐aminophosphonates (R^3^=Oalkyl) or α‐aminophosphine oxides (R^3^=Ph). Reproduced with permission from Ref. [178]. Copyright 2020, American Chemical Society.

Mechanochemical activation was also applied to the preparation of α‐amino phosphonate backbone P(=O)−C−N via the three‐component domino Kabachnick^−^Fields reaction (KF‐3CR), intrinsically endowed with high atom economy, generating water as a by‐product, and allowing to the introduction, all‐in‐one, of C‐, N‐, and P‐modifications on the α‐amino phosphonate framework (Scheme 41).^[178]^

Therefore, α‐aminophosphonates (R^3^=Oalkyl) or α‐aminophosphine oxides (R^3^=Ph) were prepared by ball‐milling by reacting to an hydrophosphoryl compound (e. g., dialkyl phosphite or phosphine oxide), an aldehyde, and an arylamine. The final products were recovered pure upon precipitation in water and filtration under vacuum, selectively and in very high yields (95 % to quantitative), whatever the arylamine/arylaldehyde/P‐nucleophile combination was [P‐nucleophiles: P−(O)H(OEt)_2_, P(O)H(O−*i*Pr)_2_, and P(O)H(Ph)_2_]. The method was particularly successful in the preparation of α‐aminophosphine oxides (R^3^=Ph), a class of compound usually poorly investigated in solution. Indeed, the reactions conditions required large excess of amine, high temperature, and long reaction times, hampering the stability of the starting diphenylphosphine oxide reactants, generating the corresponding oxidized diphenylphosphinic acid byproduct, to be separated by a chromatographic column. In the same study, the kinetic constant associated with the imine formation was determined (*k*=0.0343 min^−1^) by a mathematical model^[179]^ applied to data recorded by the *in‐situ* monitoring of the reaction by Raman spectroscopy.^[175]^

The 3CR‐KF reaction outcome depended on the hardness and density of the grinding material. Therefore, when stainless steel was used, the reaction stopped to the intermediate *E*‐imine, while in ZrO_2_ the α‐amino phosphonate framework was obtained. The authors suggested that surface‐mediated phenomena involving the jar material (and the reactants) could influence the activation process by (i) accelerating the imine formation (usually slow in solution); (ii) generating *in situ* of a more electrophilic C=N bond (intermediate **A**); (iii) promoting the P‐tautomerization equilibrium (usually slow in solution) and (iv) exerting a template effect (intermediate **B**) favoring the hydrophosphonyation (addition of the P‐nucleophile) (Scheme 41).^[178]^ Moreover, by‐products such as α‐hydroxyphosphonates, their rearrangement to phosphates and amine‐promoted decomposition, and α‐amino bis‐phosphonates formed in solution were not observed.

When the reaction was performed in solution, under thermal activation in a sealed Pyrex tube (up to 70 °C under reflux in THF, CH_3_CN, and neat conditions, for 5 h), the conversions remained moderate, and the formation of the corresponding α‐hydroxyphosphonates could be observed. These results clearly demonstrated that thermal activation alone was not enough for achieving full conversions, selectivity, and high yield of α‐amino phosphonate derivatives, and post‐reactional treatments were always necessary (liquid–liquid extraction and purification by chromatography).

**Scheme 42:**
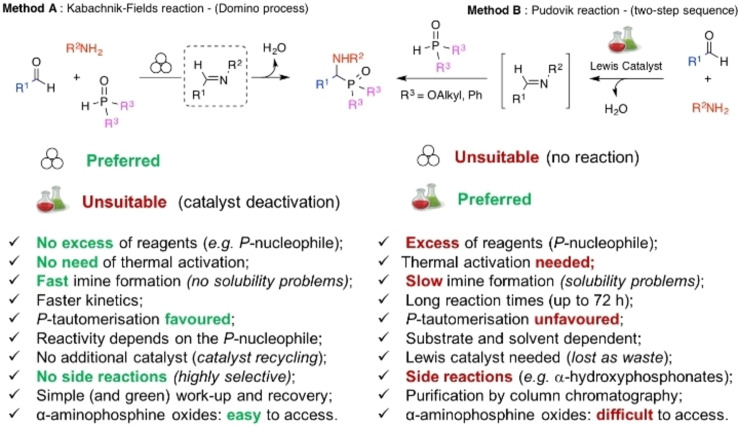
Comparisons between the KF‐3CR (method A) and Pudovik (method B) by ball‐mill and in solution, to prepare α‐aminophosphonates (R^3^=Oalkyl) or α‐aminophosphine oxides (R^3^=Ph). Adapted with permission from Ref. [178]. Copyright 2020, American Chemical Society.

Additionally, in solution the method of choice for the preparation of α‐aminophosphonates or α‐aminophosphine oxides is the step‐wise two‐component Pudovik reaction (method B, Scheme 42). A Lewis catalyst is needed to promote the addition of the P‐nucleophile on the C=N bond of the preformed imine. However, when investigated in the ball‐mill in ZrO_2_, the Pudovik reaction failed and the *E‐*imine was recovered unreacted and with no traces of *E*/*Z*‐isomerization. These results clearly showed that by simply eliminating the solvent from a protocol and transposing the reaction in a ball mill is not trivial in terms of outcome and that the reaction conditions usually disfavored in solution (e. g., the 3CR‐KF), became the method of choice by mechanochemistry, to access α‐aminophosphonates or α‐aminophosphine oxides, and presenting several advantages over the Pudovik method in solution (Scheme 42).^[178]^

### 2.9 Mechanosynthesis with solvent‐free separations

Nowadays, the mechanosynthesis of organic compounds has made it possible to design and synthesize compounds with different structural architectures and possible useful applications. Handling organic functional groups and converting them into others is often a laborious process, resulting in mixtures of compounds requiring subsequent further purification. Mechanochemical strategies can minimize solvent input, but subsequent purification steps often cannot avoid conventional solvent‐based purification steps, undermining the starting assumptions of solvent‐free procedures. This issue remains the bottleneck for all‐new synthesis technologies that aim to reduce solvent today. There is no one‐size‐fits‐all solution for all chemical processes, and the path in this challenging area is still far from definitive.

**Scheme 43:**
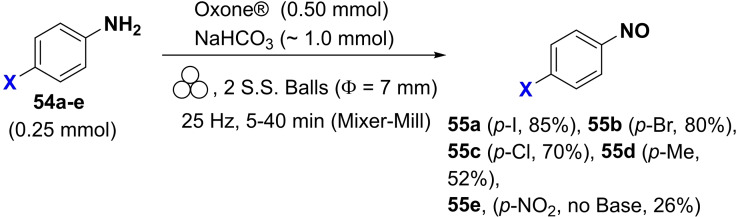
Mechanochemical preparation of nitroso derivatives from anilines. Adpated with permission from Ref. [180a]. Copyright 2012, Royal Society of Chemistry.

In this context, Vančik and co‐workers^[180]^ combined the mechanochemical preparation of nitroso derivatives with a fully solvent‐free purification process, addressing one of the more significant problems of milling mechanochemistry (Scheme 43).

They proved a rapid and selective solvent‐free synthesis of nitrosobenzenes under mechanochemical conditions using Oxone as a green solid oxidizing agent. This procedure avoids all issues associated with the high reactivity and ease of oxidation of nitrosobenzene derivatives observed in traditional solution‐based chemistry. The nitrobenzene targets could be easily isolated and purified by sublimation of the reaction crude collected directly from the grinding jar. A similar “solvent‐free” recovery of the final product was also reported for the mechanochemical preparation in batch of two APIs, nitrofurantoin and dantrolene.^[180b]^

### 2.10 Mechanochemical reactivity in the solid state

In several paragraphs of this Review, we highlighted how mechanochemical processes fulfil the twelve principles of green chemistry and make it possible to overcome reactivity problems arising directly or indirectly from using a solvent. However, we often lose sight of a crucial point of mechanochemical reactions: two or more solids are involved in the reactive process, and other factors/parameters, different from those observed in solvent‐based processes, play a crucial role during milling.

**Scheme 44:**
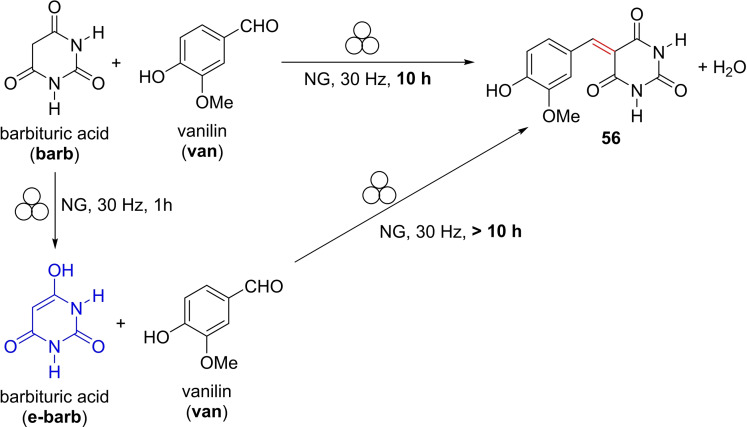
Knoevenagel condensation between barbituric acid (barb) and vanillin (van) starting from two keto tautomer of barbituric acid (desmotropes). Adapted with permission from Ref. [181]. Copyright 2021, American Chemical Society.

In a recent noteworthy study on the reactivity between two or more solid components, Halasz and co‐workers^[181]^ showed that the solid form of the reagents strongly influences their solid‐state reactivity when they are stripped of the external contribution of the solvent. They investigate a classical Knoevenagel condensation reaction between vanillin (van) and barbituric acid (barb), emphasizing as solid forms of the last reactive component could affect reaction paths, product selectivity and kinetics (Scheme 44). In the 1 : 1 barb:van cocrystal (bv), it was found that the formation of the condensation product **56** starts after around 1.5 h and goes to completion after a prolonged milling reaction period (10 h).

**Figure 18:**
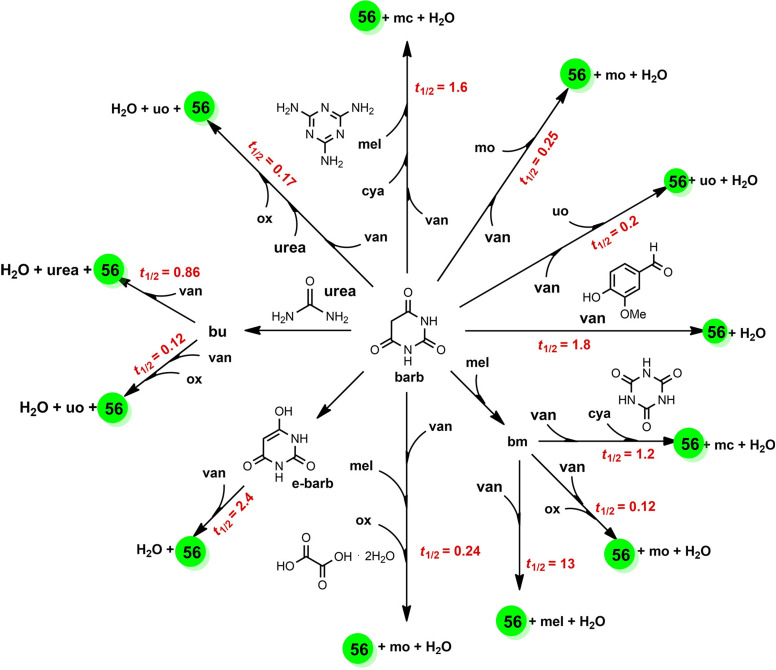
Modifications of barb and employed reaction conditions, including the approximate half‐life for each reaction (*t*_1/2_, [h]). Acronyms: barbituric acid (barb or b); cyanuric acid (cya or c); melamine (mel or m), oxalic acid (ox or o), urea (u); vanil (van or v), barbituric acid:urea cocrystal (bu), barbituric acid‐melamine salt (bm), melamine:cyanuric acid cocrystal (mc), vanillin:anhydrous oxalic acid (vox). Reproduced with permission from Ref. [181]. Copyright 2021, American Chemical Society.

On the contrary, the grinding process of vanillin and e‐barb, which is the enol and more stable form of the barbituric acid in the solid state, provided **56** at a slower rate (Scheme 44). The progress of the reaction was monitored by time‐resolved *in‐situ* Raman spectroscopy. By using a set of polymorphs, desmotropes (molecule in different tautomeric forms), cocrystals, or salts, the authors achieved not only around the 10‐fold acceleration of the reaction but also deceleration of the same mechanochemical process (Figure 18). Although many features of this process still need further investigation, these data pointed out faster reactions were promoted in a reaction environment with facile proton transfer. They were outstandingly skilled in systematically manipulating the chemical reactivity through applying principles of crystal engineering, further highlighting how many of the modifications of solids become unavailable upon dissolution.

In an interesting recent paper by Martins and Emmerling on this topic,^[182]^ the authors highlighted that the grinding of the active pharmaceutical ingredient carbamazepine (CBZ) with three dihydroxybenzoic acids (2,4‐DHBA, 2,5‐DHBA, 2,6‐DHBA) delivered distinct crystal structures for each carboxylic acid used in the reaction (CBZ:2,4‐DHBA 1 : 1, CBZ:2,5‐DHBA 1 : 1, and CBZ:2,6‐DHBA 1 : 1, Figure 19). These studies have proved the existence of an empirical correlation between the type of hydrogen bond interactions present in the DHBA derivatives and the rate of co‐crystallization.

**Figure 19:**
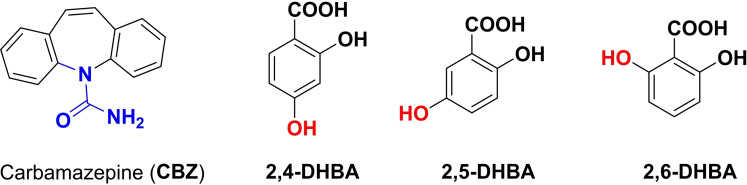
Chemical structure of CBZ and coformers 2,4‐, 2,5‐, and 2,6‐dihydroxybenzoic acids (DHBA).

**Figure 20:**
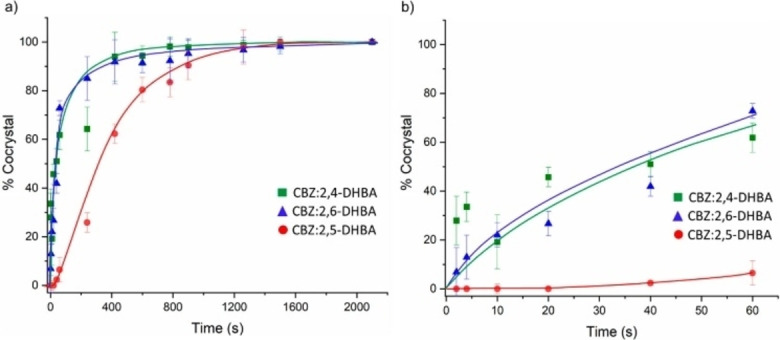
(a) Quantitative phase analysis for the co‐crystallization of CBZ with 2,4‐DHBA, 2,5‐DHBA, and 2,6‐DHBA obtained by Rietveld refinements, showing the cocrystal formation with the relative ESD bars. (b) Graphical expansion of the quantitative phase analysis plot. No fitting was performed, and the curves in the kinetic data are only a guide to the eye. Each point in time in these kinetic plots corresponds to an independent grinding experiment. Reproduced with permission from Ref. [182]. Copyright 2021, American Chemical Society.

They observed that the rate of co‐crystallization of CBZ:2,4‐DHBA and CBZ:2,6‐DHBA was faster than the formation of the CBZ:2.5‐DHBA cocrystal, reaching equilibrium after 600 s of milling without an induction period. In contrast, the formation of CBZ:2.5‐DHBA cocrystals presents a kinetic profile considerably different, whereby the reaction starts after a short induction period of 20 s and equilibrium is reached after 1300 s (Figure 20). Interestingly, the co‐crystallization kinetic profiles using different aromatic coformers, where the position of a functional group changed from *ortho* to *para*, were not explored so far. In this study, the authors found a close empirical correlation between the rate of co‐crystallization and the structure of the three different coformers. The slowest co‐crystallization rate was observed for CBZ:2.5‐DHBA, where, with respect to the hydrogen‐bonds motif of reagents, the major structural modifications in the cocrystal product were found. These noteworthy findings pointed out how different structurally related coformers can affect the kinetics of mechanochemical co‐crystallizations.

### 2.11 Solvent‐free solid‐state vs. liquid‐phase synthetic procedures

For various reasons, past and contemporary chemistry, including historical, is solvent‐based chemistry. Today, with its strong relationship with green chemistry, mechanochemistry is having an enormous success in changing the way we think about chemistry, significantly promoting solvent‐free reactions. Solvent‐free reactions, especially those involving solid components, are not the domain and subject of study of mechanochemistry exclusively.

**Figure 21:**
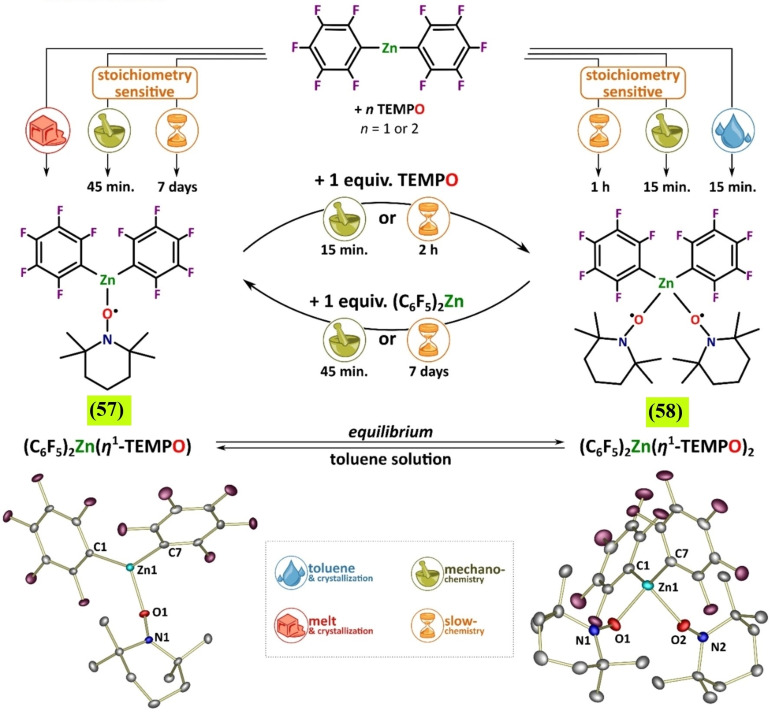
Speciation of the reaction products involving the Ar_2_Zn/TEMPO system for different Ar substituents and synthetic procedures. Reproduced with permission from Ref. [183a]. Copyright 2021.

Recently, Lewiński and co‐workers^[183]^ investigated the reaction between 2,2,6,6‐tetramethylpiperidinyl oxide (TEMPO) and the diamagnetic bis(pentafluorophenyl)zinc, (C_6_F_5_)_2_Zn, by conducting comparative studies in four different reaction media: the solid‐state mechano‐ and slow‐chemistry synthesis, melted phase, and solution protocols (Figure 21). These investigations go beyond conventional disciplinary boundaries solvent‐free solid‐state versus liquid‐phase synthetic approaches, validating the data with several analytical techniques [NMR spectroscopy, electron paramagnetic resonance (EPR) spectroscopy, PXRD, single‐crystal X‐ray, and density functional theory calculations]. The reaction carried out in toluene provided two distinct paramagnetic Lewis acid‐base adducts (C_6_F_5_)_2_Zn(η1‐TEMPO) (**57**) and (C_6_F5)_2_Zn(η1‐TEMPO)_2_ (**58**). The paramagnetic compound **58** is the only adduct isolated at −20 °C, irrespective of the molar ratios of the two starting materials. All experiments performed in the melt phase at 120 °C (sealed Schlenk flask) using an equimolar quantity of both reagents, sole yellow crystals of **57**, were obtained after slowly cooling to room temperature. Instead, in the case of a melted phase reaction where TEMPO and (C_6_F_5_)_2_Zn were in a 2 : 1 molar ratio, the PXRD analysis revealed the powder mixture consisted of complexes **57** and **58**. In contrast, the slow‐chemistry and mechanochemistry approaches were sensitive to the stoichiometry ratio and allowed for the selective synthesis of both adducts by simple stoichiometric control over the reactive components.

Noteworthy it is the dichotomy between the kinetics of the solid phase processes with one or two TEMPO equivalents. These are even more dramatic when conducted in the absence of an external stimuli (slow chemistry). In the low‐energy slow‐chemistry approaches using (C_6_F_5_)_2_Zn and TEMPO in 1 : 1 and 1 : 2 molar ratio, a complete conversion to the adduct **57** was achieved only after seven days, while the same reaction with 2 equiv. of TEMPO gave the adduct **58** in just 1 h. The authors attributed these two different kinetic profiles to a localized liquefaction process when an increased amount of TEMPO (2 equiv.) was added.

### 2.12 Mechanoradical reactions

Due to their unique mechanical properties, few materials have improved the quality of life and life expectancy in a human beings like synthetic polymers. From the beginning, it was clear that understanding how external mechanical stresses affected the stability of these polymeric materials would provide fundamental elements for improving their performance. Studies designed to understand how stress factors affect the polymer chain could contribute to develop more advanced polymeric functional materials.^[184]^ As early as 1930, Staundiger et al.^[185]^ realized that milling polystyrene was responsible for a significant decrease in molecular weight in the bulk state. Succeeding research efforts have shown that many of these and other correlated effects are a consequence of the homolytic cleavage of covalent bonds and the formation of radicals within strained polymers.^[186]^ Although many studies have demonstrated the formation of mechanoradicals by homolytic cleavage of the polymer chains in solution, polymeric mechanoradicals are not stable in the bulk state to be easily detected.^[187]^

**Figure 22:**
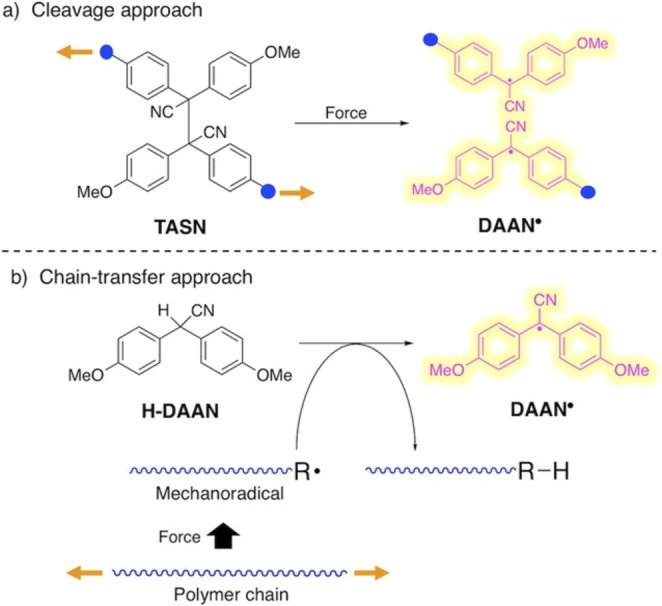
Generation of fluorescent diarylacetonitrile radical (DAAN_°_) (a) by the cleavage of tetraarylsuccinonitrile (TASN) incorporated in a polymer chain (cleavage approach) and (b) by the chain‐transfer reaction of diarylacetonitrile (H‐DAAN) by a polymer mechanoradical (chain‐transfer approach). Reproduced with permission from Ref. [188]. Copyright 2020, Wiley‐VCH.

In a recent paper, Otsuka and co‐workers^[188]^ developed an interesting procedure to detect and quantitatively evaluate polymer‐chain scission due to the formation of polymeric mechanoradicals. This innovative strategy is based on a radical scavenger that generates relatively stable fluorescent radicals (Figure 22). The generation of radicals can be directly observed in the bulk by EPR and fluorescence measurements. Diarylacetonitrile (H‐DAAN), a precursor for tetraarylsuccinonitrile (TASN), working as a radical‐type mechanochromophore, can be used as a molecular probe to detect mechanoradicals in the bulk state.

**Figure 23:**
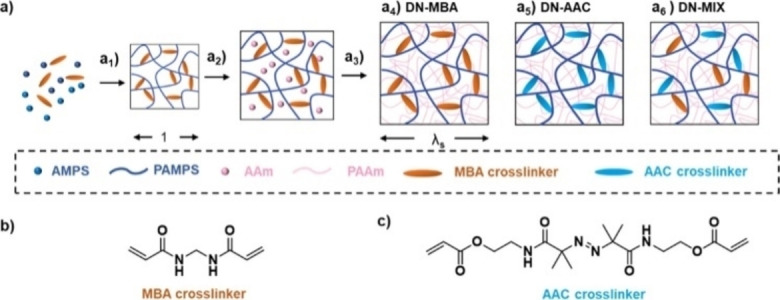
Preparation of DN gels with different crosslinkers. (a) By two step sequential polymerization (a_1_–a_3_) and varying the crosslinker in the first step of polymerization (a_1_), DN gels with different crosslinkers (a_4_–a_6_) are synthesized: (a_1_) synthesizing PAMPS SN gels, (a_2_) immersing PAMPS SN gels in an acrylamide (AAm) solution, and (a_3_) synthesizing the second Aam network in PAMPS gels and then immersing them into pure water to obtain equilibrated DN gels (a_4_–a_6_). The length swelling ratio *λ*_s_ of the PAMPS network in DN gels is determined by the thickness of the equilibrated DN gels (a_4_–a_6_) divided by the thickness of the as‐prepared PAMPS SN gels. (b,c) Chemical structure of MBA (b) and AAC (c) crosslinkers. Reproduced with permission from Ref. [190]. Copyright 2022, American Chemical Society.

Most artificial materials can be considered close and static; on the contrary, living things are open and dynamic “growing” materials, constantly upgraded using building blocks such as amino acids acquired from their environment. Gong and co‐workers^[189]^ have recently developed self‐growing double‐network (DN) hydrogels induced by mechanical straining to address these issues (Figure 23). DN gels are composed of two interpenetrating polymer networks with opposing mechanical properties. In the double‐network hydrogels, an external force can generate numerous radicals by the homolytic bond scission of the fragile first network. In these “growing” polymeric materials, the mechanical action promotes the formation of the mechanoradicals that trigger polymerization inside the gels, significantly improving the mechanical and functional properties of the resulting material.

In a very interesting paper, Gong and co‐workers^[190]^ successfully prepared a network of DN gels incorporating an azoalkane crosslinker (AAC, Figure 23). The azoalkane crosslinker leads to a decrease in yield stress, but also an excellent performance in generating mechanoradicals after stretching compared to the commercial crosslinker *N*,*N′*‐methylenebis(acrylamide). In these azoalkane‐crosslinked DN gels, the concentration of mechanoradicals is five times (up to ≈220 μm) that of the commercial crosslinker. In addition, the AAC crosslinker improved the energy efficiency for mechanoradical generation, increasing the radical generation performance, which in turn accelerates polymerization. In addition, the AAC crosslinker improves the energy efficiency for mechanoradical generation, increasing the radical generation performance, which in turn force‐triggers polymerization. The increased mechanoradical concentration is beneficial to broaden the application range of force‐responsive DN gels to biomedical device and soft robots.

**Figure 24:**
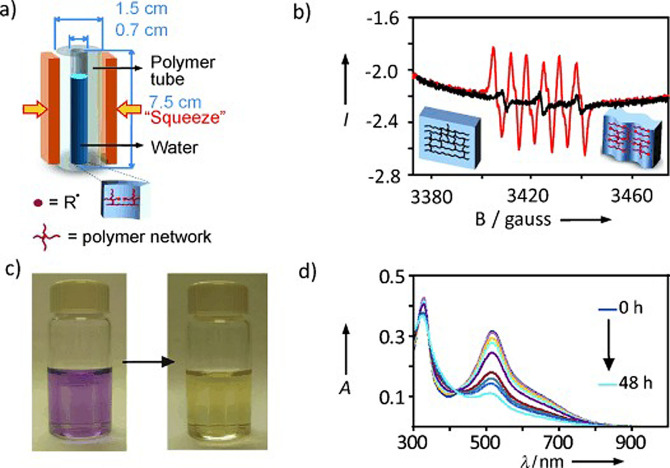
(a) Tubes made of PDMS, Tygon, or PVC are filled with deionized water and are compressed by a vice. (b) Electron spin resonance spectrum of a solution of DMPO in THF (1 mg mL^−1^) in the presence of compressed PDMS in an argon atmosphere (red) or uncompressed PDMS (black, the same spectrum is obtained in the absence of any PDMS). (c) A solution of DPPH (10.0  μM) changes color from purple to yellow when squeezed polymer tubes (in this case, Tygon) are added, which indicates the transformation from DPPH into DPPH−R in the presence of radicals. (d) Changes in the UV/Vis spectra of the solution of DPPH are shown over time. Reproduced with permission from Ref. [191]. Copyright 2012, Wiley‐VCH.

In this framework, Grzybowski and co‐workers^[191]^ have recently disclosed how a significant fraction of mechanochemical energy can be retrieved in polymers that are deformed and in contact with water (Figure 24). In these experiments, the authors observed the efficiency of mechanical‐to‐chemical energy conversion by the expenditure of 1 J energy input as high as 30 %, depending on the polymer used. Under these conditions, the mechanoradicals generated along the polymer matrix migrate to the polymer/water interface, producing H_2_O_2_ (up to tens of milligrams per square meter for 1 J of mechanical energy input), which can then promote several laboratory‐scale, aqueous‐phase, radical reactions, such as nanoparticle synthesis, dye bleaching, or fluorescence. Deformable soft, “spongy” polymers can be considered solid‐state chemical reagents that convert mechanical energy into chemical energy in a “clean”, environmentally friendly fashion.

### 2.13 Miscellaneous

In the Introduction, we pointed out that mechanochemistry is experiencing a remarkable breakthrough in several areas of chemistry. We are aware that the subjects covered in this Review are not exhaustive for this topic, and we have restricted our attention to highlighting the research areas closest to the authors’ knowledge to address them better. The reader interested in learning more about other topics closely related to mechanochemistry can find some interesting insights in the references.^[192]^

## 3 Scale‐Up: Twin‐Screw Extrusion

The processes described so far have highlighted the tremendous potential of mechanochemistry. This methodology does not only open new horizons for reactions that are often limited by their poor solubility in a solvent medium, but it provides the opportunity to design synthetic protocols with minimum production of waste and minimum consumption of energy. One of the most exciting features often overlooked is that mechanochemical processes can be easily scale‐up. Synthetic processes developed in a planetary mill have made it possible to operate on a scale in the order of several hundred grams.^[193]^

**Figure 25:**
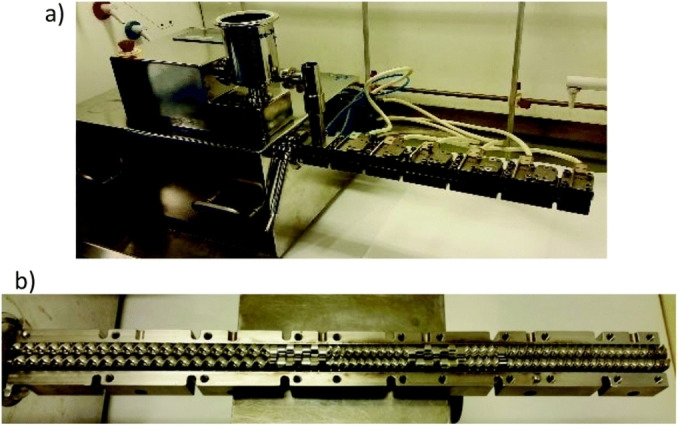
(a) Twin screw extruder. (b) Intermeshing co‐rotating screws encased in the extruder barrel. Reproduced with permission from Ref. [194a]. Copyright 2017, Royal Society of Chemistry.

Although industrial‐scale ball mills can efficiently work on larger scales (tons), they are mainly dedicated to material processing and have never been used in synthetic processes. Recently, twin‐screw extruders (TSE), widely used in the food, polymer, and pharmaceutical industries, have allowed mechanochemical processes to take a quantum leap forward, opening the door to many industrial applications in different manufacturing areas. TSE is a machine consisting of two intermeshing, co‐ or counter‐rotating screws mounted on splined shafts in a closed barrel and involves conveying the material through a confined space (Figure 25). Overall, this technology allows an easier scaling up of mechanochemical processes^[194]^ and better control of other process parameters, including temperature.

In addition, extruders are extremely versatile devices, so it is possible to heat various sections at different temperatures along their longitudinal section. This modular temperature control also enables the development of multistep processes, feeding the extruder at different points over the entire length of the process section. In addition, the endless screw that ensures shear and compressive forces to the material as it moves along the extruder barrel can be customized with conveying, kneading and reverse conveying segments to meet the specific needs of a given process. The average residence time of the material inside the extruder is a few minutes but can be prolonged according to the demands of the process. In most cases, TSE allows achieving high throughput rates (≈0.5 kg h^−1^) and space‐time yields (STY, up to 260×10^3^ kg m^−3^ day^−1^)^[194]^ without requiring additional energy‐consuming post purification processes. The screws are tight and self‐wiping, eliminating stagnant zones, resulting in high efficiency and a perfect self‐cleaning device. The considerable potential of this methodology was previously shown for the continuous manufacturing of hydrazone‐based active pharmaceutical ingredients^[13]^ and in the preparation of commodity chemicals such as perylene diimides.^[193]^ More recently, mechanochemical methods, including continuous flow mechanochemistry by TSE, for the preparation of APIs, have been quantitative assessed against the “12 Principles of Green Chemistry” by Colacino and co‐workers^[14b]^ and in life cycle assessment studies (LCA) by Spatari and co‐workers.^[91]^ A more detailed description of this technique and its applications in synthetic processes is available in a recent comprehensive Review article by Browne and co‐workers.^[195]^

## 4 Conclusion

Nowadays, many mechanochemical processes focus on a comparative study of procedures already widely known in solution. Although some classical approaches’ mechanochemical activation results are remarkable, they are still challenging to rationalize with current knowledge coming from chemistry in solution. This is like drawing the moon's dark side with pictures taken from the moon's surface visible from Earth.

Many of these challenges are closely connected with the complexity of monitoring the reaction's progress in real‐time to precisely understand what is happening inside the jar. The *in‐situ* methodology is currently the only available means to obtain direct insight into an uninterrupted mechanochemical reaction. These aspects have recently been covered in three exhaustive Reviews^[196]^ and hence will not be revisited in more details here.

Beyond this, mechanochemistry brings with itself a radical change in the panorama of organic synthesis, a field where changes typically proceed at a very slow pace. The different case studies and examples presented and discussed in this Review strongly re‐affirm the enormous potential of mechanochemistry to beneficially impact our society on levels ranging from pure scientific knowledge to environment and society. This Review, which is far from exhaustive, aims to show that mechanochemistry is many things simultaneously: a technology to drastically reduce the ecological footprint of a reaction, a strategy to synthesize compounds or materials that are not easily accessible by other means, but above all, it is a click still full of many unanswered questions. It is our desire that this study could inspire further research aiming at exploring the less‐known aspects of a stimulating area of investigation, thus contributing to further progress in the field.

## Author Contributions

The manuscript was written through the contributions of all authors. All authors have given approval to the final version of the manuscript. E.C. and A.P. contributed equally.

### Funding Sources

Financial support from FIR 2020, Fondazione di Sardegna (FdS, F72F20000230007), and MIUR (Italy, PRIN project: MultIFunctional poLymer cOmposites based on groWn matERials, n° 2017B7MMJ5_001) are gratefully acknowledged.

## Conflict of interest

The authors declare no conflict of interest.

## Biographical Information

Evelina Colacino is an Associate Professor at the University of Montpellier (France). Her research interests focus on the development of eco‐friendly methodologies to prepare value‐added compounds by mechanochemistry, with a main focus on active pharmaceutical ingredients (medicinal mechanochemistry). She promotes sustainability in higher education by integrating green chemistry at undergraduate level in organic chemistry courses, teaching laboratories and across the sub‐disciplines of chemistry, with a special focus on the fundamentals and the practice of mechanochemistry. She leads the European Programme COST Action CA18112 (MechSustInd) – ‘Mechanochemistry for Sustainable Industry (www.mechsustind.eu and https://www.cost.eu).

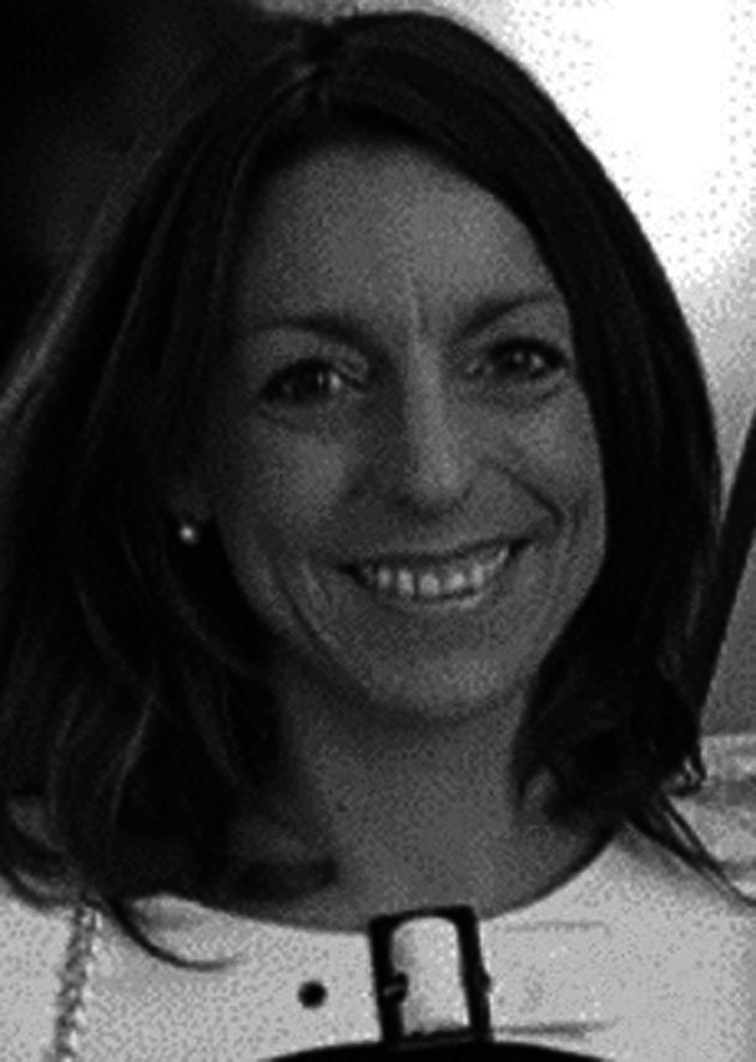

## Biographical Information

Andrea Porcheddu is a Full Professor at the University of Cagliari (Italy). He completed doctoral work at the University of Sassari before taking up a postdoctoral position at the Louis Pasteur University in Strasbourg (France). His diverse research activities range from synthesising chimaera molecules possessing complex molecular architecture to environmentally friendlier alternatives for synthesis using the most advanced technologies. Currently, his main interest is directed toward developing unconventional green procedures via ball milling. He is the author or co‐author of more than 110 scientific publications.